# Synthesis and Antiviral Activity of Novel 1,4-Pentadien-3-one Derivatives Containing a 1,3,4-Thiadiazole Moiety

**DOI:** 10.3390/molecules22040658

**Published:** 2017-04-21

**Authors:** Lu Yu, Xiuhai Gan, Dagui Zhou, Fangcheng He, Song Zeng, Deyu Hu

**Affiliations:** State Key Laboratory Breeding Base of Green Pesticide and Agricultural Bioengineering, Key Laboratory of Green Pesticide and Agriculture Bioengineering, Ministry of Education, Guizhou University, Guiyang 550025, Guizhou, China; yuji570@163.com (L.Y.); gxh200719@163.com (X.G.); zdg18230826051@163.com (D.Z.); hfc13667177168@163.com (F.H.); zengsong123@126.com (Z.S.)

**Keywords:** 1,4-pentadien-3-one derivatives, 1,3,4-thiadiazole, synthesis, anti-TMV, anti-CMV

## Abstract

1,4-Pentadien-3-one derivatives derived from curcumin possess excellent inhibitory activity against plant viruses. On the basis of this finding, a series of novel 1,4-pentadien-3-one derivatives containing a 1,3,4-thiadiazole moiety were designed and synthesized, and their structures confirmed by IR, ^1^H-NMR, and ^13^C-NMR spectroscopy and elemental analysis. The antiviral activities of the title compounds were evaluated against tobacco mosaic virus (TMV) and cucumber mosaic virus (CMV) in vivo. The assay results showed that most of compounds had remarkable antiviral activities against TMV and CMV, among which compounds **4b**, **4h**, **4i**, **4k**, **4o**, and **4q** exhibited good curative, protection, and inactivation activity against TMV. Compounds **4h**, **4i**, **4k**, **4l**, **4o**, and **4q** exhibited excellent protection activity against TMV, with EC_50_ values of 105.01, 254.77, 135.38, 297.40, 248.18, and 129.87 μg/mL, respectively, which were superior to that of ribavirin (457.25 µg/mL). In addition, preliminary SARs indicated that small electron-withdrawing groups on the aromatic ring were favorable for anti-TMV activity. This finding suggests that 1,4-pentadien-3-one derivatives containing a 1,3,4-thiadiazole moiety may be considered as potential lead structures for discovering new antiviral agents.

## 1. Introduction

**Figure 1:**
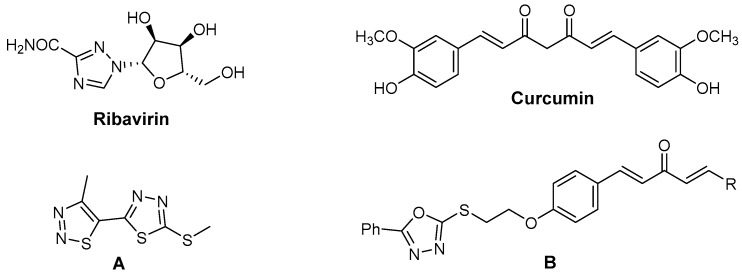
The structures of some antiviral agents and synthesized compounds.

Tobacco mosaic virus (TMV) and cucumber mosaic virus (CMV) are two important plant viruses, which can infect at least 125 species of nine plant families and cause significant economic losses in various crops, including tobacco, tomato, pepper, cucumbers, and a number of ornamental flowers [1,2]. Ribavirin (Figure 1) is widely used for preventing plant viruses, however, its inhibitory activities against TMV and CMV are less than 50% at 500 μg/mL [3]. In recent years, biologists have made great efforts to develop novel and effective antiviral compounds [4,5], but few compounds with high inhibition against plant virus have been found. That is to say, there are no efficient antiviral agents that can absolutely inhibit plant virus [6], so the development of new effective antiviral agents remains a significant challenge.

Natural products are important sources for drug development, and some natural products and their derivatives, such as limonoids [7], quassinoids [8], xanthones [9], antofine and its derivatives [10,11], as well as phenanthroindolizidine and its analogues [6], show excellent antiviral activity. Therefore, it is a development trend of modern agrochemical research to design and synthesize pesticides based on natural products. Curcumin (Figure 1), a member of a small family of natural products, exhibits extensive biological activities, including antibacterial [12], anticancer [13,14], and antiviral properties [15,16]. 1,4-Pentadien-3-one derivatives derived from curcumin possess numerous potential biological activities and play an important role in the discovery of new antiviral molecules. Our group has designed and synthesized a number of such 1,4-pentadien-3-one derivatives [17,18,19,20,21,22], and most of them exhibited excellent antiviral activities against TMV and CMV.

1,3,4-Thiadiazole is a potent multi-targeting pharmacological scaffold in heterocyclic chemistry [23]. 1,3,4-Thiadiazole derivatives have various biological activities, such as antimicrobial [24], antitubercular [25], anticonvulsants [26], antibacterial [27], anti-inflammatory [28,29], anticancer [30,31], antinociceptive [32], enzyme inhibitory [33], antidepressant and anxiolytic [34] effects. In our previous work, a number of 5-(4-chlorophenyl)-1,3,4-thiadiazole sulfonamides were designed and synthesized, and these compounds showed moderate anti-TMV activities [35]. In addition, 1,3,4-thiadiazole thioether derivative **A** (Figure 1) was found to display remarkable antiviral activity against TMV, and the results indicated that the thioethers exhibited better antiviral activity than sulfonamides [36].

In the further development of antiviral agents, a series of novel 1,4-pentadien-3-one derivatives containing a 5-phenyl-1,3,4-oxadiazole moiety (**B**, Figure 1) was found to have excellent anti-TMV activity [37]. In this study, we aimed to use a phenyl-1,3,4-thiadiazole to replace the phenyl-1,3,4-oxadiazole system to build novel 1,4-pentadien-3-one derivatives containing a 1,3,4-thiadiazole moiety for the development of antiviral agents. The assay results showed that most of title compounds exhibited good antiviral activity, among which compounds **4b**, **4h**, **4i**, **4k**, **4o**, and **4q** exhibited good curative, protection, and inactivation activity against TMV. The structure-activity relationships (SAR) of the compounds are also discussed. To the best of our knowledge, this is the first report on the synthesis and antiviral activity evaluation of 1,4-pentadien-3-one derivatives containing a 1,3,4-thiadiazole moiety.

## 2. Results and Discussion

### 2.1. Chemistry

**Scheme 1:**
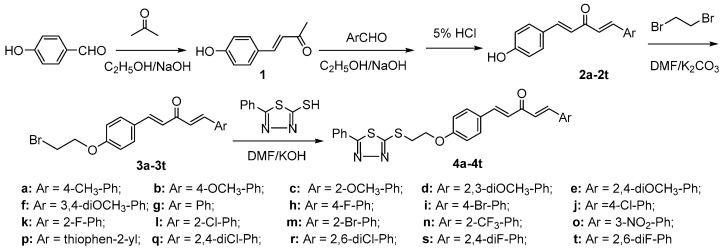
Synthesis of the title compounds **4a**–**4t**.

A synthetic route to 1,4-pentadien-3-one derivatives containing a 1,3,4-thiadiazole moiety was designed and is shown in Scheme 1. According to previously reported methods [38], 5-phenyl-1,3,4-thiadiazole-2-thiol could be obtained. The (1*E*,4*E*)-1-(4-(2-bromoethoxy)phenyl)-5-substitued phenylpenta-1,4-dien-3-ones **3a**–**3t** were prepared from 4-hydroxybenzaldehyde and aromatic aldehydes via reported procedures [22,37].

To obtain the target compounds in high yield, the reaction solvent, catalyst and temperature for the synthesis of compound **4a** were optimized on the basis of the molar ratio of 5-phenyl-1,3,4-thiadiazole-2-thiol:base:**3a** = 1.05:1.2:1.0, after a reaction time of 4 h, and the results are outlined in Table 1. It was shown that the maximum yield of **4a** (up to 87%) was achieved when the solvent, base and temperature were DMF, KOH and 40 °C, respectively. The other compounds were then synthesized under these conditions.

The structures of the target compounds were confirmed by IR, ^1^H-NMR, and ^13^C-NMR spectroscopy and elemental analysis. The data of **4a** are shown and discussed below. The IR spectra exhibited characteristic absorption bands at 3435 cm^−1^, which indicate the presence of a =CHCOCH= grouping. The stretching frequency at 2942–2838 cm^−1^ was assigned to CH vibrations. The characteristic absorptions at 1627 cm^−1^, 1610 cm^−1^, and 1598–1448 cm^−1^ were attributed to the presence of C=O, C=N, and C=C group vibrations, respectively. The characteristic absorption at 1108 cm^−1^ was assigned to CH vibrations. In the ^1^H-NMR, the four low-frequency downfield doublets at *δ* 7.71 (d, 1H, *J* = 16.0 Hz), 7.69 (d, 1H, *J* = 16.0 Hz), 7.03 (d, 1H, *J* = 16.0 Hz), and 6.97 (d, 1H, *J* = 15.0 Hz) ppm revealed the presence of four *trans* =C-H protons. The various absorption peaks at *δ* 7.89–6.98 ppm indicated the presence of phenyl protons. The triplets at 4.77 and 3.80 ppm indicated the presence of -O-CH_2_- and –S-CH_2_- absorption peaks. The presence of Ar-CH_3_ showed a singlet at 2.39 ppm. The typical chemical shifts near *δ* 189.03, 168.81, 164.50, and 160.35 ppm of ^13^C-NMR spectra validated the presence of C=O, C-N (two), and C-O, respectively. Meanwhile, peaks near *δ* 66.33 and 32.69 ppm confirmed the presence of -OCH_2_- and -SCH_2_-. The typical peak near *δ* 21.64 ppm also verified the presence of -ArCH_3_. ESI-HRMS (*m*/*z*), calcd. for C_28_H_24_O_2_N_2_NaS_2_ [M + Na]^+^ 507.11714, found 507.11646.

### 2.2. Antiviral Activity of Title Compounds against TMV In Vivo

The antiviral activities of the title compounds **4a**–**4t** against TMV were evaluated by the half-leaf method [39] and the results were summarized in Table 2. It was found that most of the title compounds exhibited good antiviral activity against TMV in vivo. Compounds **4a**, **4b**, **4g**, **4h**, **4i**, **4k**, **4o**, **4q**, and **4t** showed remarkable curative activity against TMV, with values of 55.8, 56.4, 56.3, 56.2, 53.7, 56.5, 51.7, 58.7 and 54.5%, respectively, which were better than that of ribavirin (37.9%). Meanwhile, compounds **4b**, **4f**, **4h**, **4i**, **4k**, **4l**, **4o**, and **4q** exhibited excellent protection activity, also superior to ribavirin (51.8%). Overall, most of the compounds except **4d** indicated significant inactivation activity against TMV at 500 µg/mL.

Based on the previous bioassays, the 50% effective concentration (EC_50_) values of protection activities against TMV of the title compounds were tested and are listed in Table 2. Most of the target compounds showed good antiviral activity against TMV. Compounds **4h**, **4i**, **4k**, **4l**, **4o**, and **4q** exhibited excellent protection activity against TMV, with the EC_50_ values of 105.01, 254.77, 135.38, 297.40, 248.18 and 129.87 μg/mL, respectively, which were better than that of ribavirin (457.25 µg/mL). In summary, we found that the compounds **4b**, **4h**, **4i**, **4k**, **4o**, and **4q** had good curative, protection, and inactivation activity against TMV.

### 2.3. Antiviral Activity of Title Compounds against CMV In Vivo

The antiviral activities of the title compounds **4a**–**4t** against CMV were tested by the half-leaf method [22] and the results are summarized in Table 3. Some of the title compounds exhibited good antiviral activity against CMV in vivo. Compounds **4e**, and **4f** showed remarkable curative activity against CMV, with values of 55.9% and 50.2%, respectively, which were better than that of ribavirin (36.8%). Meanwhile, the protection and inactivation activity of the target compounds was similar to that of ribavirin.

### 2.4. Antiviral Activity and Structure-Activity Relationships

The antiviral bioassay results indicated that the target compounds showed excellent antiviral activity against TMV. The preliminary SAR results were deduced on the basis of the anti-TMV activity (as shown in Table 2). The results showed that when the Ar is 4-OCH_3_-Ph (**4b**), 3,4-diOCH_3_-Ph (**4f**), 4-*F*-Ph (**4h**), 4-*Br*-Ph (**4i**), 2-*F*-Ph (**4k**), 2-*Cl*-Ph (**4l**), 3-NO_2_-Ph (**4o**), and 2,4-diCl-Ph (**4q**) groups, the corresponding target compounds exhibited good protection activity. Moreover, the results showed that electron-withdrawing groups on aromatic rings were favorable for antiviral activity at the same position, these findings were confirmed by the following activity order **4h** (Ar = 4-*F*-Ph) > **4i** (Ar = 4-*Br*-Ph) > **4a** (Ar = 4-CH_3_-Ph), **4k** (Ar = 2-*F*-Ph) > **4l** (Ar = 2-*Cl*-Ph) > **4c** (Ar = 2-OCH_3_-Ph), and **4q** (2,4-diCl-Ph) > **7e** (2,4-diOCH_3_-Ph). Bulky group of aromatic rings disfavor antiviral activity, a notion supported by the activity order of **7b** (Ar = 4-OCH_3_-Ph) > **4e** (Ar = 2,4-diOCH_3_-Ph), **4h** (Ar = 4-*F*-Ph) > **4i** (Ar = 4-*Br*-Ph), and **4k** (Ar = 2-*F*-Ph) > **4l** (Ar = 2-*Cl*-Ph) > **4m** (Ar = 2-*Br*-Ph).

## 3. Materials and Methods

### 3.1. General Information

Melting points of the compounds were recorded on an XT-4 binocular microscope melting point apparatus (Beijing Tech Instruments Co., Beijing, China), and are uncorrected. Proton nuclear magnetic resonance (NMR) spectra were determined at 500 and 125 MHz using an ECX 500 NMR spectrometer (JEOL, Tokyo, Japan) in CDCl_3_ solvent, using TMS as an internal standard. Infrared (IR) spectra were obtained on a Vector 22 Fourier transform infrared (FTIR) spectrometer (Bruker, Karlsruhe, Germany) in KBr disks. Elemental analyses were determined on an Elementar Vario-III CHN analyzer (Elementar Analysensysteme GmbH, Frankfurt, Germany). High resolution mass spectrometer (HRMS) data was conducted using a Thermo Scientific Q Exactive (Thermo, Waltham, MA, USA). Reaction progress was monitored by thin-layer chromatography (TLC) on silica gel GF254. Column chromatographic purification was carried out using silica gel (200–300 mesh, Qingdao Bangkai Hi-Tech materials Co., Ltd. Qingdao, Shandong, China). All solvents and reagents were of analytical reagent grade or chemically pure, and the solvents were dried in advance and distilled before use.

### 3.2. Chemistry

#### 3.2.1. General Procedure for Preparation of Intermediates **3a**–**3t**

(*E*)-4-(4-hydroxyphenyl) but-3-en-2-one (**1**) was synthesized from 4-hydroxybenzaldehyde (20 mmol) and acetone (20 mL) via a room temperature Claisen-Schmidt aldol condensation. (1*E*,4*E*)-1-(4-Hydroxyphenyl)-5-substitued phenylpenta-1,4-dien-3-ones **2** were synthesized from **1** (4 mmol) and an aromatic aldehyde (4 mmol) at room temperature. Thus, to a solution of the corresponding **2** (2 mmol) in DMF (3 mL), potassium carbonate (4 mmol) was added, and the resulting solution was stirred at room temperature for 1 h. Then 1,2-dibromoethane (10 mmol) was added to the mixture, warmed to 80 °C and stirred for 6 h. Upon reaction completion (as indicated by TLC), the solid was removed by filtration, and *N*,*N*-dimethylformamide and excess 1,2-dibromoethane were evaporated under vacuum. The crude product was purified by silica-gel column chromatography using petroleum ether/ethyl acetate (5:1/*v*:*v*) as the eluant to give (1*E*,4*E*)-1-(4-(2-bromoethoxy)phenyl)-5-substitued phenylpenta-1,4-dien-3-ones **3a**–**3t** as faint yellow powders [22,37].

#### 3.2.2. General Procedure for Preparation of Title Compounds **4a**–**4t**

A mixture of 5-phenyl-1,3,4-thiadiazole-2-thiol (1.05 mmol) and potassium hydroxide (1.2 mmol) in DMF (4 mL) was stirred at room temperature for 1 h, and then a solution of the appropriate (1*E*,4*E*)-1-(4-(2-bromoethoxy)phenyl)-5-substitued phenylpenta-1,4-dien-3-one **3** (1.0 mmol) in DMF (4 mL) was added. The mixture was warmed to 50 °C and stirred continuously for 4 h to 6 h. Upon reaction completion (as indicated by TLC), after dropwise addition of cold brine, the solid was filtered off, and washed with cold water. Then the crude product was recrystallized from ethyl acetate, filtered, washed, and dried to obtain the title 1,4-pentadien-3-one derivatives. The physical characteristics, IR, ^1^H-NMR, ^13^C-NMR, and elemental analysis data, for all the synthesized compounds are provided in Supporting Information and the representative data of **4a** are listed below.

*(1E,4E)-1-(4-(2-((5-Bhenyl-1,3,4-thiadiazol-2-yl)thio)ethoxy)phenyl)-5-(p-tolyl)penta-1,4-dien-3-one* (**4a**). Faint yellow powder; m.p. 125–126 °C; yield 87%; IR (KBr, cm^−1^): *ν* 3435 (=CHCOCH=), 2942–2838 (CH), 1627 (C=O), 1610 (C=N), 1598–1448 (C=C and benzene), 1108 (-C-O-C); ^1^H-NMR: *δ* 7.89 (d, 2H, *J* = 8.6 Hz, 5’-Ar-2, 6-H), 7.71 (d, 1H, *J* = 16.0 Hz, 5-H), 7.69 (d, 1H, *J* = 16.0 Hz, 1-H), 7.57 (d, 2H, *J* = 8.5 Hz, 1-Ar-2, 6-H), 7.51 (d, 2H, *J* = 8.0 Hz, 5-Ar-2, 6-H), 7.49–7.48 (m, 3H, 5’-Ar-3, 4, 5-H), 7.22 (d, 2H, *J* = 8.0 Hz, 5-Ar-3, 5-H), 7.03 (d, 1H, *J* = 16.0 Hz, 2-H), 6.98 (d, 2H, *J* = 8.5 Hz, 1-Ar-3, 5-H), 6.97 (d, 1H, *J* = 15.0 Hz, 4-H), 4.47 (t, 2H, *J* = 6.5 Hz, -CH_2_O-), 3.80 (t, 2H, *J* = 6.5 Hz, -CH_2_S-), 2.39 (s, 3H, CH_3_); ^13^C-NMR: *δ* 189.03, 168.81, 164.50, 160.35, 143.08, 142.84, 141.00, 132.24, 131.28, 130.26, 130.26, 129.79, 129.79, 129.34, 129.34, 128.47, 128.47, 128.14, 127.82, 127.82, 124.78, 123.67, 115.15, 115.15, 66.33, 32.69, 21.64.; Anal. Calcd. for C_28_H_24_N_2_O_2_S_2_: C, 69.39; H, 4.99; N, 5.78; Found: C, 69.30; H, 5.01; N, 5.81. ESI-HRMS (*m*/*z*), calcd. for C_28_H_24_O_2_N_2_NaS_2_ [M + Na]^+^ 507.11714, found 507.11646.

### 3.3. Antiviral Biological Assay

#### 3.3.1. Purification of TMV and CMV

TMV and CMV were inoculated in *Nicotiana tabacum* cv. K326, and purified by the Gooding method [40].

#### 3.3.2. Curative Activities of Compounds against TMV and CMV In Vivo

TMV and CMV (at a concentration of 6 μg/mL) were inoculated on the whole leaves of the same growing leaves of *Nicotiana tabacum* L. After 1 h, the leaves were washed with water, and after drying, the compound solution was smeared on the left side of leaf and the solvent was smeared on the right side for control. The local lesion numbers were recorded 3 to 4 days after inoculation [22,39].

#### 3.3.3. Protective Activities of Compounds against TMV and CMV In Vivo

The solution of the compound was smeared on the left side of leaf, while the solvent was served as control on the right side of leaf. The leaves were inoculated with 6 μg/mL TMV and CMV after 12 h. Then, the leaves were washed with water. The number of local lesions numbers was counted after 3 to 4 days [22,39].

#### 3.3.4. Inactivation Activities of Compounds aga inst TMV and CMV In Vivo

TMV and CMV was inhibited by mixing with the compound solution at the same volume for 30 min, respectively. Then, it was inoculated on the left side of leaf, and the right side of the leaf was inoculated with solvent and virus mixture as the control. The local lesion numbers were recorded 3 to 4 days after inoculation [22,39]. The in vivo inhibition rates of the compound were calculated based on the following formula (“av” means average). Inhibition rate (%) = [(av local lesion no. of control − av local lesion no. smeared with drugs)/av local lesion no. of control] × 100%. Three replications were conducted for each compound.

## 4. Conclusions

In summary, a serial of 1,4-pentadien-3-one derivatives containing a 1,3,4-thiadiazole moiety were designed for the development of antiviral agents, and twenty novel compounds were synthesized using optimized reaction conditions. The assay results demonstrated that most of compounds exhibited remarkable antiviral activities against TMV and CMV, among which compounds **4b**, **4h**, **4i**, **4k**, **4o**, and **4q** exhibited good curative, protection, and inactivation activity against TMV. Especially, compounds **4h**, **4i**, **4k**, **4l**, **4o**, and **4q** displayed excellent protection activity against TMV, with the EC_50_ values of 105.01, 254.77, 135.38, 297.40, 248.18, and 129.87 μg/mL, respectively, which were better than that of ribavirin (457.25 µg/mL). Preliminary SARs illuminated that small electron-withdrawing groups on the aromatic ring were favorable for antiviral activity at the same position. This finding suggests that 1,4-pentadien-3-one derivatives containing a 1,3,4-thiadiazole moiety may be used as potential lead structures for development of new antiviral agents.

## Figures and Tables

**Table 1:** Effect of the different conditions for the synthesis of **4a**.

No.	Solvent	Base	Temperature (°C)	Yield (%) ^a^
1	DMF	Et_3_N	25	0
2	DMF	K_2_CO_3_	25	21
3	DMF	K_2_CO_3_	50	38
4	acetone	KOH	25	18
5	THF	KOH	25	26
6	CH_3_CN	KOH	25	38
7	DMF	KOH	25	56
8	DMF	KOH	40	87
9	DMF	NaOH	40	72

^a^ Yields of isolated products.

**Table 2:** Antiviral activities of the test compounds against TMV in vivo.

Compound	Curative Activity (%) ^a^	Protection Activity (%) ^a^	Inactivation Activity (%) ^a^	EC_50_ of Protection Activity (µg/mL) ^a^
**4a**	55.8 ± 3.1	55.9 ± 3.5	84.1 ± 4.8	344.23 ± 2.35
**4b**	56.4 ± 1.5	64.5 ± 1.1	84.1 ± 4.8	319.67 ± 1.89
**4c**	36.8 ± 0.6	52.2 ± 1.4	91.8 ± 3.8	411.42 ± 2.68
**4d**	21.2 ± 1.5	24.2 ± 2.1	64.3 ± 2.6	1058.25 ± 2.11
**4e**	45.0 ± 1.6	30.4 ± 2.1	82.4 ± 1.8	1042.09 ± 1.28
**4f**	40.6 ± 0.8	61.6 ± 3.3	90.1 ± 0.3	389.46 ± 2.32
**4g**	56.3 ± 2.7	46.3 ± 1.4	78.5 ± 1.9	592.44 ± 1.89
**4h**	56.2 ± 3.5	70.2 ± 1.3	93.8 ± 1.7	105.01 ± 3.15
**4i**	53.7 ± 1.6	64.3 ± 2.7	85.3 ± 2.0	254.77 ± 1.66
**4j**	44.6 ± 3.2	53.6 ± 3.7	85.2 ± 2.2	388.31 ± 2.05
**4k**	56.5 ± 2.1	68.4 ± 1.8	87.1 ± 3.6	135.38 ± 3.12
**4l**	45.9 ± 3.1	63.4 ± 4.4	83.0 ± 1.9	297.40 ± 4.10
**4m**	40.5 ± 1.8	54.8 ± 2.5	81.5 ± 1.2	334.03 ± 1.08
**4n**	47.9 ± 2.5	59.8 ± 4.3	89.3 ± 2.5	309.09 ± 2.56
**4o**	51.7 ± 2.8	64.8 ± 3.1	77.6 ± 1.3	248.18 ± 4.14
**4p**	24.2 ± 4.5	50.7 ± 1.5	88.6 ± 1.1	466.15 ± 1.98
**4q**	58.7 ± 3.0	68.4 ± 1.6	84.7 ± 2.6	129.87 ± 3.55
**4r**	42.3 ± 2.4	56.7 ± 2.8	87.4 ± 2.9	316.77 ± 2.54
**4s**	28.2 ± 3.8	59.0 ± 1.7	82.7 ± 1.6	316.52 ± 4.29
**4t**	54.5 ± 4.4	54.1 ± 1.2	82.7 ± 1.6	425.71 ± 3.17
Ribavirin ^b^	37.9 ± 1.9	51.8 ± 2.3	72.9 ± 2.4	457.25± 2.68

^a^ Average of three replicates, at 500 μg/mL. ^b^ The commercial antiviral agent.

**Table 3:** Antiviral activities of the test compounds against CMV in vivo.

Compound	Curative Activity (%) ^a^	Protection Activity (%) ^a^	Inactivation Activity (%) ^a^
**4a**	29.3 ± 1.3	40.1 ± 2.5	71.3 ± 3.1
**4b**	23.6 ± 2.2	38.9 ± 2.9	65.2 ± 2.5
**4c**	37.9 ± 3.7	43.4 ± 3.1	61.5 ± 2.2
**4d**	18.5 ± 1.8	33.6 ± 2.1	51.2 ± 3.4
**4e**	55.9 ± 1.8	42.3 ± 1.1	79.6 ± 4.2
**4f**	50.2 ± 2.7	46.6 ± 2.3	62.5 ± 1.9
**4g**	39.8 ± 2.5	48.5 ± 2.7	66.7 ± 2.2
**4h**	39.8 ± 2.5	36.2 ± 2.2	62.1 ±1.9
**4i**	37.3 ± 2.5	35.6 ± 2.1	51.8 ± 1.1
**4j**	40.6 ± 3.7	45.9 ± 1.6	79.1± 2.5
**4k**	26.4 ± 2.3	35.6 ± 1.9	49.9± 3.6
**4l**	29.7 ± 1.1	43.5 ± 2.5	66.5 ± 2.0
**4m**	28.5 ± 2.3	42.2 ± 1.4	68.9 ± 2.8
**4n**	18.9 ± 2.9	25.4 ± 1.7	46.2 ± 0.9
**4o**	31.8 ± 2.8	49.9 ± 1.9	58.2 ± 3.2
**4p**	29.5 ± 1.4	38.5 ± 2.1	59.8 ± 1.9
**4q**	44.5 ± 1.8	48.1 ± 2.4	43.9 ± 1.2
**4r**	26.4 ± 2.2	40.5 ± 1.9	42.5 ± 2.8
**4s**	41.0 ± 1.7	54.7 ± 2.4	78.5 ± 1.9
**4t**	34.8 ± 2.3	48.1 ± 1.6	69.5 ± 2.0
Ribavirin ^b^	36.8 ± 1.6	47.9 ± 2.7	71.2 ± 1.7

^a^ Average of three replicates, at 500 μg/mL. ^b^ A commercial antiviral agent.

## Supplementary Materials

The following are available online. The data and spectrogram of compounds **4a**–**4t**.
