# Supplementary material for: Synthesis and Antiviral Activity of Novel 1,4-Pentadien-3-one Derivatives Containing a 1,3,4-Thiadiazole Moiety
Source: Molecules. 2017 Apr 21;22(4):658. doi: 10.3390/molecules22040658 (PMC6154619; doi:10.3390/molecules22040658)
Supplement: Supplementary file 1 [file molecules-22-00658-s001.pdf]

Supporting Information

Synthesis and Antiviral Activity of Novel 1,4-Pentadie-3-one Derivatives Containing a 1,3,4-Thiadiazol moiety

Lu Yu †, Xiuhai Gan †, Dagui Zhou, Fangcheng He, Song Zeng and Deyu Hu *

State Key Laboratory Breeding Base of Green Pesticide and Agricultural Bioengineering, Key Laboratory of Green Pesticide and Agriculture Bioengineering, Ministry of Education, Guizhou University, Guiyang 550025, China; E-Mails: yuji570@163.com (L.Y.); gxh200719@163.com (X.G.); zdg18230826051@163.com (D.Z.); hfc13667177168@163.com (F.H); zengsong123@126.com (Z.S.)

† These authors contributed equally to this work.

* Author to whom correspondence should be addressed; E-Mail: dyhu@gzu.edu.cn; Tel/Fax: +86-851-829-2170.

Content

1. The data of title compounds 4a–4t……………………………………………2

2. Spectrogram of title compounds 4a–4t………………………………………10

1. The data of title compounds 4a–4t

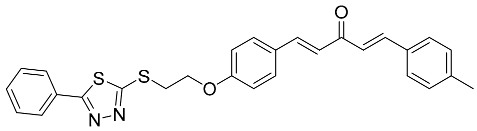

Data for *(1E,4E)-1-(4-(2-((5-phenyl-1,3,4-thiadiazol-2-yl)thio)ethoxy)phenyl)-5-(p-tolyl)penta -1,4-dien-3-one* (**4a**). Faint yellow powder; m.p. 125–126 °C; yield 87%; IR (KBr, cm^{-1}): ν 3435 (=CHCOCH=), 2942–2838 (CH), 1627 (C=O), 1610 (C=N), 1598–1448 (C=C and benzene), 1108 (–C–O–C); ^1H NMR (500 MHz, $CDCl_3$): δ 7.89 (d, 2H, J = 8.6 Hz, 5′-Ar-2, 6-H), 7.71 (d, 1H, J = 16.0 Hz, 5-H), 7.69 (d, 1H, J = 16.0 Hz, 1-H), 7.57 (d, 2H, J = 8.5 Hz, 1-Ar-2, 6-H), 7.51 (d, 2H, J = 8.0 Hz, 5-Ar-2, 6-H), 7.49–7.48 (m, 3H, 5′-Ar-3, 4, 5-H), 7.22 (d, 2H, J = 8.0 Hz, 5-Ar-3, 5-H), 7.03 (d, 1H, J = 16.0 Hz, 2-H), 6.98 (d, 2H, J = 8.5 Hz, 1-Ar-3, 5-H), 6.97 (d, 1H, J = 15.0 Hz, 4-H), 4.47 (t, 2H, J = 6.5 Hz, $-CH_2O-$), 3.80 (t, 2H, J = 6.5 Hz, $-CH_2S-$), 2.39 (s, 3H, CH_3); ^{13}C NMR (125 MHz, $CDCl_3$): δ 189.03, 168.81, 164.50, 160.35, 143.08, 142.84, 141.00, 132.24, 131.28, 130.26, 130.26, 129.79, 129.79, 129.34, 129.34, 128.47, 128.47, 128.14, 127.82, 127.82, 124.78, 123.67, 115.15, 115.15, 66.33, 32.69, 21.64. Anal. Calcd for $C_{28}H_{24}N_2O_2S_2$: C, 69.39; H, 4.99; N, 5.78; Found: C, 69.30; H, 5.01; N, 5.81. ESI-HRMS (m/z), calcd for $C_{28}H_{24}O_2N_2NaS_2$ $[M+Na]^+$ 507.11714, found 507.11646.

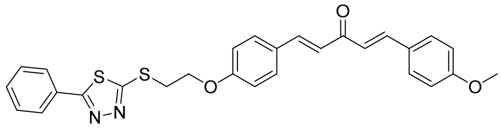

Data for *(1E,4E)-1-(4-methoxyphenyl)-5-(4-(2-((5-phenyl-1,3,4-thiadiazol-2-yl)thio)ethoxy) phenyl)penta-1,4-dien-3-one* (**4b**). Faint yellow powder; m.p. 131–132 °C; yield 78%; IR (KBr, cm^{-1}): ν 3435 (=CHCOCH=), 2943–2849 (CH), 1632 (C=O), 1601 (C=N), 1597–1452 (C=C and benzene), 1176 (–C–O–C); ^1H NMR (500 MHz, $CDCl_3$): δ 7.89–7.87 (m, 2H, 5′-Ar-2, 6-H), 7.70 (d, 1H, J = 16.0 Hz, 5-H), 7.68 (d, 1H, J = 16.0 Hz, 1-H), 7.56 (d, 4H, J = 7.5 Hz, 1,5-Ar-2, 6-H), 7.49–7.47 (m, 3H, 5′-Ar-3, 4, 5-H), 6.96 (d, 2H, J = 6.0 Hz, 1-Ar-3, 5-H), 6.95 (d, 1H, J = 11.0 Hz, 2-H), 6.94 (d, 1H, J = 11.0 Hz, 4-H), 6.92 (d, 2H, J = 6.5 Hz, 5-Ar-3, 5-H), 4.46 (t, 2H, J = 6.0 Hz, $-CH_2O-$), 3.85 (s, 3H, OCH_3), 3.79 (t, 2H, J = 6.0 Hz, $-CH_2S-$); ^{13}C NMR (125 MHz, $CDCl_3$): δ 188.92, 168.80, 164.50, 161.64, 160.29, 142.84, 142.60, 131.27, 130.23, 130.23, 130.20, 130.20, 129.84, 129.34, 129.34, 128.20, 127.82, 127.82, 127.69, 123.78, 123.59, 115.14, 115.14, 114.50, 114.50, 66.33, 55.51, 32.70. Anal. Calcd for $C_{28}H_{24}N_2O_3S_2$: C, 67.18; H, 4.83; N, 5.60; Found: C, 67.21; H, 4.81; N, 5.70. ESI-HRMS (m/z), calcd for $C_{28}H_{24}O_3N_2NaS_2$ $[M+Na]^+$ 523.11206, found 523.11157.

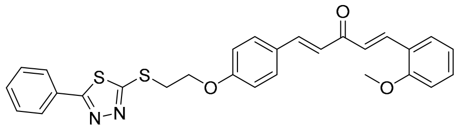

Data for *(1E,4E)-1-(2-methoxyphenyl)-5-(4-(2-((5-phenyl-1,3,4-thiadiazol-2-yl)thio)ethoxy) phenyl)penta-1,4-dien-3-one* (**4c**). Faint yellow powder; m.p. 135–136 °C; yield 85%; IR (KBr, cm^{-1}): ν 3435 (=CHCOCH=), 2933–2851 (CH), 1632 (C=O), 1611 (C=N), 1598–1457 (C=C and benzene), 1175 (–C–O–C); ^1H NMR (500 MHz, $CDCl_3$): δ 8.04 (d, 1H, J = 16.0 Hz, 5-H),

7.89–7.87 (m, 2H, 5′-Ar-2, 6-H), 7.69 (d, 1H, *J* = 15.0 Hz, 1-H), 7.60 (dd, 1H, *J* = 7.5, 1.5 Hz, 1-Ar-6-H), 7.57 (d, 2H, *J* = 8.5 Hz, 5-Ar-2,6-H), 7.49–7.47 (m, 3H, 5′-Ar-3, 4, 5-H), 7.37 (t, 1H, *J* = 7.5 Hz, 1-Ar-4-H), 7.11 (d, 1H, *J* = 16.5 Hz, 2-H), 6.99 (d, 1H, *J* = 15.5 Hz, 4-H), 6.99–6.92 (m, 4H, 1-Ar-3,5-H, 5-Ar-3,5-H,), 4.46 (t, 2H, *J* = 6.5 Hz, -CH_2O-), 3.92 (s, 3H, OCH_3), 3.79 (t, 2H, *J* = 6.5 Hz, -CH_2S-); ^{13}C NMR (125 MHz, $CDCl_3$): δ 189.53, 168.80, 164.50, 160.26, 158.67, 142.64, 138.34, 131.71, 131.27, 130.24, 130.24, 129.85, 129.33, 129.33, 128.89, 128.29, 127.82, 127.82, 126.65, 123.97, 123.55, 120.86, 115.13, 115.13, 111.26, 66.34, 55.63, 32.72. Anal. Calcd for $C_{28}H_{24}N_2O_3S_2$: C, 67.18; H, 4.83; N, 5.60; Found: C, 67.21.30; H, 4.92; N, 5.81. ESI-HRMS (m/z), calcd for $C_{28}H_{24}O_3N_2NaS_2$ [M+Na]$^+$ 523.11206, found 523.11151.

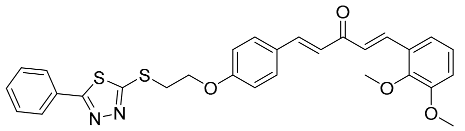

Data for *(1E,4E)-1-(2,3-dimethoxyphenyl)-5-(4-(2-((5-phenyl-1,3,4-thiadiazol-2-yl)thio)ethoxy) phenyl)penta-1,4-dien-3-one* (**4d**): Faint yellow powder; m.p. 143–145 ˚C; yield 79%; IR (KBr, cm^{-1}): ν 3439 (=CHCOCH=), 2943–2861 (CH), 1638 (C=O), 1601 (C=N), 1598–1454 (C=C and benzene), 1187 (–C–O–C); ^{1}H NMR (500 MHz, $CDCl_3$): δ 7.82 (d, 1H, *J* = 16.0 Hz 5-H), 7.88 (d, 2H, *J* = 8.0 Hz, 5′-Ar-2, 6-H), 7.69 (d, 1H, *J* = 15.0 Hz, 1-H), 7.57 (d, 2H, *J* = 8.5 Hz, 5-Ar-2,6-H), 7.48–7.45 (m, 3H, 5′-Ar-3, 4, 5-H), 7.24 (d, 1H, *J* = 8.0 Hz, 1-Ar-6-H), 7.10 (d, 1H, *J* =17.0 Hz, 2-H), 7.08 (t, 1H, *J* = 8.0 Hz, 1-Ar-5-H), 6.98 (d, 1H, *J* = 16.0 Hz, 4-H), 6.97 (d, 2H, *J* = 8.5 Hz, 5-Ar-3,5-H), 6.95 (d, 1H, *J* = 7.0 Hz, 1-Ar-4-H), 4.46 (t, 2H, *J* = 6.5 Hz, -CH_2O-), 3.87 (s, 6H, OCH_3), 3.79 (t, 2H, *J* = 6.5 Hz, -CH_2S-); ^{13}C NMR (125 MHz, $CDCl_3$): δ 189.31, 168.80, 164.48, 160.36, 153.27, 148.87, 142.97, 137.64, 131.26, 130.27, 130.27, 129.86, 129.32, 129.32, 129.23, 128.18, 127.82, 127.82, 127.31, 124.28, 123.54, 119.46, 115.17, 115.17, 114.12, 66.37, 61.49, 55.99, 32.72. Anal. Calcd for $C_{29}H_{26}N_2O_4S_2$: C, 65.64; H, 4.94; N, 5.28; Found: C, 65.51.30; H, 4.92; N, 5.32. ESI-HRMS (m/z), calcd for $C_{29}H_{26}O_4N_2NaS_2$ [M+Na]$^+$ 553.12262, found 553.12189.

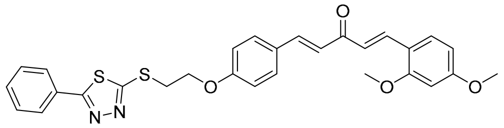

Data for *(1E,4E)-1-(2,4-dimethoxyphenyl)-5-(4-(2-((5-phenyl-1,3,4-thiadiazol-2-yl)thio)ethoxy) phenyl)penta-1,4-dien-3-one* (**4e**): Faint yellow powder; m.p. 144–145 ˚C; yield 79%; IR (KBr, cm^{-1}): ν 3438 (=CHCOCH=), 2941–2856 (CH), 1635 (C=O), 1609 (C=N), 1596–1451 (C=C and benzene), 1178 (–C–O–C); ^{1}H NMR (500 MHz, $CDCl_3$): δ 7.98 (d, 1H, *J* = 16.0 Hz, 5-H), 7.89–7.87 (m, 2H, 5′-Ar-2, 6-H), 7.67 (d, 1H, *J* = 15.5 Hz, 1-H), 7.57–7.54 (m, 3H, 5-Ar-2,6-H, 1-Ar-6-H), 7.48–7.46 (m, 3H, 5′-Ar-3, 4, 5-H), 7.03 (d, 1H, *J* = 16.0 Hz, 2-H), 7.00 (d, 1H, *J* = 15.0 Hz, 4-H), 6.95 (d, 2H, *J* = 9.0 Hz, 5-Ar-3,5-H), 6.52 (dd, 1H, *J* = 8.5, 2.0 Hz, 1-Ar-5-H), 6.46 (d, 1H, *J* = 2.0 Hz, 1-Ar-3-H), 4.45 (t, 2H, *J* = 6.0 Hz, -CH_2O-), 3.90 (s, 3H, OCH_3), 3.85 (s, 3H, OCH_3), 3.79 (t, 2H, *J* = 6.0 Hz, -CH_2S-); ^{13}C NMR (125 MHz, $CDCl_3$): δ 189.53, 168.80, 164.53, 163.04, 160.23, 160.13, 142.11, 138.49, 131.27, 130.52, 130.16, 130.16, 129.85, 129.33, 129.33, 128.42, 127.82, 127.82, 124.43, 123.75, 117.10, 115.09, 115.09, 105.49, 98.51, 66.31, 55.64, 55.60, 32.72. Anal. Calcd for $C_{29}H_{26}N_2O_4S_2$: C, 65.64; H, 4.94; N, 5.28; Found: C, 65.72; H, 5.04; N, 5.15. ESI-HRMS (m/z), calcd for $C_{29}H_{26}O_4N_2NaS_2$ [M+Na]$^+$ 553.12262, found 553.12231.

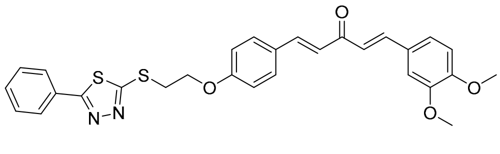

Data for *(1E,4E)-1-(3,4-dimethoxyphenyl)-5-(4-(2-((5-phenyl-1,3,4-thiadiazol-2-yl)thio)ethoxy) phenyl)penta-1,4-dien-3-one* (**4f**): Faint yellow powder; m.p. 146–147 °C; yield 87%; IR (KBr, cm^{-1}): *ν* 3439 (=CHCOCH=), 2933–2851 (CH), 1629 (C=O), 1601 (C=N), 1598–1451 (C=C and benzene), 1188 (–C–O–C); ^1H NMR (500 MHz, $CDCl_3$): *δ* 7.89–7.87 (m, 2H, 5′-Ar-2, 6-H), 7.69 (d, 1H, *J* = 16.0 Hz 5-H), 7.67 (d, 1H, *J* = 16.0 Hz, 1-H), 7.56 (d, 2H, *J* = 8.5 Hz, 5-Ar-2,6-H), 7.49–7.46 (m, 3H, 5′-Ar-3,4,5-H), 7.19 (dd, 1H, *J* = 9.0, 2.0 Hz, 1-Ar-6-H), 7.13 (d, 1H, *J* = 2.0 Hz, 1-Ar-2-H), 6.98 (d, 1H, *J* = 16.0 Hz, 2-H), 6.96 (d, 2H, *J* = 8.5 Hz, 5-Ar-3,5-H), 6.92 (d, 1H, *J* = 15.5 Hz, 4-H), 6.88 (d, 1H, *J* = 8.5 Hz, 1-Ar-5-H), 4.46 (t, 2H, *J* = 6.5 Hz, $-CH_2O-$), 3.94 (s, 3H, OCH_3), 3.92 (s, 3H, OCH_3), 3.78 (t, 2H, *J* = 6.0 Hz, $-CH_2S-$); ^{13}C NMR (125 MHz, $CDCl_3$): *δ* 189.53, 168.80, 164.53, 163.04, 160.23, 160.13, 142.11, 138.49, 131.27, 130.52, 130.16, 130.16, 129.85, 129.33, 129.33, 128.42, 127.82, 127.82, 124.43, 123.75, 117.10, 115.09, 115.09, 105.49, 98.51, 66.31, 55.64, 55.60, 32.72. Anal. Calcd for $C_{29}H_{26}N_2O_4S_2$: C, 65.64; H, 4.94; N, 5.28; Found: C, 65.83; H, 5.01; N, 5.06. ESI-HRMS (m/z), calcd for $C_{29}H_{26}O_4N_2NaS_2$ $[M+Na]^+$ 553.12262, found 553.12244.

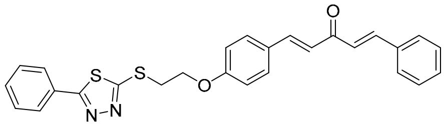

Data for *(1E,4E)-1-phenyl-5-(4-(2-((5-phenyl-1,3,4-thiadiazol-2-yl)thio)ethoxy)phenyl)penta-1,4-dien-3-one* (**4g**): Faint yellow powder; m.p. 148–149 °C; yield 85%; IR (KBr, cm^{-1}): *ν* 3438 (=CHCOCH=), 2934–2850 (CH), 1631 (C=O), 1608 (C=N), 1599–1444 (C=C and benzene), 1189 (–C–O–C); ^1H NMR (500 MHz, $CDCl_3$): *δ* 8.00 (d, 2H, *J* = 8.0 Hz, 5′-Ar-2, 6-H), 7.72 (d, 1H, *J* = 16.0 Hz,, 1-H), 7.69 (d, 1H, *J* = 16.0 Hz, 5-H), 7.61 (d, 2H, *J* = 6.0 Hz, 1-Ar-2, 6-H), 7.57 (d, 2H, *J* = 9.0 Hz, 5-Ar-2, 6-H), 7.54–7.48 (m, 3H, 5′-Ar-3, 4, 5-H), 7.43–7.41 (m, 3H, 1-Ar-3, 4, 5-H), 7.07 (d, 1H, *J* = 16.0 Hz, 4-H), 6.97 (d, 1H, *J* = 16.0 Hz, 2-H), 6.96 (d, 2H, *J* = 8.6 Hz, 5-Ar-3, 5-H), 4.46 (t, 2H, *J* = 6.3 Hz, $-CH_2O-$), 3.71 (t, 2H, *J* = 6.3 Hz, $-CH_2S-$); ^{13}C NMR (125 MHz, $CDCl_3$): *δ* 188.72, 165.91, 163.67, 160.00, 142.80, 142.80, 134.71, 131.66, 130.27, 130.08, 130.08, 128.95, 128.95, 128.81, 128.81, 128.21, 128.21, 127.93, 126.54, 126.54, 125.40, 123.41, 123.28, 114.88, 114.88, 65.81, 31.38. Anal. Calcd for $C_{27}H_{22}N_2O_2S_2$: C, 68.91; H, 4.71; N, 5.95; Found: C, 68.74; H, 4.85; N, 5.81. ESI-HRMS (m/z), calcd for $C_{27}H_{22}O_2N_2NaS_2$ $[M+Na]^+$ 493.10149, found 493.10080.

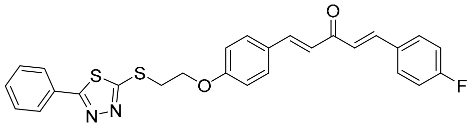

Data for *(1E,4E)-1-(4-fluorophenyl)-5-(4-(2-((5-phenyl-1,3,4-thiadiazol-2-yl)thio)ethoxy)phenyl) penta-1,4-dien-3-one* (**4h**): Faint yellow powder; m.p. 125–126 °C; yield 85%; IR (KBr, cm^{-1}): *ν* 3438 (=CHCOCH=), 2936–2851 (CH), 1633 (C=O), 1601 (C=N), 1598–1458 (C=C and benzene), 1242 (C–F), 1188(–C–O–C); ^1H NMR (500 MHz, $CDCl_3$): *δ* 7.89–7.87 (m, 2H, 5′-Ar-2, 6-H), 7.70 (d, 1H, *J* = 16.0 Hz, 5-H), 7.68 (d, 1H, *J* = 16.0 Hz, 1-H), 7.61–7.55 (m, 4H, 1,5-Ar-2, 6-H), 7.48–7.47 (m, 3H, 5′-Ar-3, 4, 5-H), 7.11–7.07 (m, 2H, 1-Ar-3, 5-H), 6.98 (d, 1H, *J* = 13.5 Hz, 2-H), 6.96 (d, 2H, *J* = 6.0 Hz, 5-Ar-3, 5-H), 6.93 (d, 1H, *J* = 16.0 Hz, 4-H), 4.46 (t, 2H, *J* = 5.5 Hz, $-CH_2O-$), 3.78 (t, 2H, *J* = 6.0 Hz, $-CH_2S-$); ^{13}C NMR (125 MHz, $CDCl_3$): *δ* 188.70, 168.81, 164.47,

163.06, 160.45, 143.17, 141.63, 131.27, 130.30, 130.30, 130.26, 129.83, 129.33, 127.82, 127.82, 125.36, 123.63, 116.27, 116.10, 115.20, 115.20, 66.38, 32.70. Anal. Calcd for $C_{27}H_{21}FN_2O_2S_2$: C, 66.37; H, 4.33; N, 5.73; Found: C, 66.24; H, 4.44; N, 5.69. ESI-HRMS (m/z), calcd for $C_{27}H_{21}O_2N_2FNaS_2$ $[M+Na]^+$ 511.09207, found 511.09146.

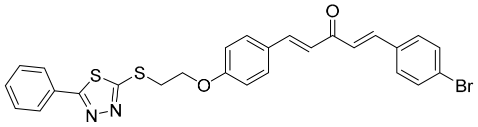

Data for *(1E,4E)-1-(4-bromophenyl)-5-(4-(2-((5-phenyl-1,3,4-thiadiazol-2-yl)thio)ethoxy)phenyl) penta-1,4-dien-3-one* (**4i**). Faint yellow powder; m.p. 148–149 ˚C; yield 89%; IR (KBr, cm^{-1}): *ν* 3438 (=CHCOCH=), 2928–2834 (CH), 1652 (C=O), 1610 (C=N), 1597–1453 (C=C and benzene), 1227 (C–Br), 1183 (–C–O–C); ^1H NMR (500 MHz, $CDCl_3$): *δ* 8.02–8.00 (m, 2H, 5′-Ar-2, 6-H), 7.70 (d, 1H, *J* = 16.0 Hz 1-H), 7.65 (d, 1H, *J* = 16.0 Hz, 5-H), 7.56–7.47 (m, 9H, 1-Ar-2, 6-H, 5-Ar-2, 6-H, 5′-Ar-3, 4, 5-H, 1-Ar-3, 5-H), 7.05 (d, 1H, *J* = 16.0 Hz, 4-H), 6.97 (d, 2H, *J* = 8.6 Hz, 5-Ar-3, 5-H), 6.94 (d, 1H, *J* = 16.0 Hz, 2-H), 4.47 (t, 2H, *J* = 6.3 Hz, $-CH_2O-$), 3.71 (t, 2H, *J* = 6.3 Hz, $-CH_2S-$); ^{13}C NMR (125 MHz, $CDCl_3$): *δ* 188.68, 166.20, 163.94, 160.37, 143.37, 141.62, 133.93, 132.32, 132.32, 131.93, 130.40, 130.40, 129.84, 129.84, 129.23, 129.23, 128.09, 126.80, 126.80, 126.09, 124.75, 123.64, 123.56, 115.19, 115.19, 66.08, 31.64. Anal. Calcd for $C_{27}H_{21}BrN_2O_2S_2$: C, 59.02; H, 3.85; N, 5.10; Found: C, 60.10; H, 3.80; N, 5.20. ESI-HRMS (m/z), calcd for $C_{27}H_{21}O_2N_2BrNaS_2$ $[M+Na]^+$ 571.01200, found 571.01178.

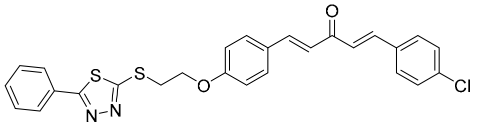

Data for *(1E,4E)-1-(4-chlorophenyl)-5-(4-(2-((5-phenyl-1,3,4-thiadiazol-2-yl)thio)ethoxy)phenyl) penta-1,4-dien-3-one* (**4j**). Faint yellow powder; m.p. 159–160 ˚C; yield 95%; IR (KBr, cm^{-1}): *ν* 3436 (=CHCOCH=), 2939–2853 (CH), 1629 (C=O), 1608 (C=N), 1598–1456 (C=C and benzene), 1239 (C–Cl), 1185 (–C–O–C); ^1H NMR (500 MHz, $CDCl_3$): *δ* 7.89–7.87 (m, 2H, 5′-Ar-2, 6-H), 7.70 (d, 1H, *J* = 16.0 Hz, 5-H), 7.68 (d, 1H, *J* = 16.0 Hz, 1-H), 7.56 (d, 2H, *J* = 9.0 Hz, 5-Ar-2, 6-H), 7.53 (d, 2H, *J* = 8.5 Hz, 1-Ar-2, 6-H), 7.48–7.45 (m, 3H, 5′-Ar-3, 4, 5-H), 7.37 (d, 2H, *J* = 8.5 Hz, 1-Ar-3, 5-H), 7.02 (d, 1H, *J* = 16.0 Hz, 2-H), 6.97 (d, 2H, *J* = 8.5 Hz, 5-Ar-3, 5-H), 6.93 (d, 1H, *J* = 15.5 Hz, 4-H), 4.47 (t, 2H, *J* = 6.0 Hz, $-CH_2O-$), 3.79 (t, 2H, *J* = 6.0 Hz, $-CH_2S-$); ^{13}C NMR (125 MHz, $CDCl_3$): *δ* 188.64, 168.82, 164.46, 160.50, 143.35, 141.46, 136.33, 133.52, 131.27, 130.33, 130.33, 129.84, 129.57, 129.57, 129.31, 129.31, 127.99, 127.82, 127.82, 126.03, 123.59, 115.21, 115.21, 66.38, 32.68. Anal. Calcd for $C_{27}H_{21}ClN_2O_2S_2$: C, 64.21; H, 4.19; N, 5.55; Found: C, 64.19; H, 4.23; N, 5.48. ESI-HRMS (m/z), calcd for $C_{27}H_{21}O_2N_2ClNaS_2$ $[M+Na]^+$ 527.06252, found 527.06195.

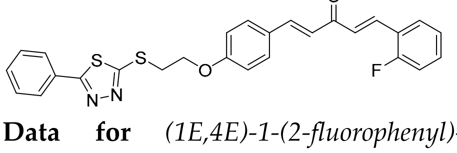

Data for *(1E,4E)-1-(2-fluorophenyl)-5-(4-(2-((5-phenyl-1,3,4-thiadiazol-2-yl)thio)ethoxy)phenyl) penta-1,4-dien-3-one* (**4k**). Faint yellow powder; m.p. 138–139 ˚C; yield 84%; IR (KBr, cm^{-1}): *ν* 3439 (=CHCOCH=), 2939–2855 (CH), 1629 (C=O), 1607 (C=N), 1598–1453 (C=C and benzene), 1245 (C–F), 1186 (–C–O–C); ^1H NMR (500 MHz, $CDCl_3$): *δ* 7.90–7.89 (m, 2H, 5′-Ar-2, 6-H), 7.83 (d, 1H, *J* = 16.0 Hz, 5-H), 7.71 (d, 1H, *J* = 16.0 Hz, 1-H), 7.60 (d, 1H, *J* = 9.0 Hz, 1-Ar-6-H), 7.57 (d, 2H, *J* = 9.0 Hz, 5-Ar-2,6-H), 7.49–7.47 (m, 3H, 5′-Ar-3, 4, 5-H), 7.38–7.37 (m, 1H, 1-Ar-4-H),

7.27–7.13 (m, 3H, 2-H, 4-H, 1-Ar-3-H), 6.99–6.96 (m, 3H, 1-Ar-5-H, 5-Ar-3,5-H,), 4.47 (t, 2H, *J* = 6.0 Hz, -CH_2O-), 3.79 (t, 2H, *J* = 6.0 Hz, -CH_2S-); ^{13}C NMR (125 MHz, $CDCl_3$): *δ* 188.94, 168.81, 164.47, 160.47, 143.37, 135.50, 131.80, 131.73, 131.27, 130.35, 130.35, 129.86, 129.47, 129.33, 129.33, 128.06, 127.82, 127.82, 124.59, 123.52, 123.07, 116.41, 116.24, 115.19, 115.19, 66.38, 32.72. Anal. Calcd for $C_{27}H_{21}FN_2O_2S_2$: C, 66.37; H, 4.33; N, 5.73; Found: C, 69.27; H, 4.26; N, 5.71. ESI-HRMS (m/z), calcd for $C_{27}H_{21}O_2N_2FNaS_2$ $[M+Na]^+$ 511.09207, found 511.09152.

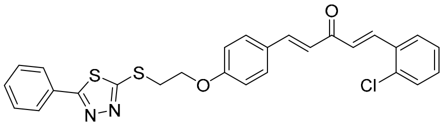

Data for *(1E,4E)-1-(2-chlorophenyl)-5-(4-(2-((5-phenyl-1,3,4-thiadiazol-2-yl)thio)ethoxy)phenyl) penta-1,4-dien-3-one* (**4l**). Faint yellow powder; m.p. 150–151 °C; yield 81%; IR (KBr, cm^{-1}): *ν* 3437 (=CHCOCH=), 2939–2856 (CH), 1629 (C=O), 1608 (C=N), 1598–1453 (C=C and benzene), 1239 (C–Cl), 1185 (–C–O–C); ^{1}H NMR (500 MHz, $CDCl_3$): *δ* 8.09 (d, 1H, *J* = 16.0 Hz, 1-H), 7.89–7.87 (m, 2H, 5′-Ar-2, 6-H), 7.72–7.69 (m, 2H, 1, 5-H), 7.57 (d, 2H, *J* = 7.0 Hz, 5-Ar-2, 6-H), 7.50–7.47 (m, 3H, 5′-Ar-3, 4, 5-H), 7.43 (d, 1H, *J* = 6.0 Hz, 1-Ar-4-H), 7.32–7.25 (m, 2H, 4-H, 1-Ar-3-H), 7.01 (d, 1H, *J* = 14.0 Hz 2-H), 7.00–6.97 (m, 3H, 1-Ar-5-H, 5-Ar-3,5-H), 4.47 (t, 2H, *J* = 6.0 Hz, -CH_2O-), 3.79 (t, 2H, *J* = 6.0 Hz, -CH_2S-); ^{13}C NMR (125 MHz, $CDCl_3$): *δ* 188.81, 168.81, 164.46, 160.50, 143.51, 138.62, 135.42, 133.33, 131.27, 131.11, 130.35, 130.35, 129.85, 129.33, 129.33, 128.38, 128.02, 127.82, 127.82, 127.76, 127.17, 123.10, 115.20, 115.20, 66.38, 32.71. Anal. Calcd for $C_{27}H_{21}ClN_2O_2S_2$: C, 64.21; H, 4.19; N, 5.55; Found: C, 64.25; H, 4.24; N, 5.61. ESI-HRMS (m/z), calcd for $C_{27}H_{21}O_2N_2ClNaS_2$ $[M+Na]^+$ 527.06252, found 527.06238.

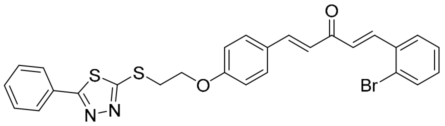

Data for *(1E,4E)-1-(2-bromophenyl)-5-(4-(2-((5-phenyl-1,3,4-thiadiazol-2-yl)thio)ethoxy)phenyl) penta-1,4-dien-3-one* (**4m**). Faint yellow powder; m.p. 153–154 °C; yield 83%; IR (KBr, cm^{-1}): *ν* 3435 (=CHCOCH=), 2939–2858 (CH), 1629 (C=O), 1607 (C=N), 1598–1453 (C=C and benzene), 1235 (C–Cl), 1181 (–C–O–C); ^{1}H NMR (500 MHz, $CDCl_3$): *δ* 8.04 (d, 1H, *J* = 16.0 Hz, 1-H), 7.88–7.87 (m, 2H, 5′-Ar-2, 6-H), 7.72–7.68 (m, 2H, 1, 5-H), 7.62 (d, 1H, *J* = 8.0 Hz, 1-Ar-3-H), 7.56 (d, 2H, *J* = 8.5 Hz, 5-Ar-2, 6-H), 7.48–7.45 (m, 3H, 5′-Ar-3, 4, 5-H), 7.34 (t, 1H, *J* = 7.5 Hz, 1-Ar-5-H), 7.26–7.21 (m, 1H, 1-Ar-4-H), 7.00–6.97 (m, 4H, 1-Ar-6-H, 5-Ar-3,5-H, 2-H), 4.46 (t, 2H, *J* = 6.0 Hz, -CH_2O-), 3.78 (t, 2H, *J* = 6.0 Hz, -CH_2S-); ^{13}C NMR (125 MHz, $CDCl_3$): *δ* 188.80, 168.81, 164.47, 160.50, 143.56, 141.21, 135.12, 133.59, 131.27, 131.27, 130.36, 130.36, 129.85, 129.33, 129.33, 128.58, 128.01, 127.90, 127.82, 127.82, 127.82, 125.87, 123.07, 115.21, 115.21, 66.39, 32.71. Anal. Calcd for $C_{27}H_{21}BrN_2O_2S_2$: C, 59.02; H, 3.85; N, 5.10; Found: C, 60.13; H, 3.78; N, 5.02. ESI-HRMS (m/z), calcd for $C_{27}H_{21}O_2N_2BrNaS_2$ $[M+Na]^+$ 571.01200, found 571.01154.

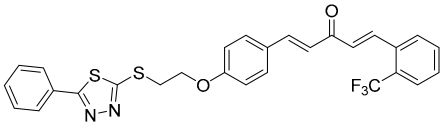

Data for *(1E,4E)-1-(4-(2-((5-phenyl-1,3,4-thiadiazol-2-yl)thio)ethoxy)phenyl)-5-(2-(trifluorometh yl)phenyl)penta-1,4-dien-3-one* (**4n**). Faint yellow powder; m.p. 134–135 °C; yield 95%; IR (KBr, cm^{-1}): *ν* 3458 (=CHCOCH=), 2939–2852 (CH), 1657 (C=O), 1615 (C=N), 1597–1455 (C=C and benzene), 1327 (C-F), 1172 (–C–O–C); ^{1}H NMR (500 MHz, $CDCl_3$): *δ* 8.05 (d, 1H, *J* = 16.0 Hz, 5-H), 8.00 (d, 2H, *J* = 7.0 Hz, 5′-Ar-2, 6-H), 7.79 (d, 1H, *J* = 8.0 Hz, 5-Ar-3-H), 7.73 (d, 1H, *J* = 7.5

Hz, 5-Ar-6-H), 7.69 (d, 1H, *J* = 16.0 Hz, 1-H), 7.59 (t, 1H, *J* = 7.0 Hz, 5-Ar-5-H), 7.56 (d, 2H, *J* = 9.5 Hz, 1-Ar-2, 6-H), 7.54–7.48 (m, 4H, 5′-Ar-3, 4, 5-H, 5-Ar-4-H), 6.99 (d, 1H, *J* =16.0 Hz, 2-H), 6.97 (d, 2H, *J* = 7.5 Hz, 1-Ar-3, 5-H), 6.96 (d, 1H, *J* = 16.0 Hz, 4-H), 4.47 (t, 2H, *J* = 6.0 Hz, -CH_2O-), 3.71 (t, 2H, *J* = 6.0 Hz, -CH_2S-); ^{13}C NMR (125 MHz, $CDCl_3$): δ 188.80, 166.23, 163.97, 160.46, 143.90, 138.30, 134.10, 132.29, 131.97, 130.48, 130.48, 129.95, 129.73, 129.26, 129.26, 129.26, 128.08, 128.08, 126.85, 126.85, 126.43, 123.62, 123.06, 115.24, 115.24, 66.14, 31.70. Anal. Calcd for $C_{28}H_{21}F_3N_2O_2S_2$: C, 62.44; H, 3.93; N, 5.20; Found: C, 62.33; H, 3.87; N, 5.14. ESI-HRMS (m/z), calcd for $C_{28}H_{21}O_2N_2F_3NaS_2$ $[M+Na]^+$ 561.08888, found 561.08838.

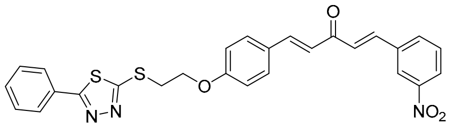

Data for *(1E,4E)-1-(3-nitrophenyl)-5-(4-(2-((5-phenyl-1,3,4-thiadiazol-2-yl)thio)ethoxy)phenyl) penta-1,4-dien-3-one* (**4o**). Faint yellow powder; m.p. 186–187 ˚C; yield 71%; IR (KBr, cm^{-1}): ν 3464 (=CHCOCH=), 2942–2856 (CH), 1659 (C=O), 1618 (C=N), 1599–1458 (C=C and benzene), 1178 (–C–O–C); ^1H NMR (500 MHz, $CDCl_3$): δ 8.47 (brs, 1H, 1-H), 8.23 (d, 2H, *J* = 7.5 Hz, 5′-Ar-2, 6-H), 7.88–7.87 (brs, 3H, 1-Ar-3-H, 5-Ar-2, 6-H), 7.72 (d, 2H, *J* = 16.0 Hz, 1, 5-H), 7.61–7.58 (m, 3H, 5′-Ar-3, 4, 5-H), 7.48 (brs, 4H, 1-Ar-2, 4, 5, 6-H), 7.19 (d, 1H, *J* = 15.5 Hz, 4-H), 6.98 (d, *J* = 8.5 Hz, 5-Ar-3,5-H), 6.94 (d, *J* = 16.0 Hz, 2-H), 4.46 (t, 2H, *J* = 6.0 Hz, -CH_2O-), 3.78 (t, 2H, *J* = 6.0 Hz, -CH_2S-); ^{13}C NMR (125 MHz, $CDCl_3$): δ 188.15, 168.87, 164.44, 160.71, 148.83, 144.08, 139.82, 136.85, 134.26, 131.28, 130.48, 130.48, 130.08, 129.84, 129.34, 129.34, 127.91, 127.82, 127.82, 124.57, 123.55, 122.39, 115.27, 115.27, 66.40, 32.66. Anal. Calcd for $C_{27}H_{21}N_3O_4S_2$: C, 62.90; H, 4.11; N, 8.15; Found: C, 62.86; H, 4.06; N, 8.21. ESI-HRMS (m/z), calcd for $C_{27}H_{21}O_4N_3NaS_2$ $[M+Na]^+$ 538.08657, found 538.08588.

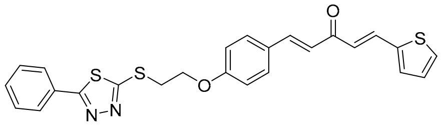

Data for *(1E,4E)-1-(4-(2-((5-phenyl-1,3,4-thiadiazol-2-yl)thio)ethoxy)phenyl)-5-(thiophen-2-yl) penta-1,4-dien-3-one* (**4p**): Faint yellow powder; m.p. 123–124 ˚C; yield 79%; IR (KBr, cm^{-1}): ν 3428 (=CHCOCH=), 2918–2845 (CH), 1623 (C=O), 1610 (C=N), 1585–1449 (C=C and benzene), 1181 (–C–O–C); ^1H NMR (500 MHz, $CDCl_3$): δ 7.88 (d, 2H, *J* = 7.0 Hz, 5′-Ar-2, 6-H), 7.84 (d, 1H, *J* = 16.0 Hz, 5-H), 7.68 (d, 1H, *J* = 16.0 Hz, 1-H), 7.56 (d, 2H, *J* = 8.5 Hz, 5-Ar-2, 6-H), 7.48–7.47 (m, 3H, 5′-Ar-3, 4, 5-H), 7.40 (d, 1H, *J* = 5.0 Hz, thiophen-5-H), 7.32 (d,1H, *J* = 3.5 Hz, thiophen-3-H), 7.08 (t, 1H, *J* = 3.5 Hz, thiophen-4-H), 6.96 (d, 2H, *J* = 8.5 Hz, 5-Ar-3, 5-H), 6.90 (d, 1H, *J* =16.0 Hz, 2-H), 6.86 (d, 1H, *J* = 16.0 Hz, 4-H), 4.46 (t, 2H, *J* = 6.0 Hz, -CH_2O-), 3.78 (t, 2H, *J* = 6.0 Hz, -CH_2S-); ^{13}C NMR (125 MHz, $CDCl_3$): δ 188.38, 168.81, 164.50, 160.40, 142.95, 140.51, 135.50, 131.81, 131.28, 130.30, 130.30, 129.83, 129.34, 129.34, 128.73, 128.40, 128.07, 127.82, 127.82, 124.50, 123.81, 115.16, 115.16, 66.34, 32.69. Anal. Calcd for $C_{25}H_{20}N_2O_2S_3$: C, 63.00; H, 4.23; N, 5.88; Found: C, 62.85; H, 4.16; N, 5.85. ESI-HRMS (m/z), calcd for $C_{25}H_{20}O_2N_2NaS_3$ $[M+Na]^+$ 499.05791, found 499.05756.

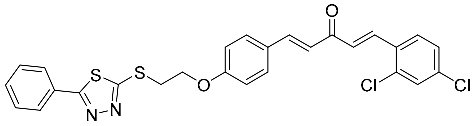

Data for *(1E,4E)-1-(2,4-dichlorophenyl)-5-(4-(2-((5-phenyl-1,3,4-thiadiazol-2-yl)thio)ethoxy)*

phenyl)penta-1,4-dien-3-one (**4q**). Faint yellow powder; m.p. 139–140 °C; yield 80%; IR (KBr, cm^{-1}): *ν* 3431 (=CHCOCH=), 2937–2859 (CH), 1635 (C=O), 1609 (C=N), 1598–1458 (C=C and benzene), 1247 (C–Cl), 1185 (–C–O–C); ^{1}H NMR (500 MHz, $CDCl_3$): *δ* 8.01 (d, 1H, *J* = 16.0 Hz 1-H), 7.92 (brs, 2H, 5′-Ar-2, 6-H), 7.69 (d, 1H, *J* = 15.0 Hz, 5-H), 7.63 (d, 1H, *J* = 8.5 Hz, 1-Ar-6-H), 7.56 (d, 2H, *J* = 8.5 Hz, 5-Ar-2, 6-H), 7.48–7.45 (m, 4H, 1-Ar-3-H, 5′-Ar-3, 4, 5-H), 7.29–7.25 (m, 2H, 4-H, 1-Ar-5-H), 7.01–6.94 (m, 3H, 5-Ar-3, 5-H, 2-H), 4.46 (t, 2H, *J* = 6.0 Hz, -CH_2O-), 3.78 (t, 2H, *J* = 6.0 Hz, -CH_2S-); ^{13}C NMR (125 MHz, $CDCl_3$): *δ* 188.49, 168.82, 164.45, 160.58, 143.75, 137.35, 135.98, 131.94, 131.27, 130.40, 130.40, 130.17, 129.84, 129.33, 129.33, 128.54, 128.48, 127.92, 127.82, 127.82, 127.66, 123.11, 115.23, 115.23, 66.39, 32.67. Anal. Calcd for $C_{27}H_{20}ClFN_2O_2S_2$: C, 60.11; H, 3.74; N, 5.19; Found: C, 60.25; H, 3.72; N, 5.33. ESI-HRMS (m/z), calcd for $C_{27}H_{20}O_2N_2Cl_2NaS_2$ $[M+Na]^+$ 561.02355, found 561.02332.

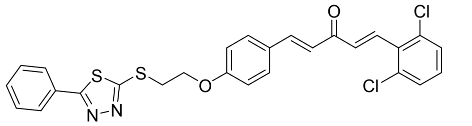

Data for *(1E,4E)-1-(2,6-dichlorophenyl)-5-(4-(2-((5-phenyl-1,3,4-thiadiazol-2-yl)thio)ethoxy) phenyl)penta-1,4-dien-3-one* (**4r**). Faint yellow powder; m.p. 145–147 °C; yield 83%; IR (KBr, cm^{-1}): *ν* 3435 (=CHCOCH=), 2924–2836 (CH), 1648 (C=O), 1609 (C=N), 1585–1451 (C=C and benzene), 1245 (C–Cl), 1180 (–C–O–C); ^{1}H NMR (500 MHz, $CDCl_3$): *δ* 7.87 (brs, 2H, 5′-Ar-2, 6-H), 7.74 (d, 1H, *J* = 16.5 Hz, 5-H), 7.69 (d, 1H, *J* = 16.0 Hz, 1-H), 7.55 (d, 1H, *J* = 6.5 Hz, 1-Ar-6-H), 7.48 (brs, 2H, 5-Ar-2, 6-H), 7.36 (d, 2H, *J* = 8.0 Hz, 5′-Ar-3, 5-H), 7.29 (d, 1H, *J* = 8.0 Hz, 1-Ar-2-H), 7.23–7.14 (m, 1H, 5′-Ar-4-H), 6.96 (d, 2H, *J* = 8.5 Hz, 5-Ar-3, 5-H), 6.9 (t, 1H, *J* = 6.0 Hz, 1-Ar-4-H), 6.68 (d, 1H, *J* = 16.0 Hz, 4-H), 6.54 (d, 1H, *J* = 16.5 Hz, 2-H), 4.43 (t, 2H, *J* = 6.0 Hz, -CH_2O-), 3.76 (t, 2H, *J* = 6.0 Hz, -CH_2S-); ^{13}C NMR (125 MHz, $CDCl_3$): *δ* 190.03, 188.81, 168.82, 164.47, 160.57, 143.94, 143.59, 136.21, 135.23, 134.92, 133.75, 133.39, 132.74, 131.77, 131.27, 130.42, 130.19, 129.81129.33, 129.33, 128.91, 127.93, 127.82, 127.82, 123.94, 123.72, 115.21, 115.09, 66.38, 32.70. Anal. Calcd for $C_{27}H_{20}Cl_2N_2O_2S_2$: C, 62.11; H, 3.74; N, 5.19; Found: C, 62.22; H, 3.84; N, 5.20. ESI-HRMS (m/z), calcd for $C_{27}H_{20}O_2N_2Cl_2NaS_2$ $[M+Na]^+$ 561.02355, found 561.02332.

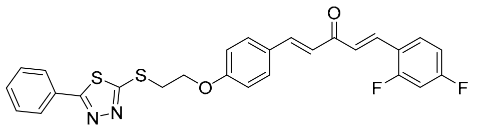

Data for *(1E,4E)-1-(2,4-difluorophenyl)-5-(4-(2-((5-phenyl-1,3,4-thiadiazol-2-yl)thio)ethoxy) phenyl)penta-1,4-dien-3-one* (**4s**). Faint yellow powder; m.p. 143–144 °C; yield 81%; IR (KBr, cm^{-1}): *ν* 3438 (=CHCOCH=), 2928–2835 (CH), 1649 (C=O), 1610 (C=N), 1588–1456 (C=C and benzene), 1238 (C–F), 1187 (–C–O–C); ^{1}H NMR (500 MHz, $CDCl_3$): *δ* 7.88 (d, 2H, *J* = 7.0 Hz, 5′-Ar-2, 6-H), 7.75 (d, 1H, *J* = 16.0 Hz, 1-H), 7.69 (d, 1H, *J* = 15.5 Hz, 5-H), 7.60 (d, 1H, *J* = 6.0 Hz, 1-Ar-6-H), 7.57 (d, 2H, *J* = 8.5 Hz, 5-Ar-2, 6-H), 7.48–7.45 (m, 3H, 5′-Ar-3, 4, 5-H), 7.10 (d, 1H, *J* =16.0 Hz, 4-H), 6.96 (d, 2H, *J* = 9.0 Hz, 5-Ar-3, 5-H), 6.94 (d, 1H, *J* = 16.5 Hz, 2-H), 6.93–6.86 (m, 2H, 1-Ar-3, 5-H), 4.47 (t, 2H, *J* = 6.0 Hz, -CH_2O-), 3.79 (t, 2H, *J* = 6.0 Hz, -CH_2S-); ^{13}C NMR (125 MHz, $CDCl_3$): *δ* 188.74, 168.81, 164.47, 162.80, 160.51, 143.49, 134.51, 131.28, 130.45, 130.38, 130.38, 129.83, 129.34, 129.34, 127.96, 127.82, 127.82, 127.56, 123.48, 115.18, 115.18, 66.35, 32.66. Anal. Calcd for $C_{27}H_{20}F_2N_2O_2S_2$: C, 64.01; H, 3.98; N, 5.53; Found: C, 64.11; H, 3.85; N, 5.40. ESI-HRMS (m/z), calcd for $C_{27}H_{20}O_2N_2F_2NaS_2$ $[M+Na]^+$ 529.08265, found 529.08221.

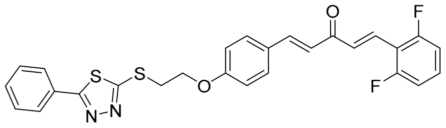

Data for *(1E,4E)-1-(2,6-difluorophenyl)-5-(4-(2-((5-phenyl-1,3,4-thiadiazol-2-yl)thio)ethoxy) phenyl)penta-1,4-dien-3-one* (**4t**). Faint yellow powder; m.p. 142–143 °C; yield 78%; IR (KBr, cm^{-1}): ν 3438 (=CHCOCH=), 2937–2853 (CH), 1625 (C=O), 1606 (C=N), 1599–1457 (C=C and benzene), 1242 (C–F), 1186 (–C–O–C); ^{1}H NMR (500 MHz, $CDCl_3$): δ 7.89 (brs, 2H, 5′-Ar-2, 6-H), 7.79 (d, 1H, J = 16.5 Hz, 5-H), 7.69 (d, 1H, J = 15.5 Hz, 1-H), 7.56 (d, 2H, J = 9.0 Hz, 5-Ar-2,6-H), 7.48 (brs, 3H, 5′-Ar-3, 4, 5-H), 7.37 (d, 1H, J = 16.0 Hz, 1-Ar-4-H), 7.32–7.25 (m, 1H, 2-H, 1-Ar-4-H), 6.97–6.91 (m, 5H, 1-Ar-3,5-H, 5-Ar-3,5-H, 2-H), 4.46 (t, 2H, J = 6.0 Hz, -CH_2O-), 3.78 (t, 2H, J = 6.0 Hz, -CH_2S-); ^{13}C NMR (125 MHz, $CDCl_3$): δ 189.04, 168.80, 164.46, 163.01, 160.98, 160.51, 143.57, 131.26, 131.10, 130.86, 130.39, 130.39, 129.86, 129.32,129.32, 128.89, 128.01, 127.82, 127.82, 123.98, 115.19, 115.19, 113.10, 112.07, 111.86, 66.38, 32.72. Anal. Calcd for $C_{27}H_{20}F_2N_2O_2S_2$: C, 64.01; H, 3.98; N, 5.53; Found: C, 64.08; H, 3.92; N, 5.57. ESI-HRMS (m/z), calcd for $C_{27}H_{20}O_2N_2F_2NaS_2$ $[M+Na]^+$ 529.08265, found 529.08215.

2. Spectrogram of title compounds 4a-4t

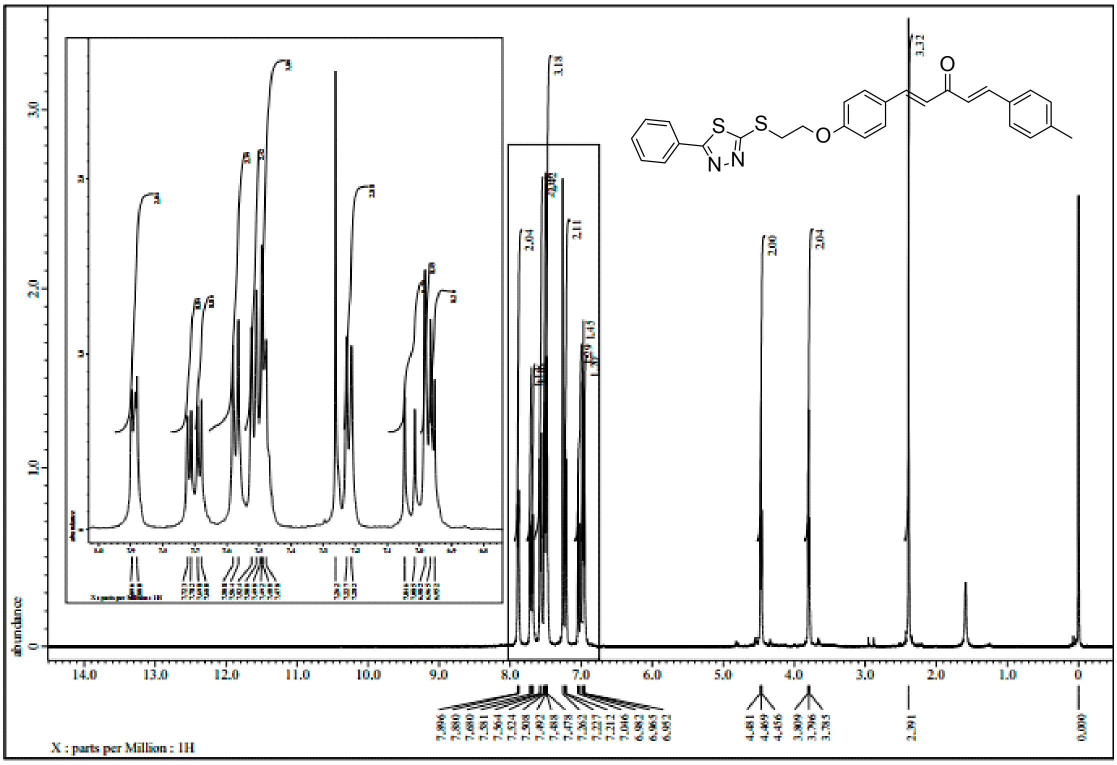

^{1}H NMR of compound **4a**

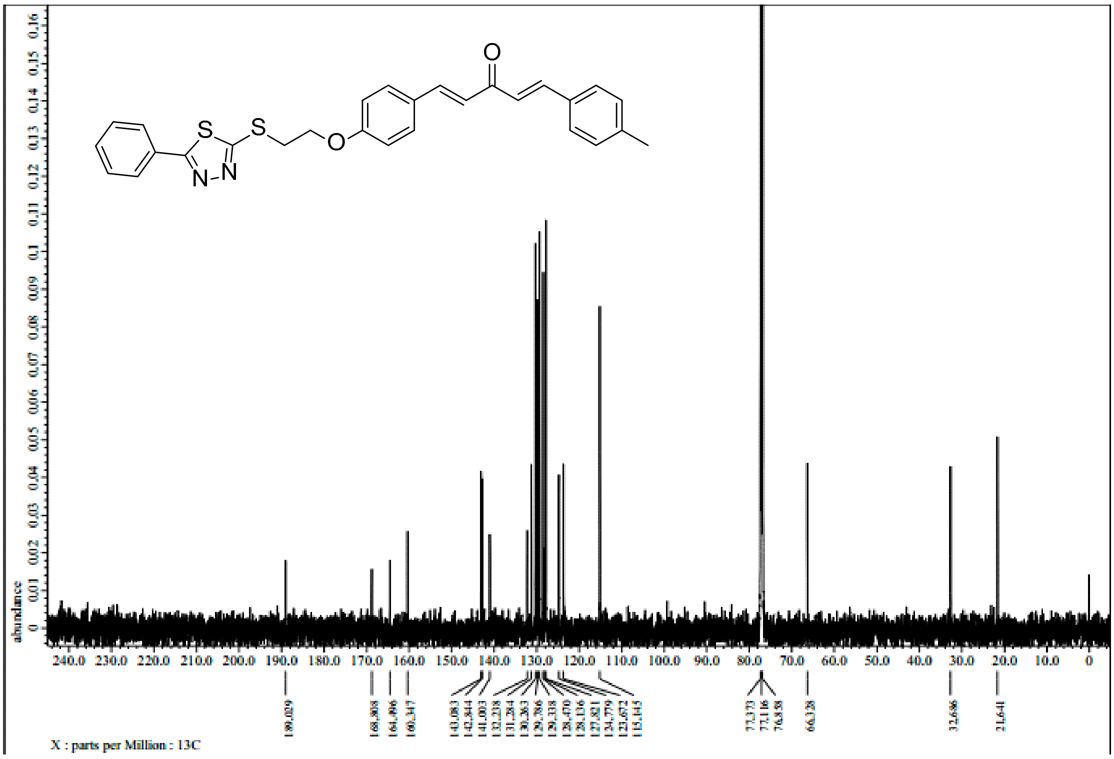

^{13}C NMR of compound **4a**

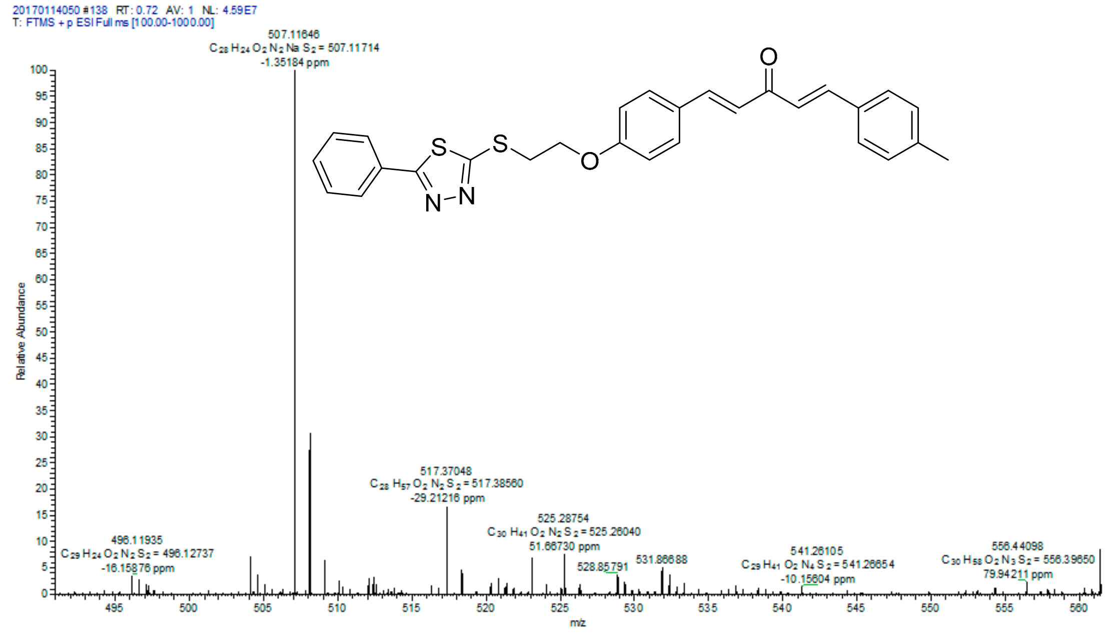

HRMS of compound **4a**

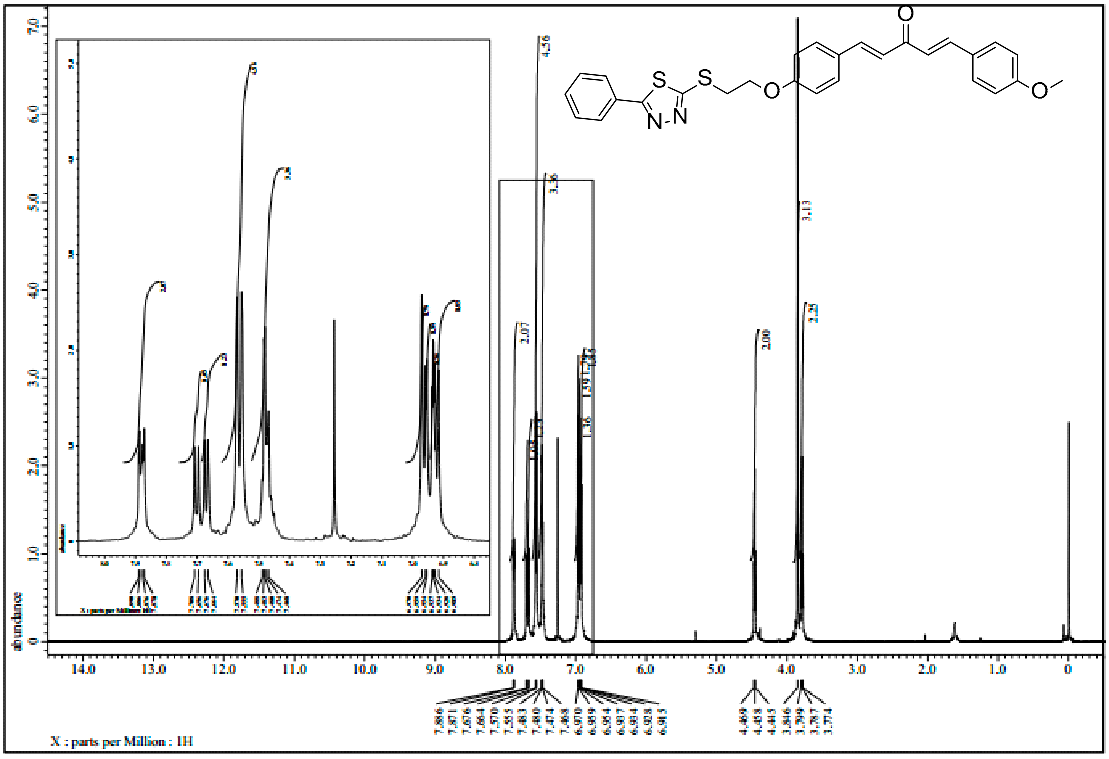

^{1}H NMR of compound **4b**

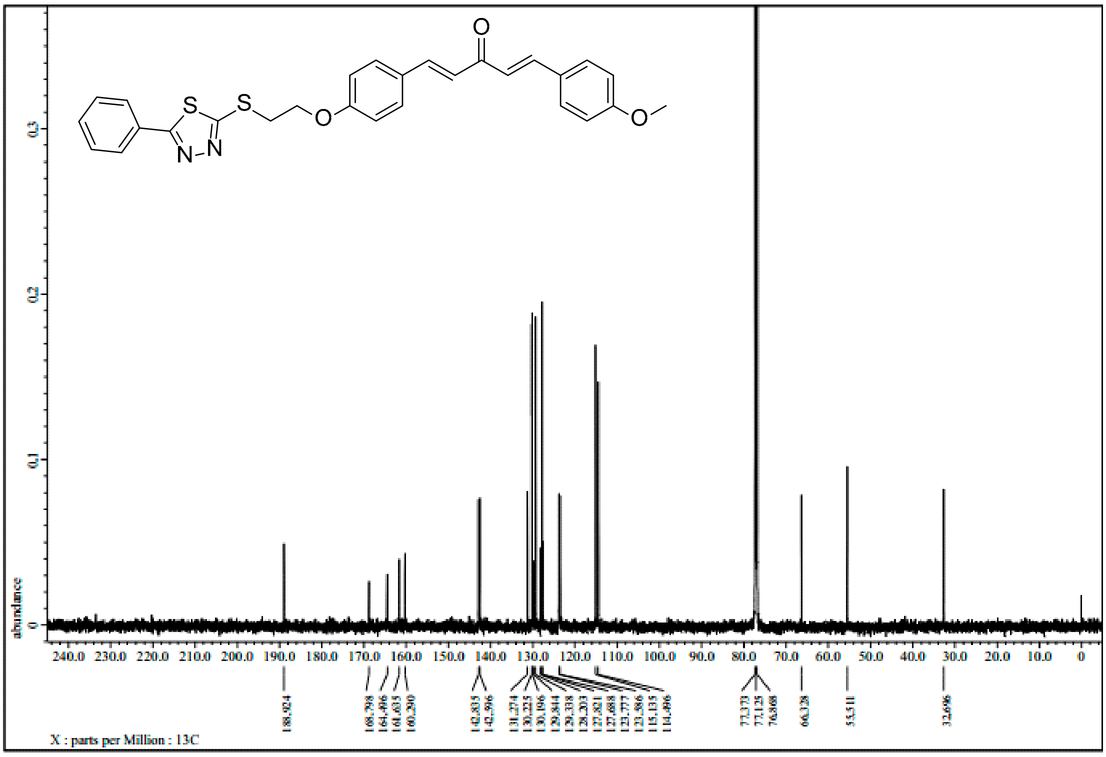

^{13}C NMR of compound **4b**

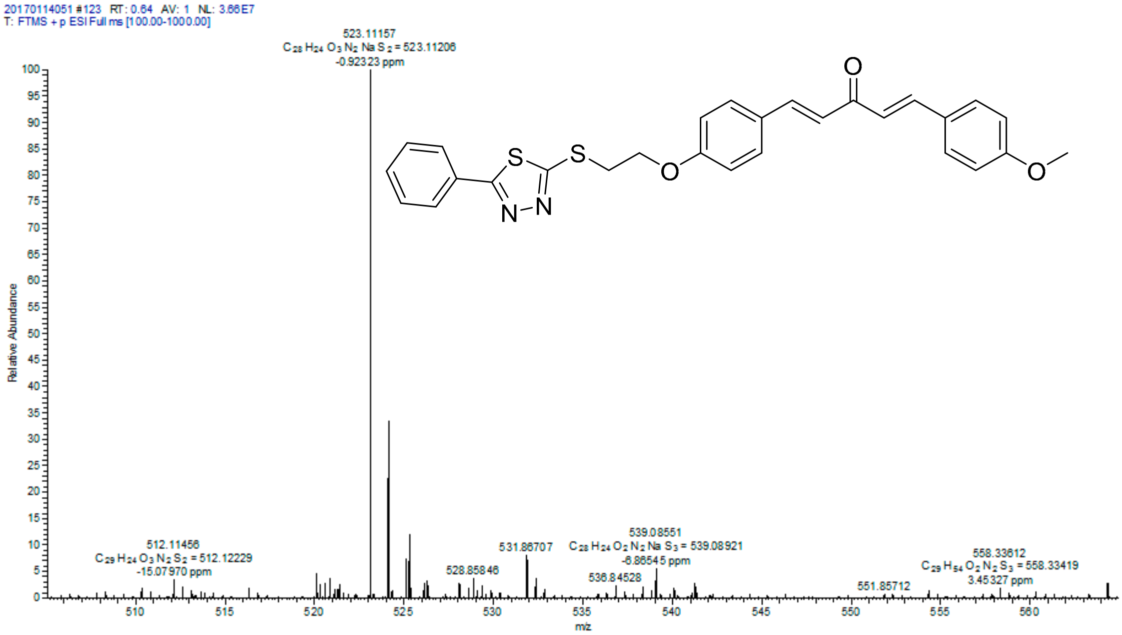

HRMS of compound **4b**

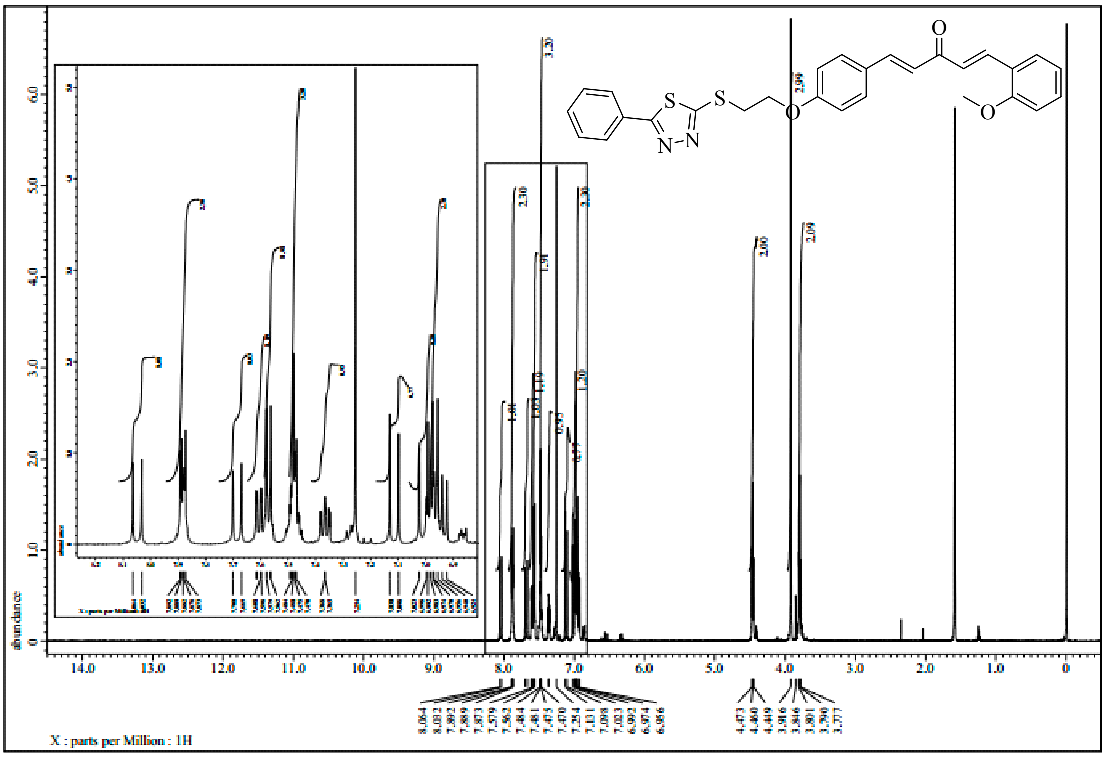

^{1}H NMR of compound **4c**

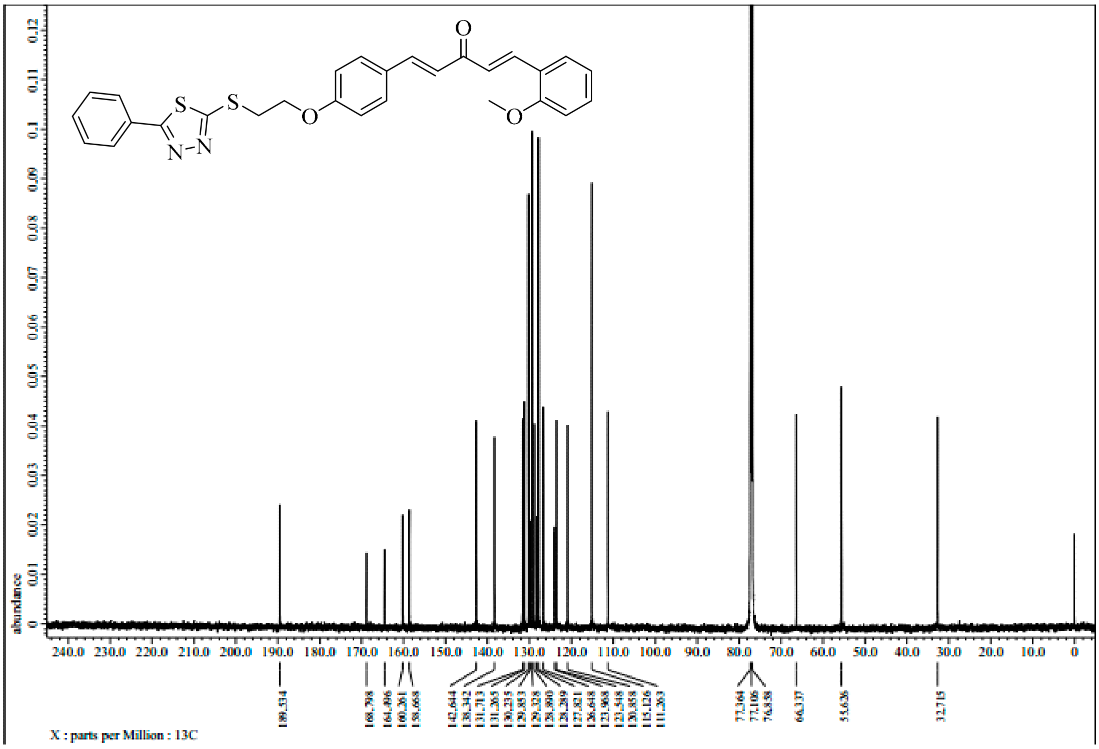

^{13}C NMR of compound **4c**

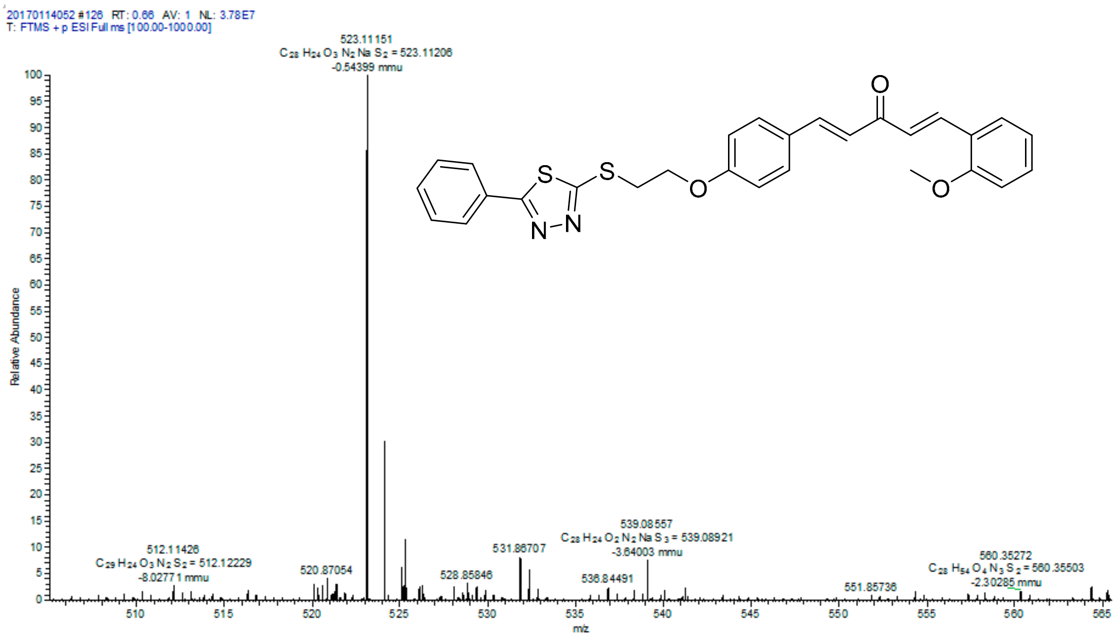

HRMS of compound **4c**

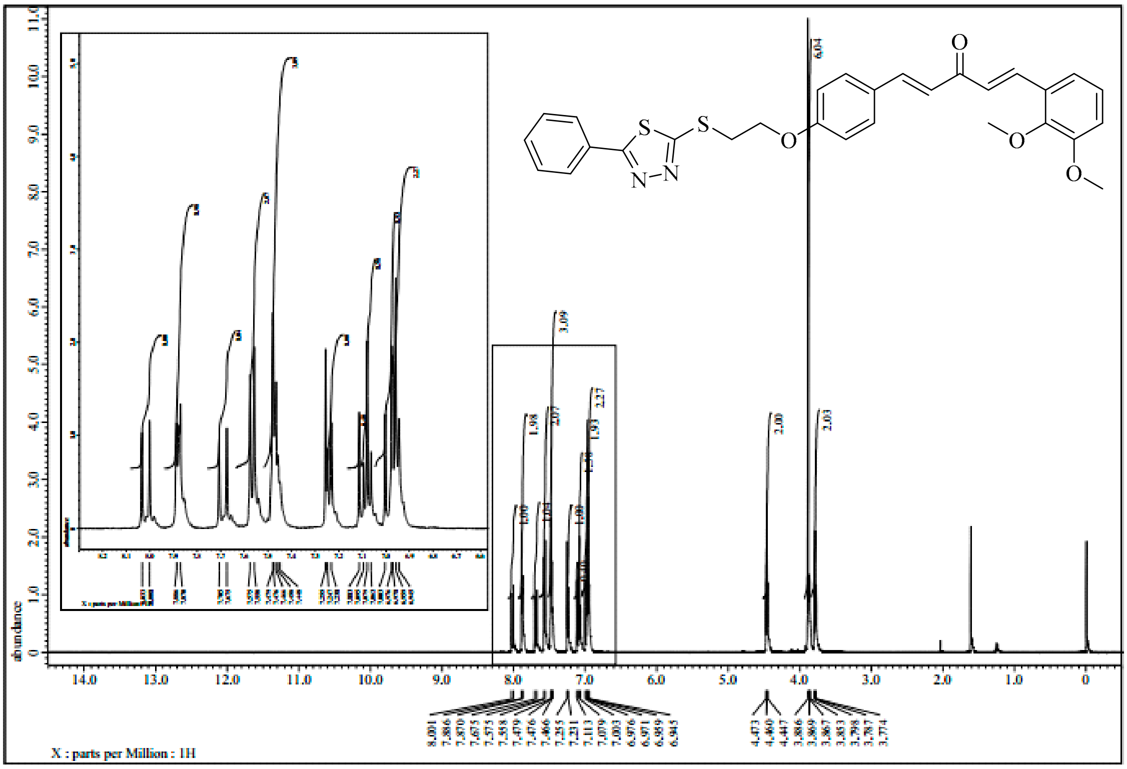

^{1}H NMR of compound **4d**

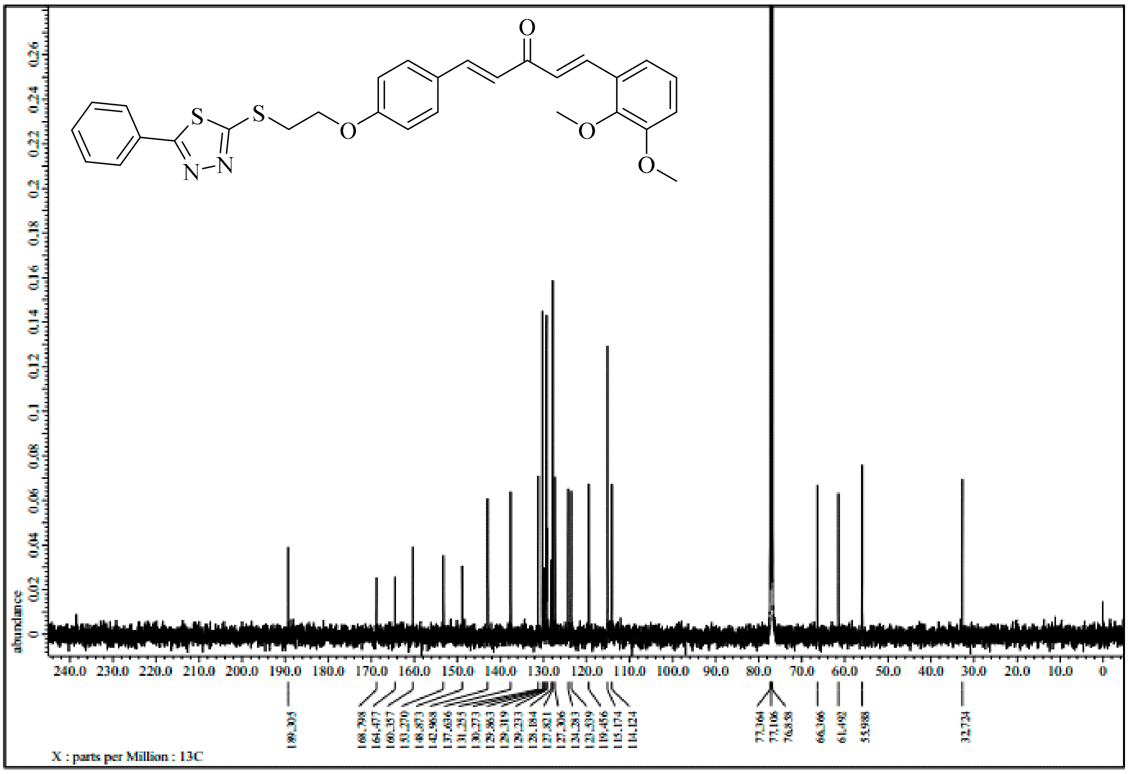

^{13}C NMR of compound **4d**

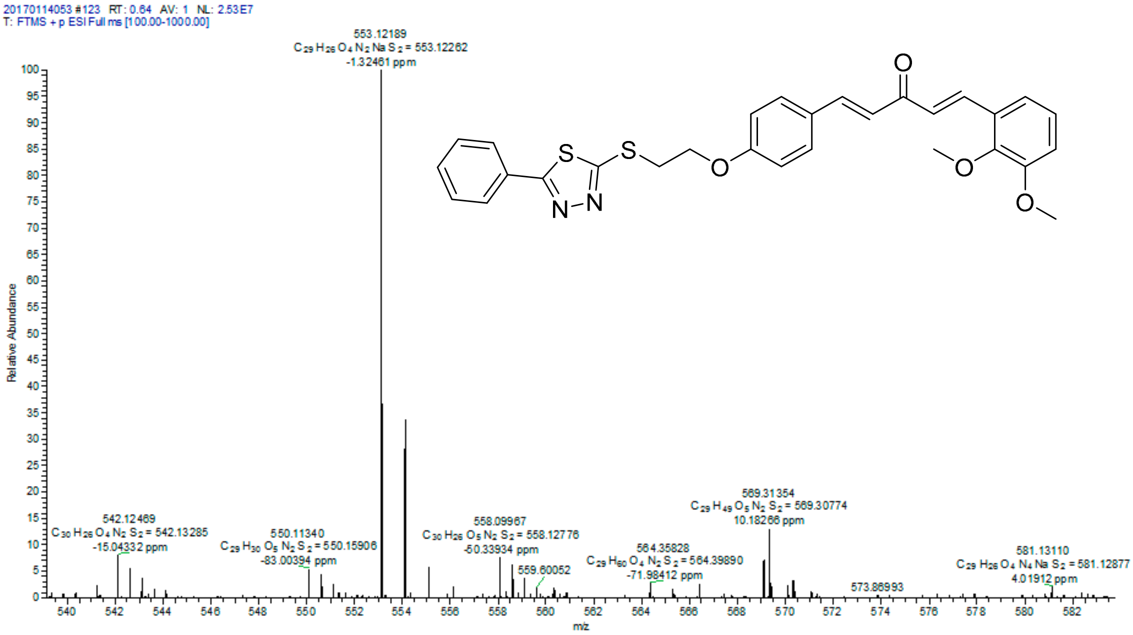

HRMS of compound **4d**

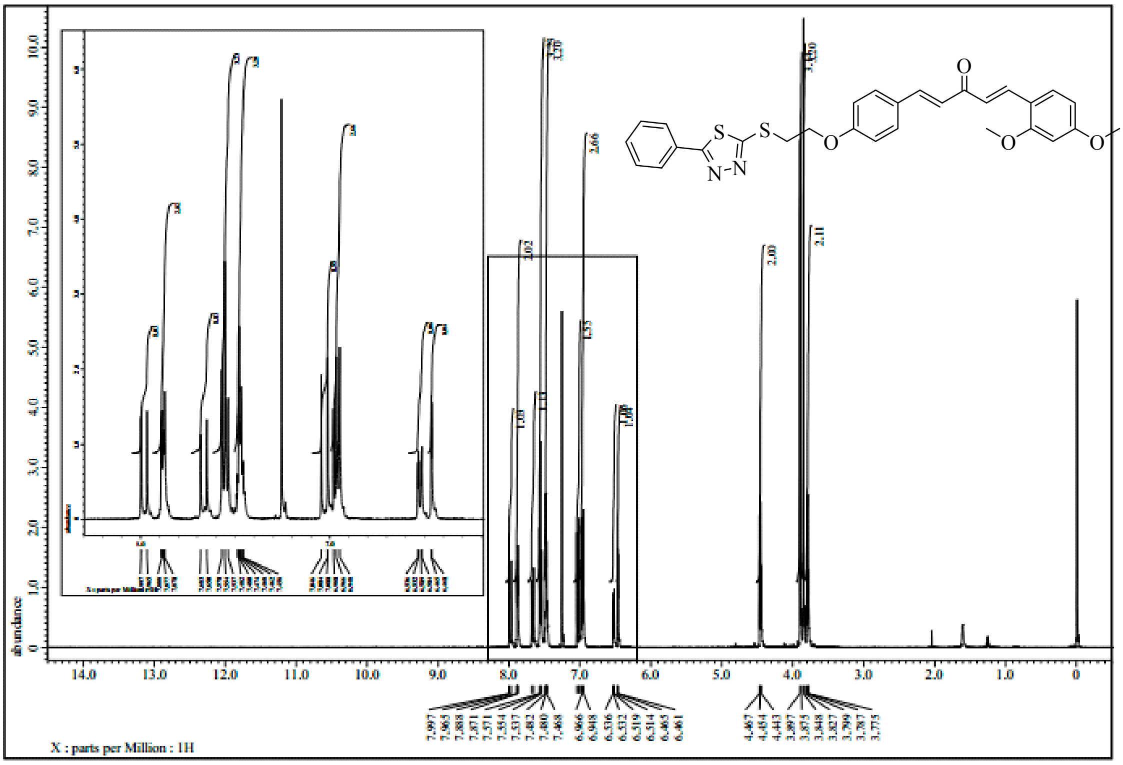

^{1}H NMR of compound **4e**

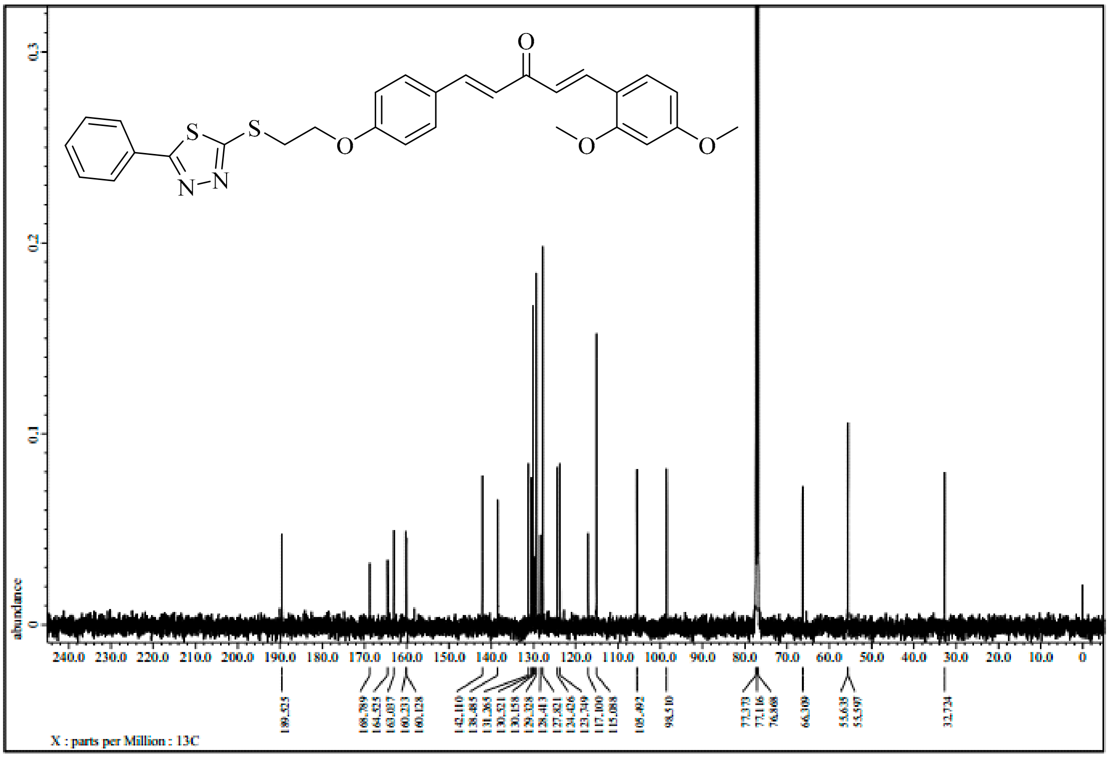

^{13}C NMR of compound **4e**

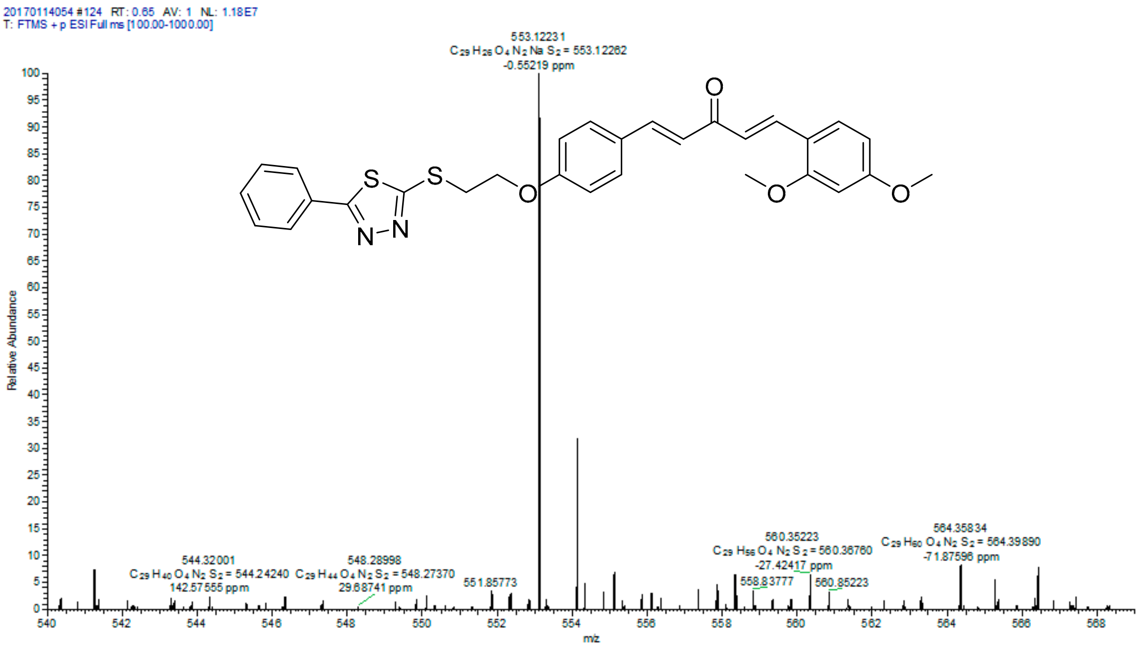

HRMS of compound **4e**

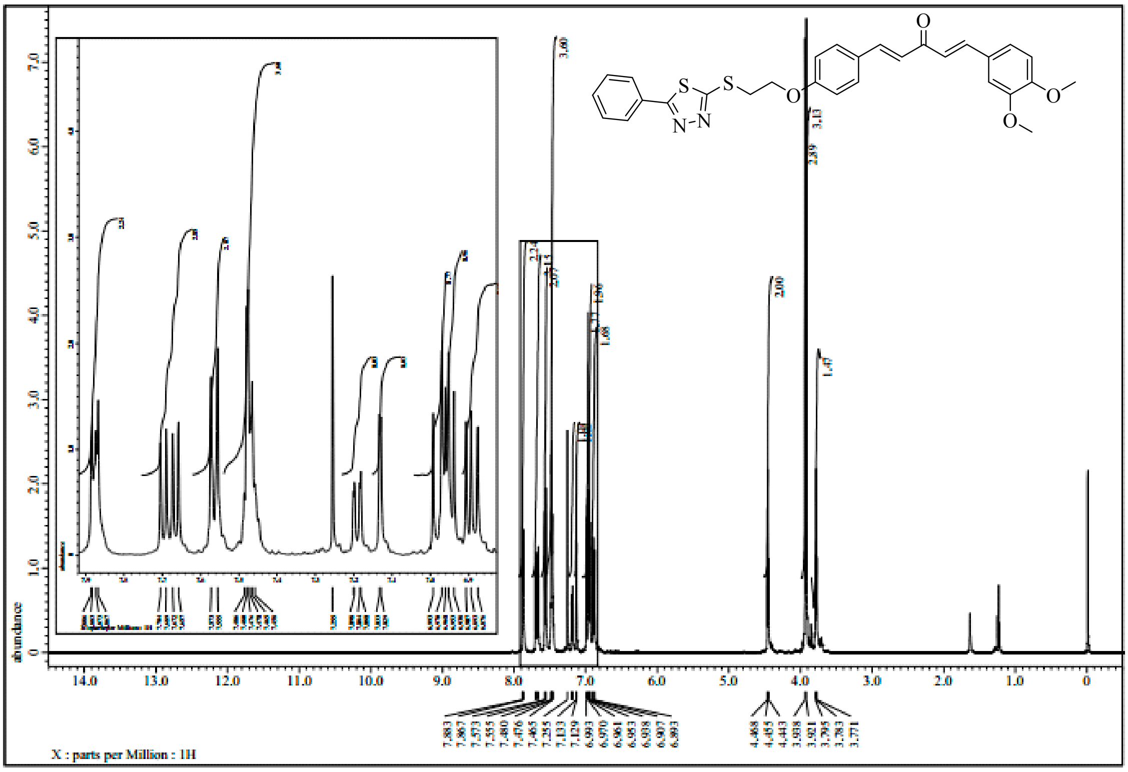

^{1}H NMR of compound **4f**

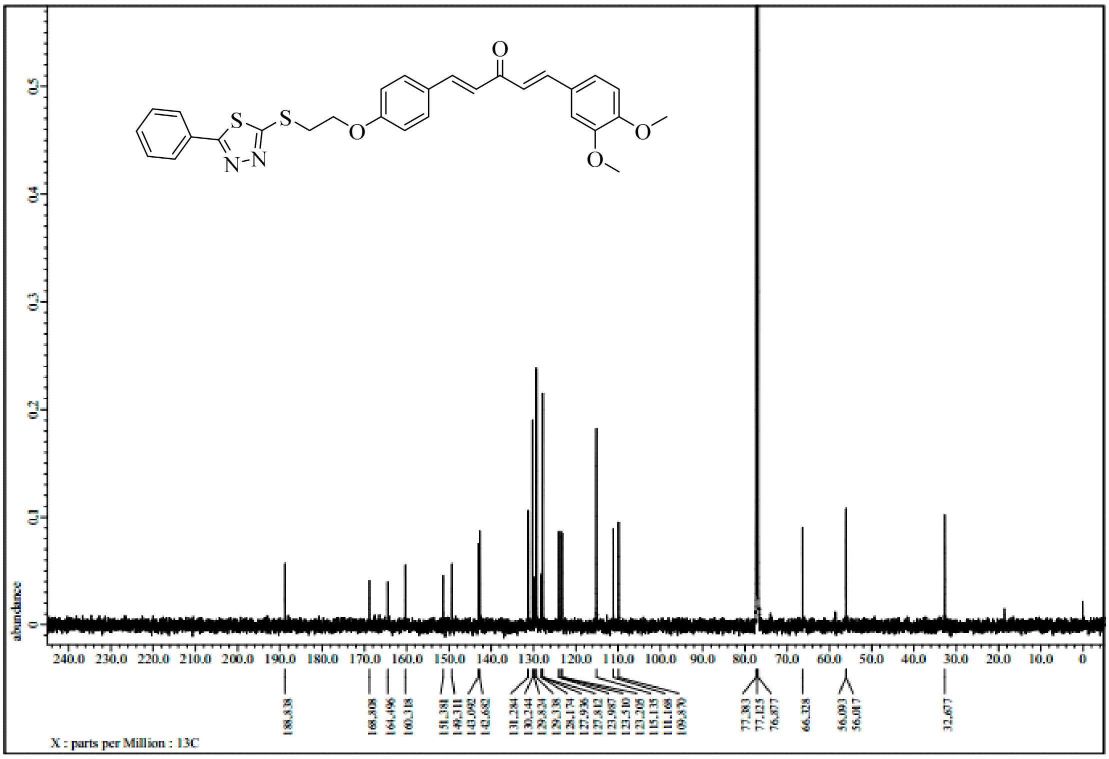

^{13}C NMR of compound **4f**

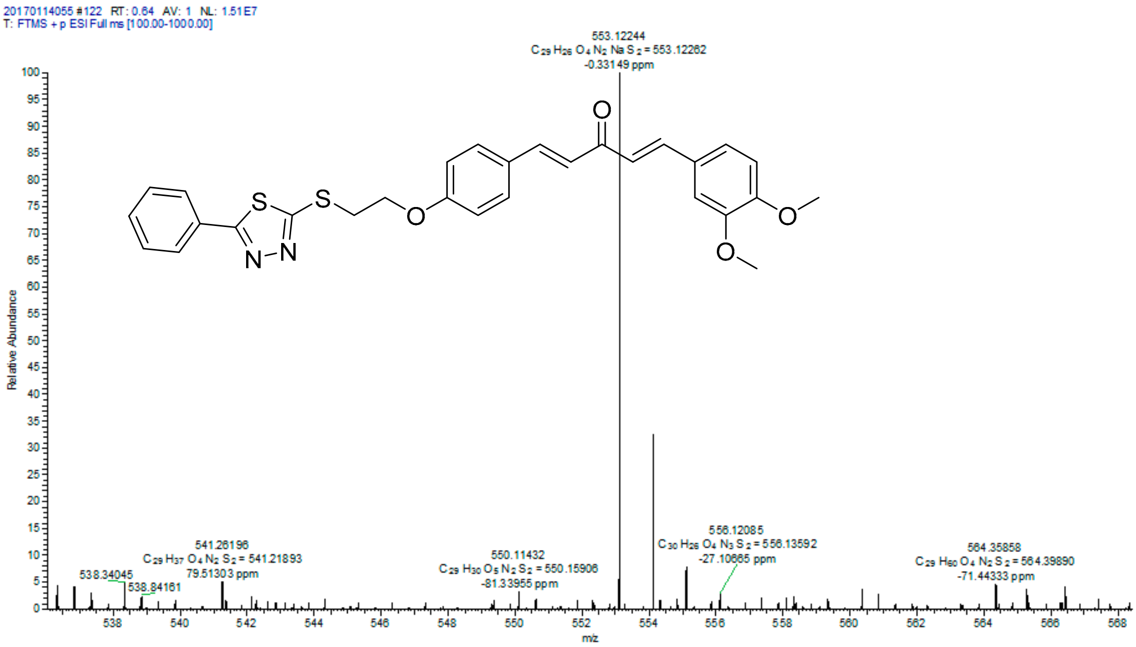

HRMS of compound **4f**

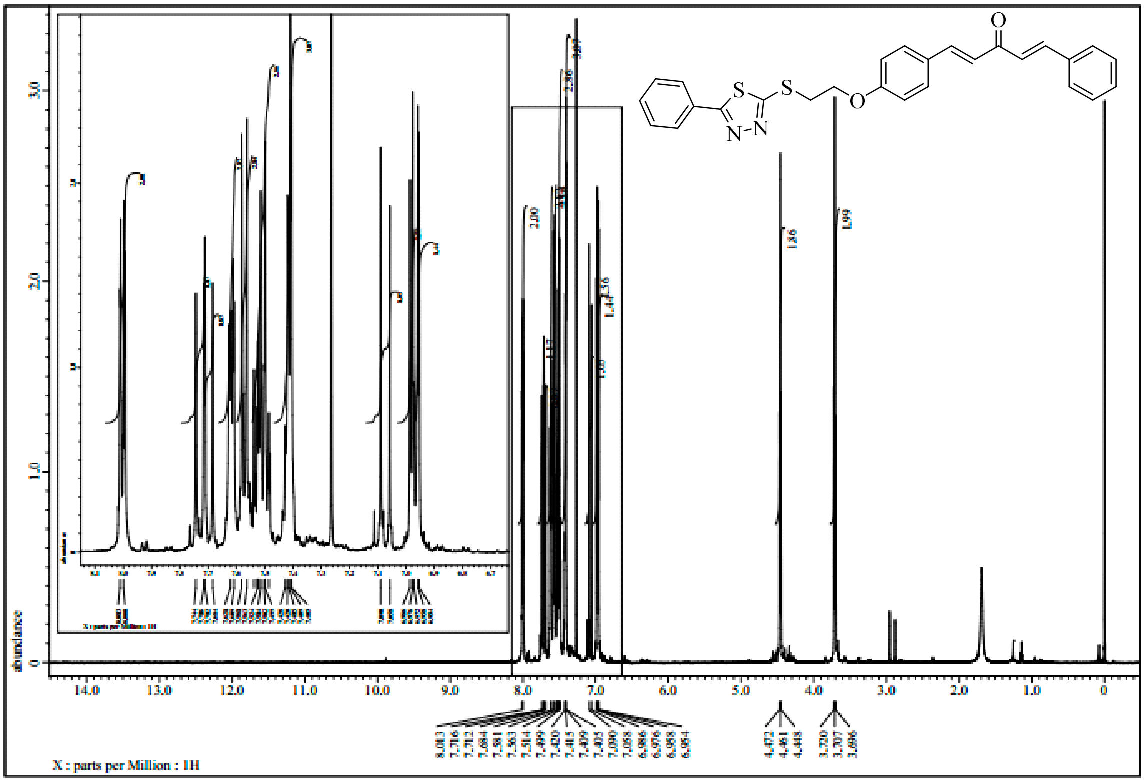

^{1}H NMR of compound **4g**

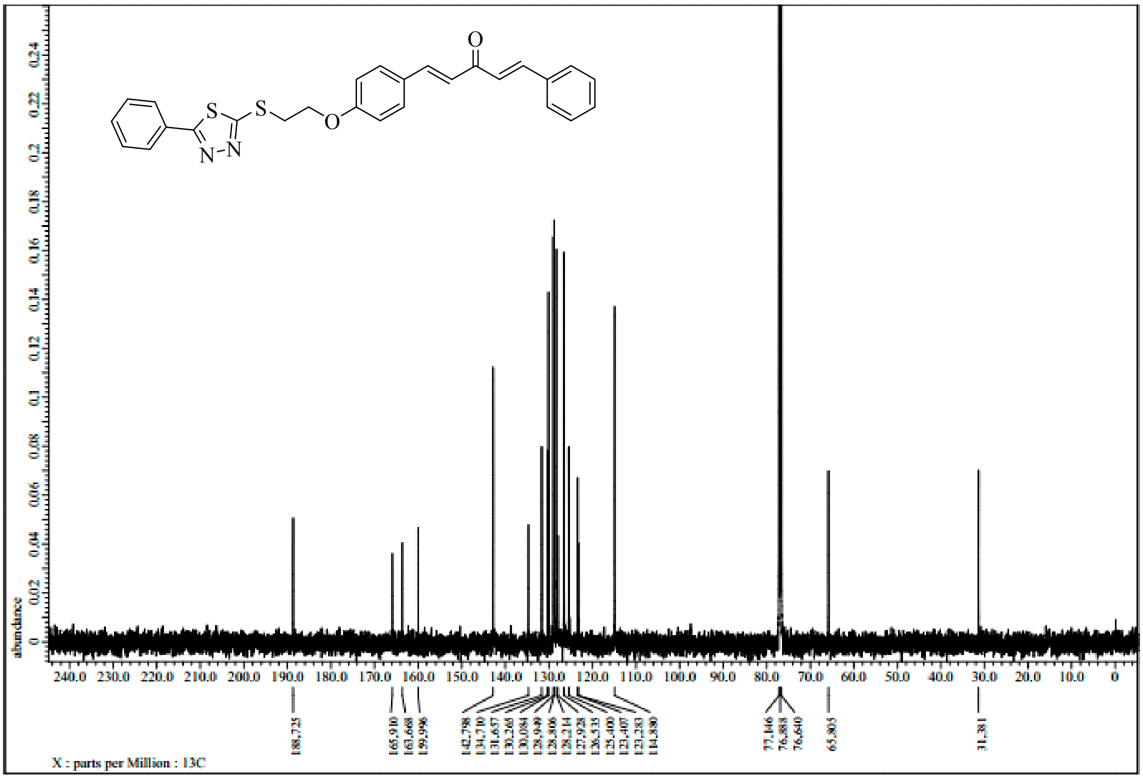

^{13}C NMR of compound **4g**

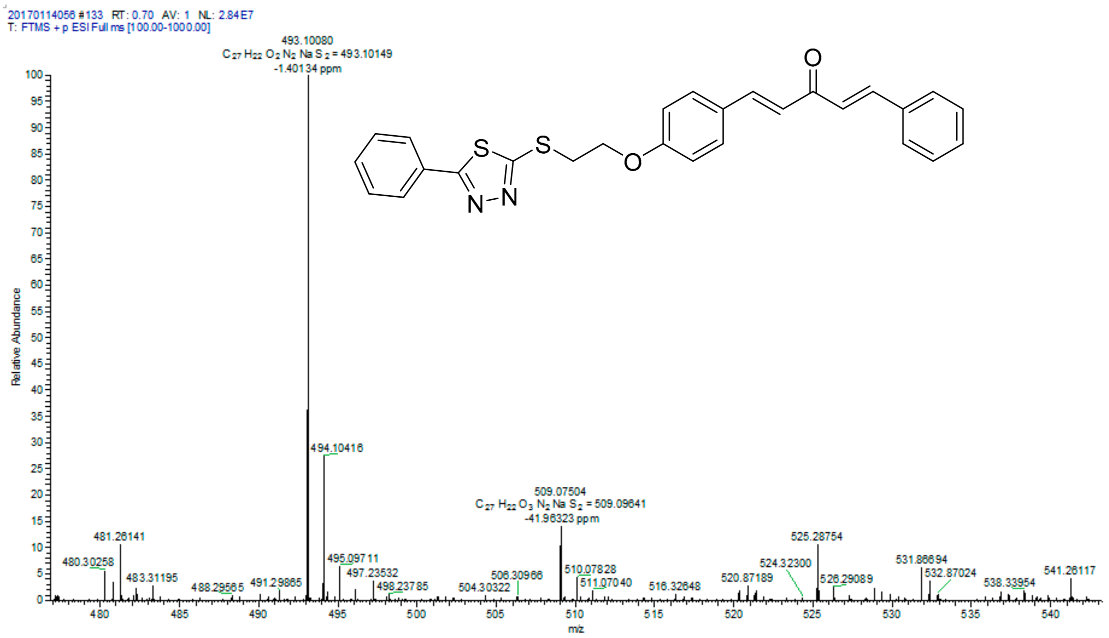

HRMS of compound **4g**

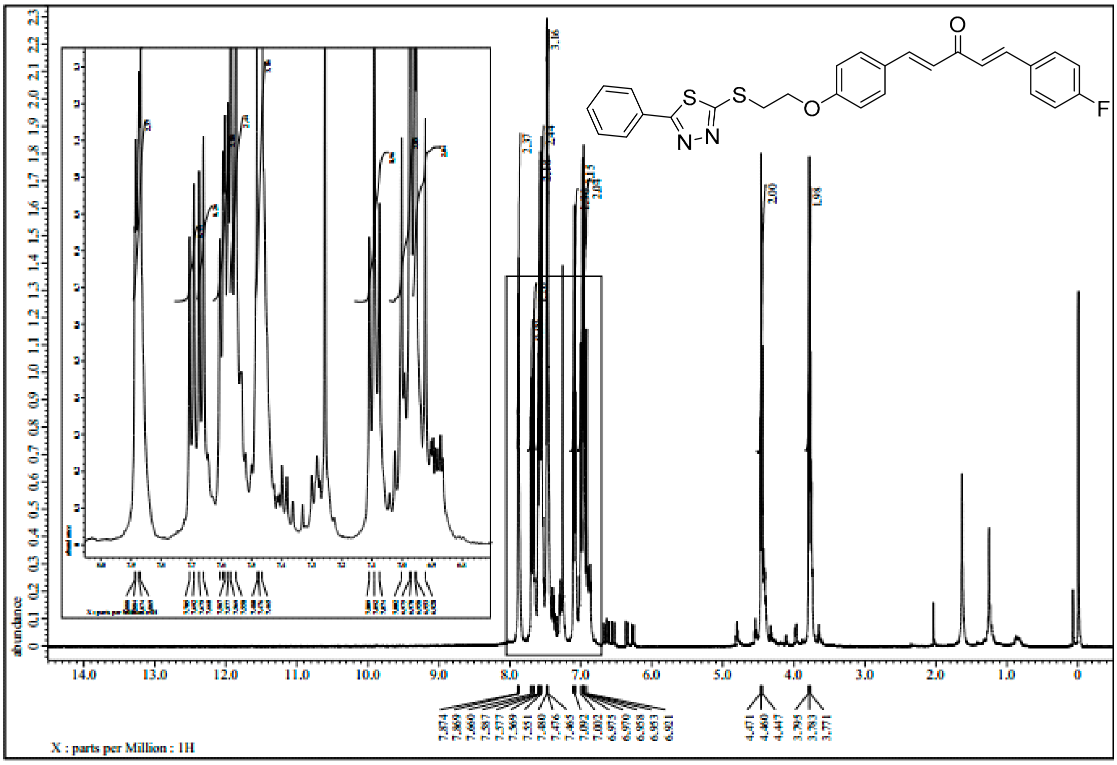

^{1}H NMR of compound **4h**

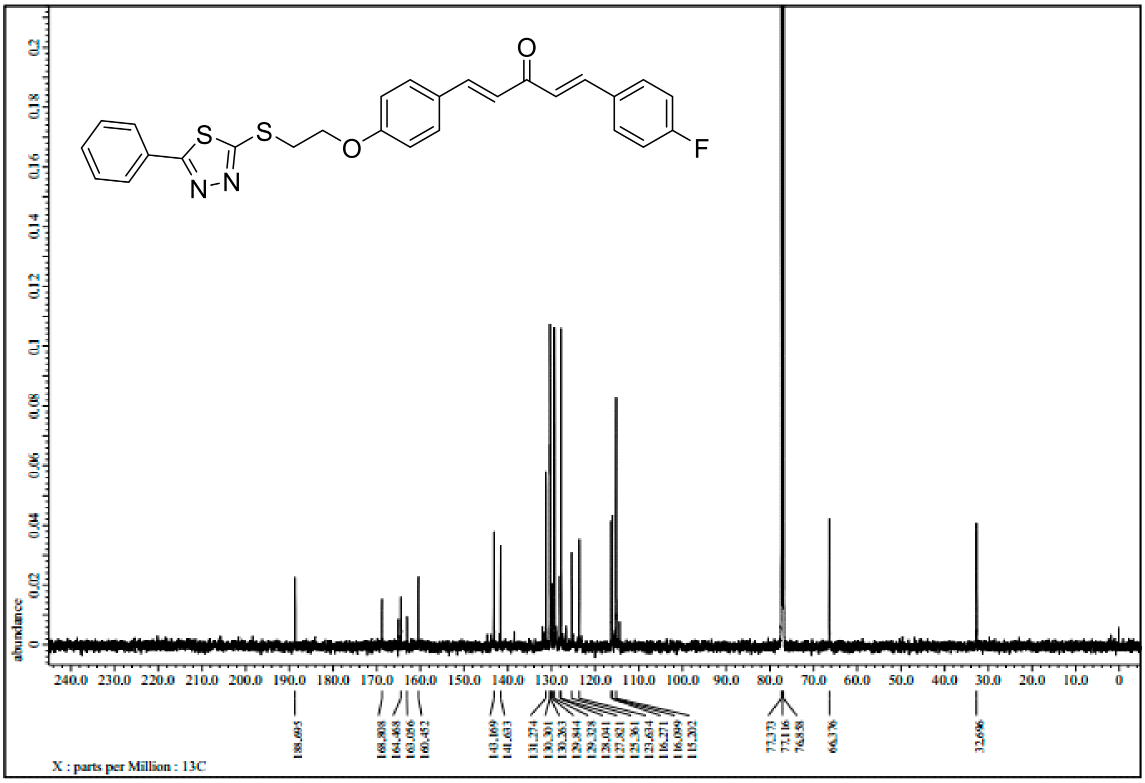

^{13}C NMR of compound **4h**

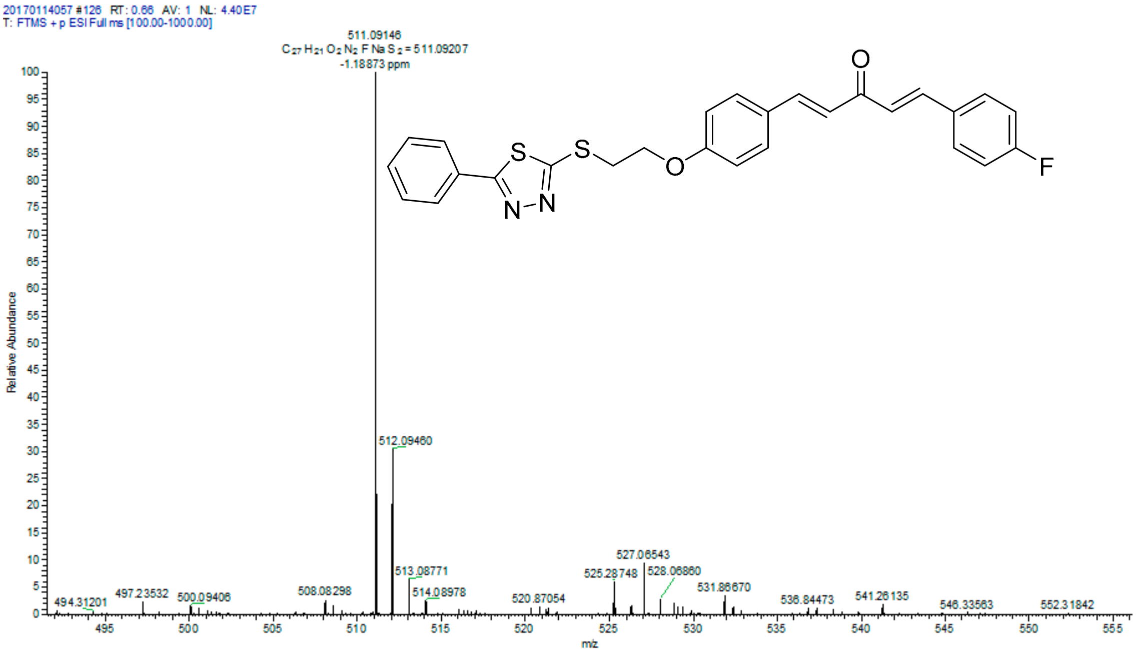

HRMS of compound **4h**

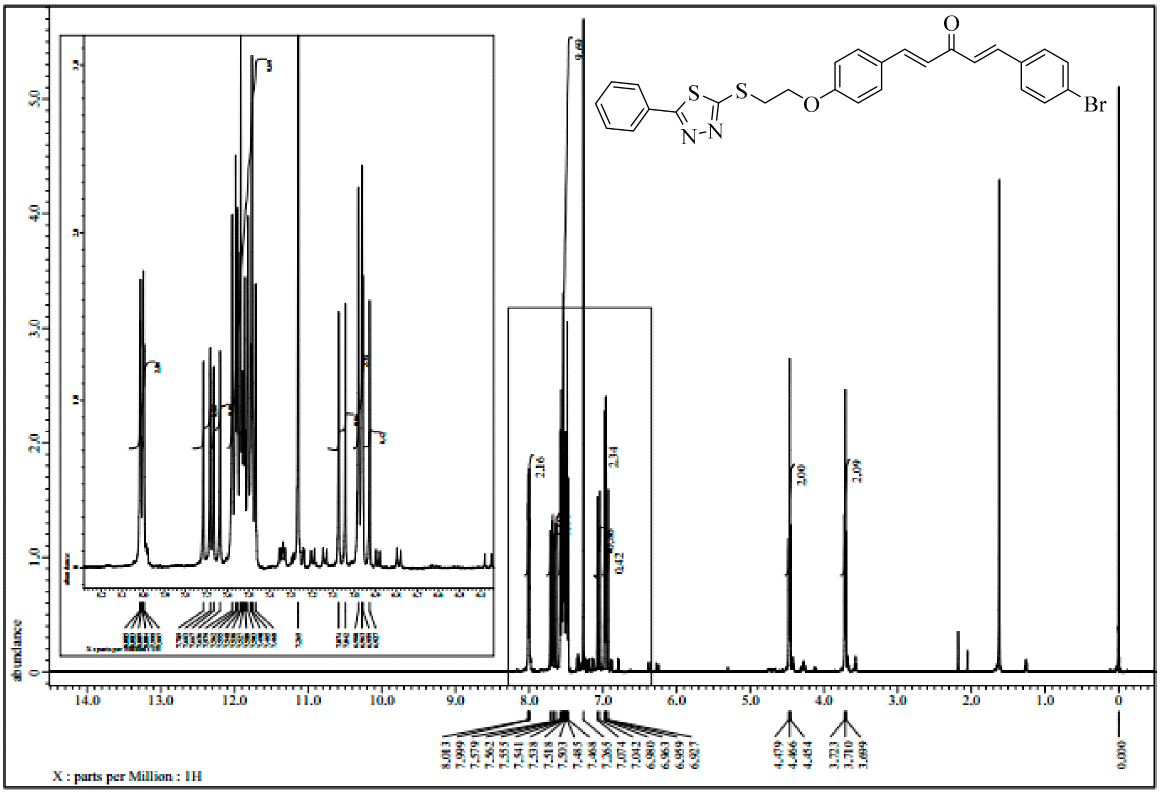

^{1}H NMR of compound **4i**

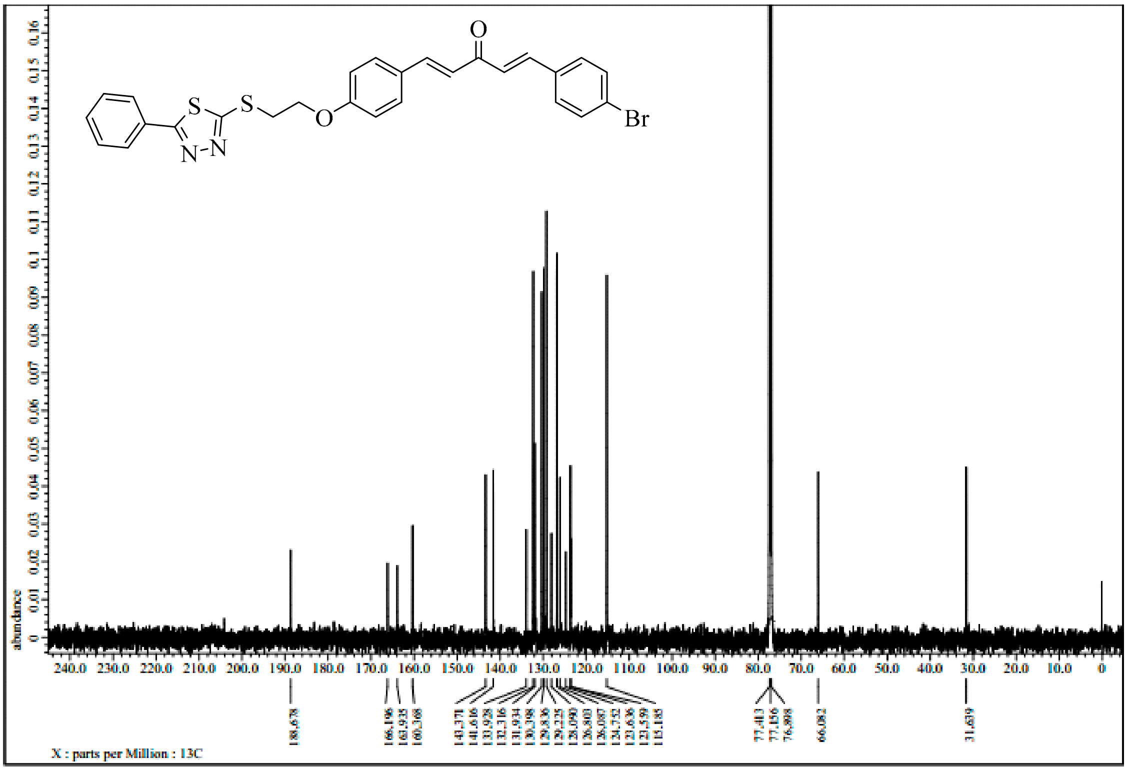

^{13}C NMR of compound **4i**

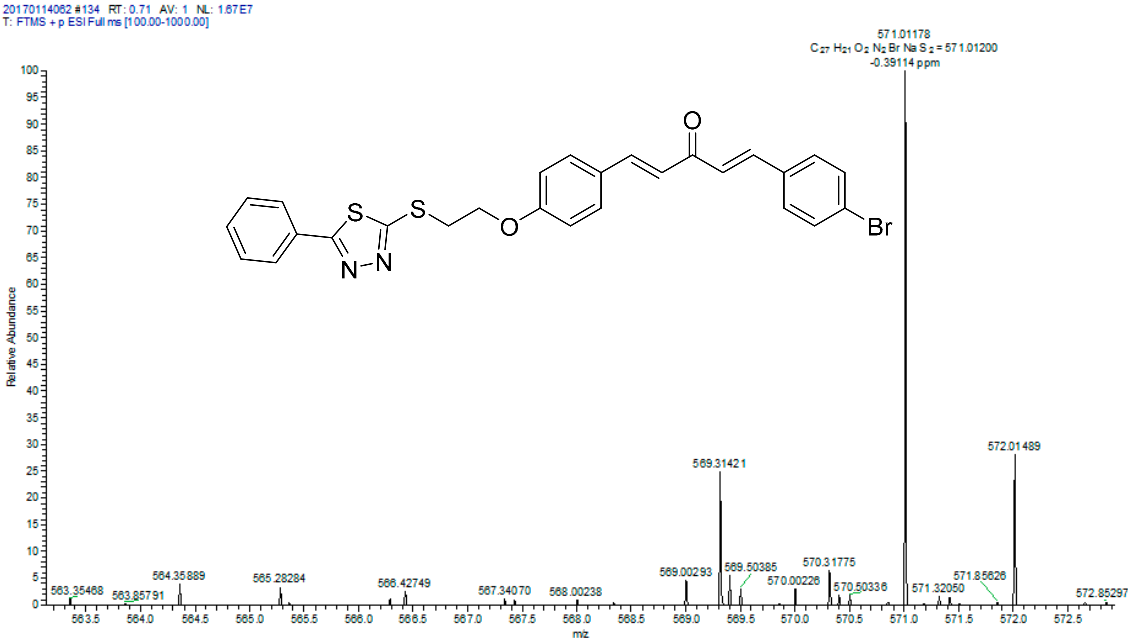

HRMS of compound **4i**

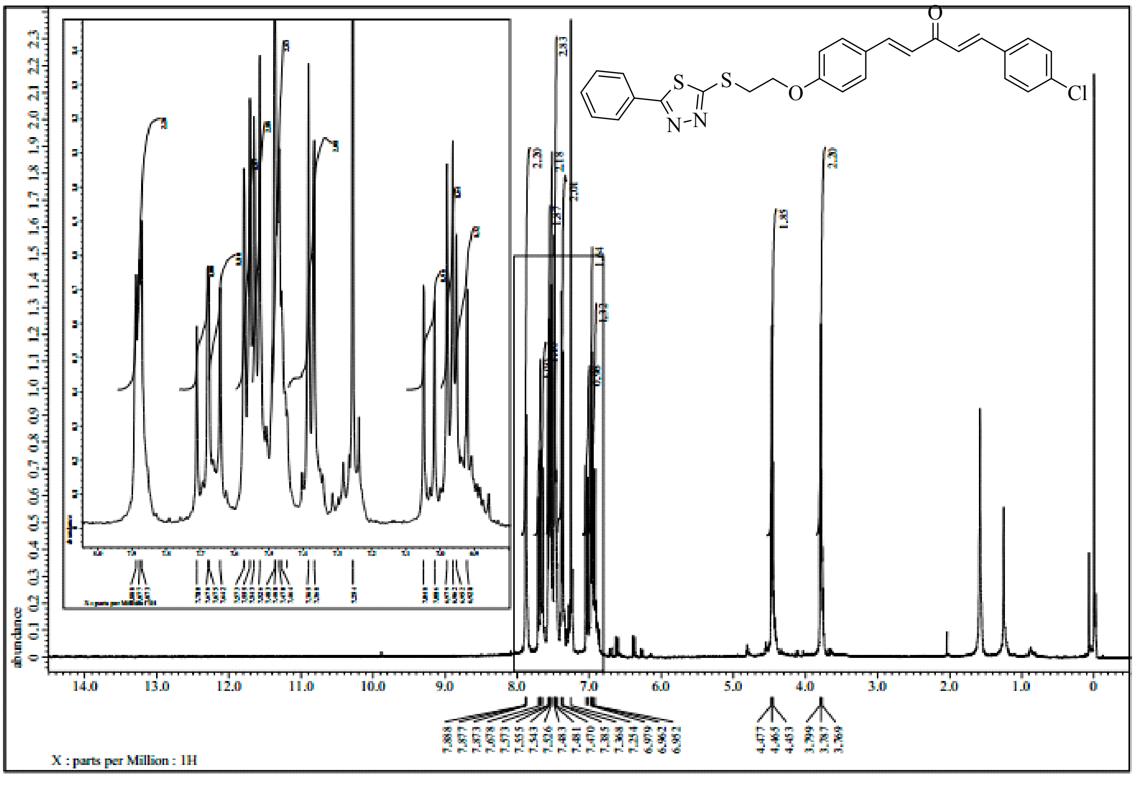

^{1}H NMR of compound **4j**

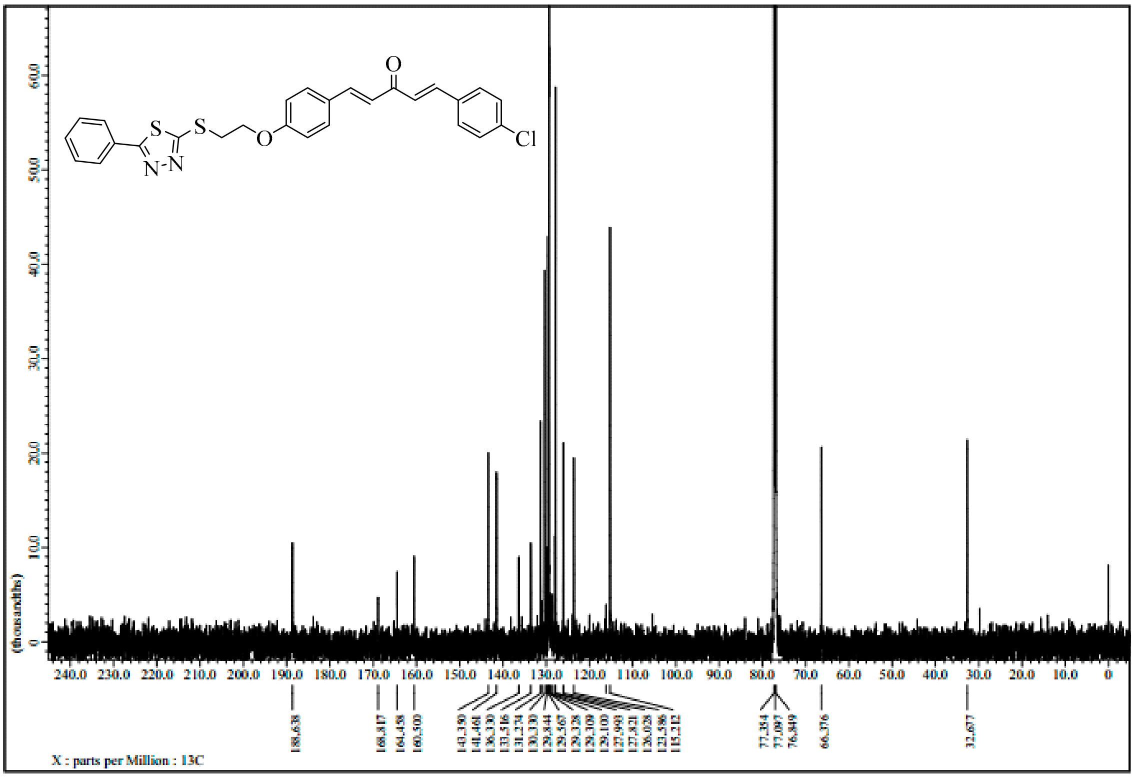

^{13}C NMR of compound **4j**

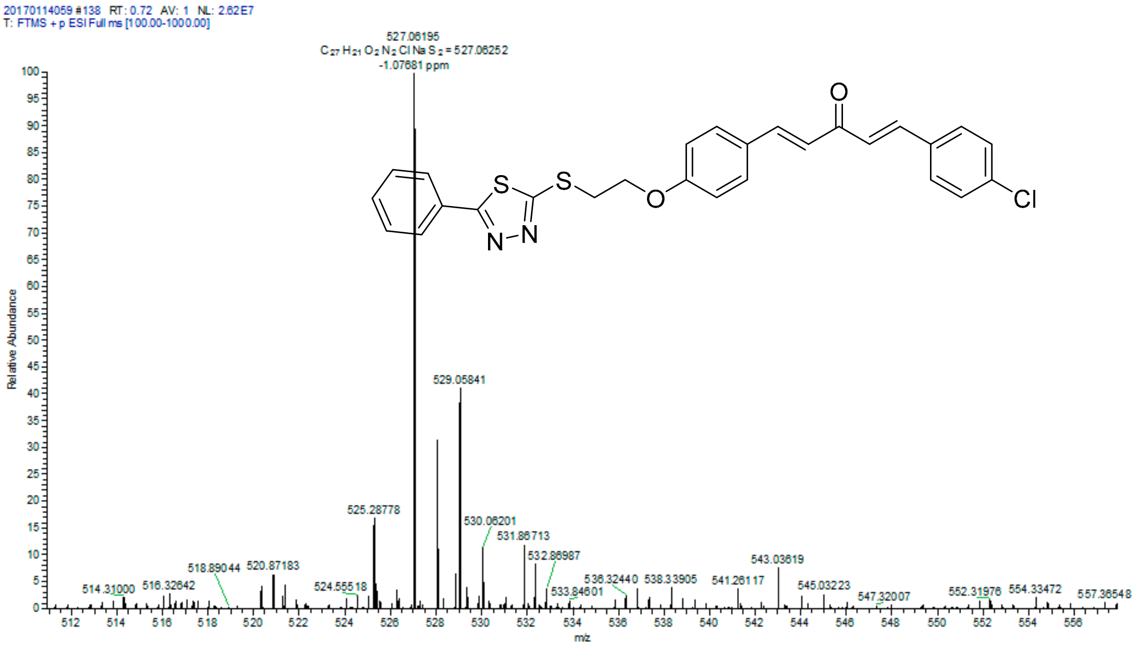

HRMS of compound **4j**

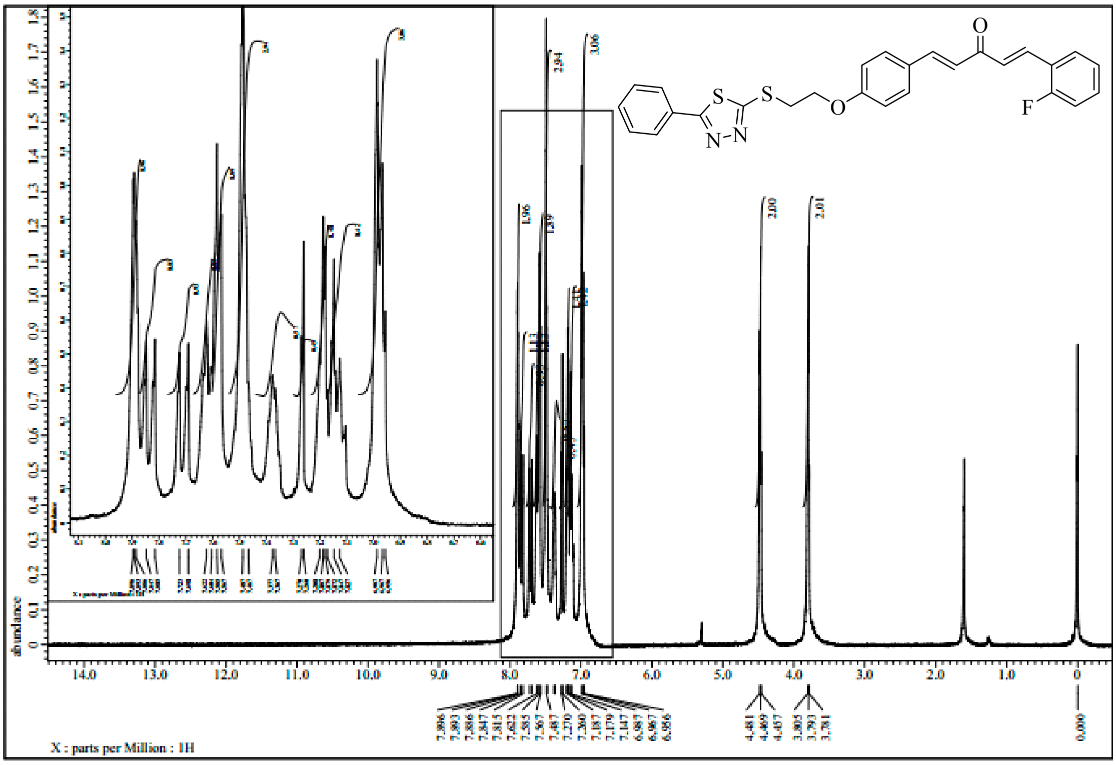

^{1}H NMR of compound **4k**

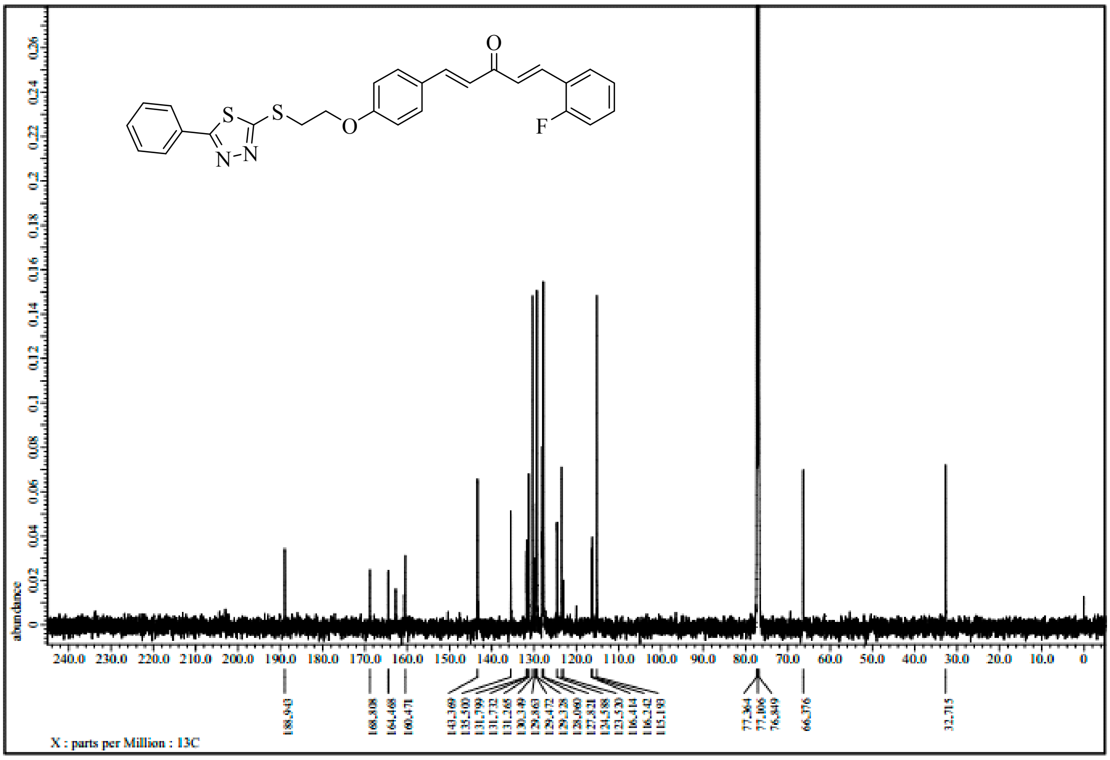

^{13}C NMR of compound **4k**

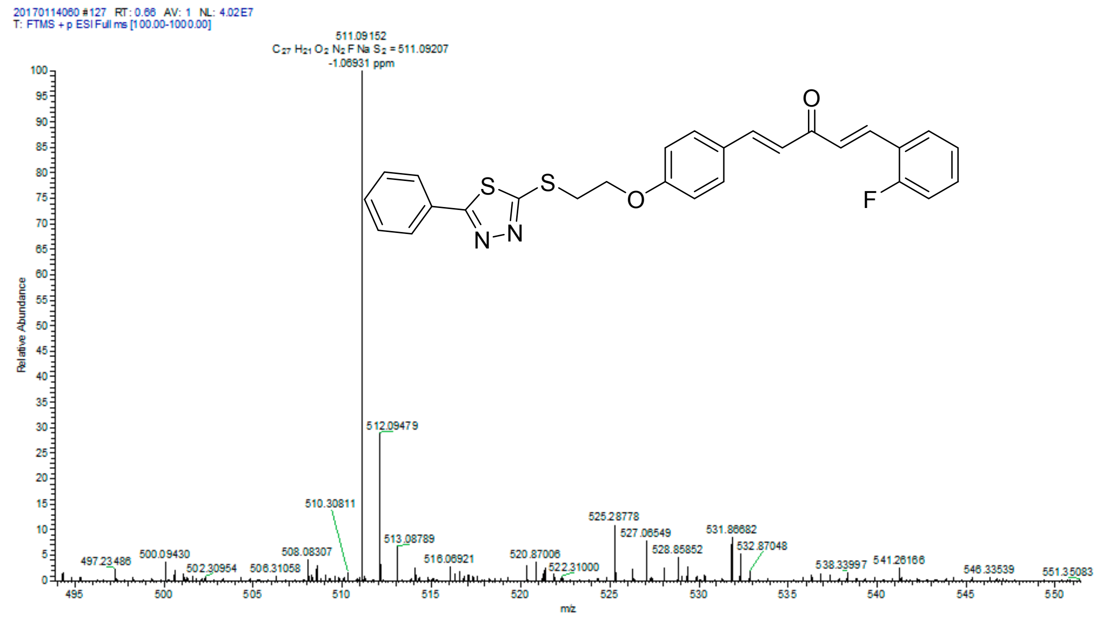

HRMS of compound **4k**

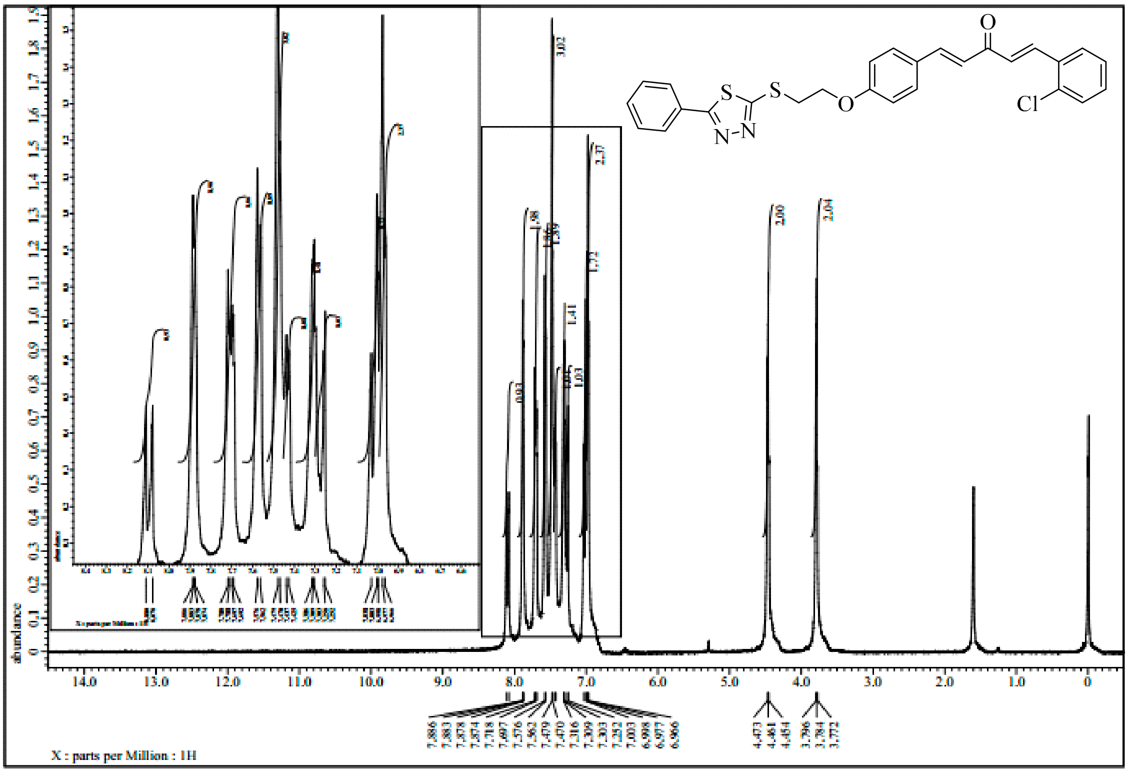

^{1}H NMR of compound **4l**

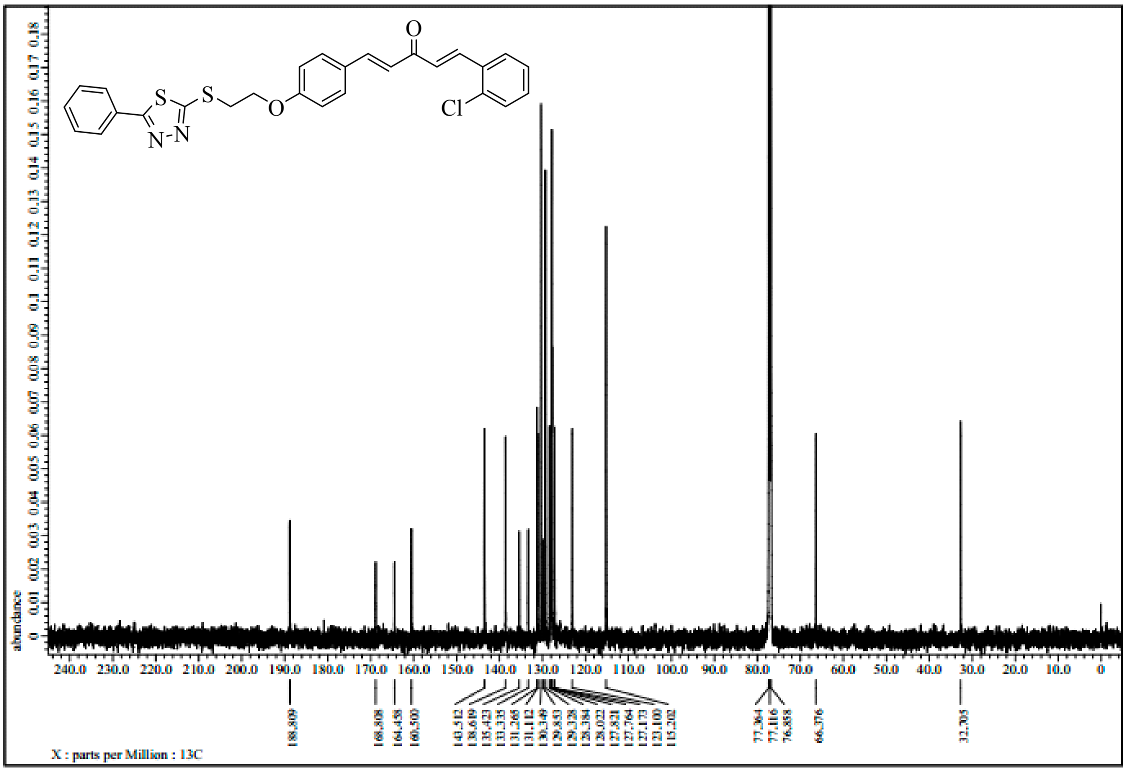

^{13}C NMR of compound **4l**

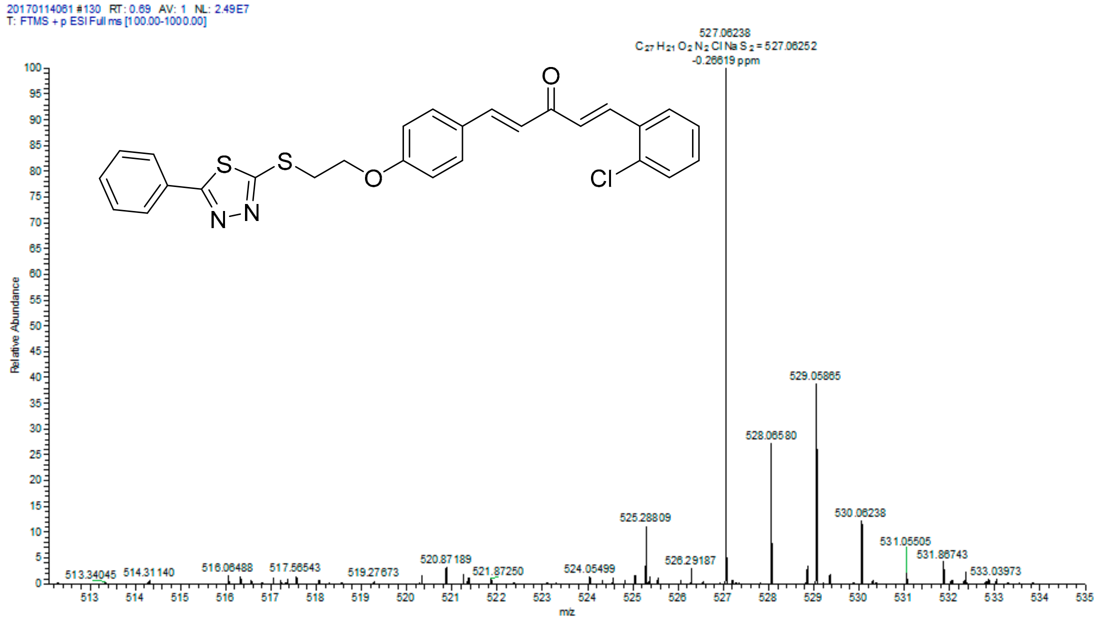

HRMS of compound **4l**

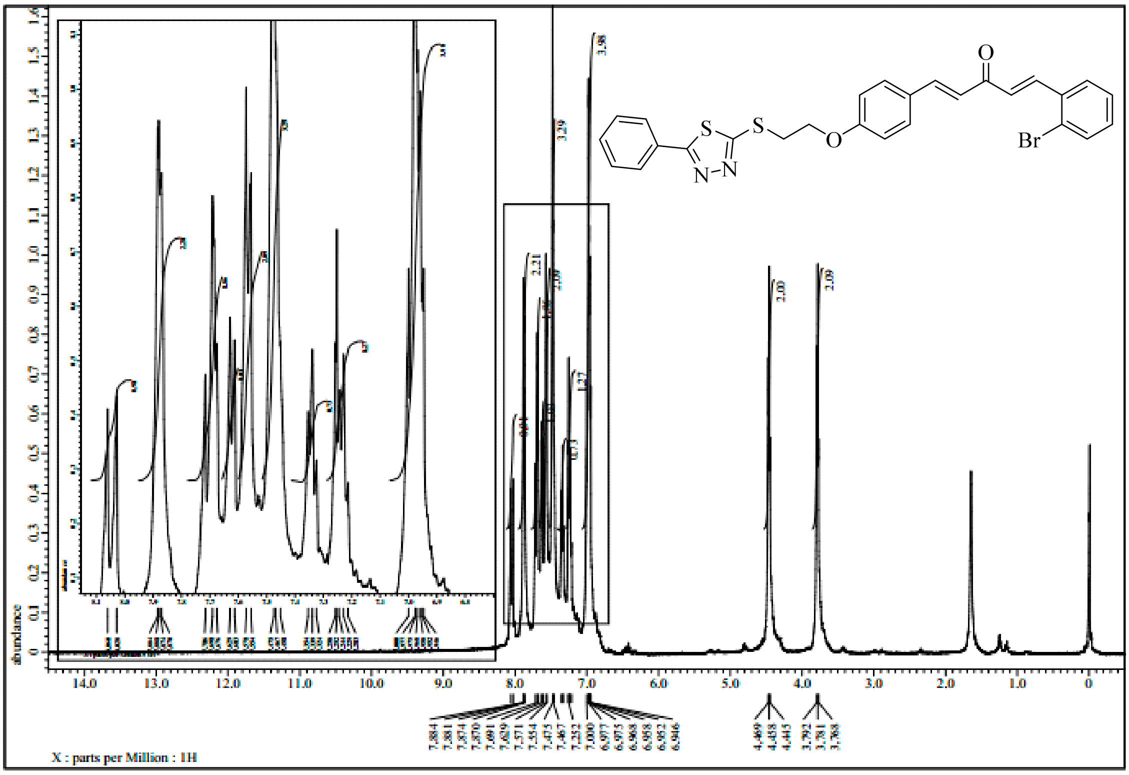

^{1}H NMR of compound **4m**

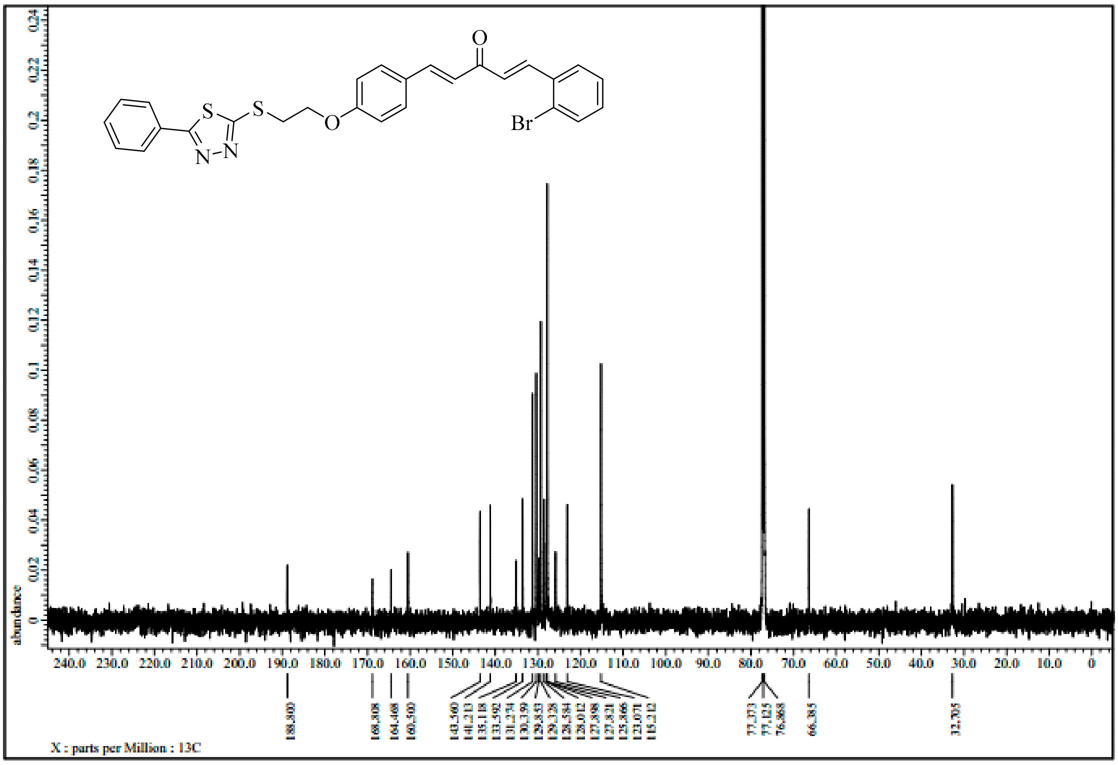

^{13}C NMR of compound **4m**

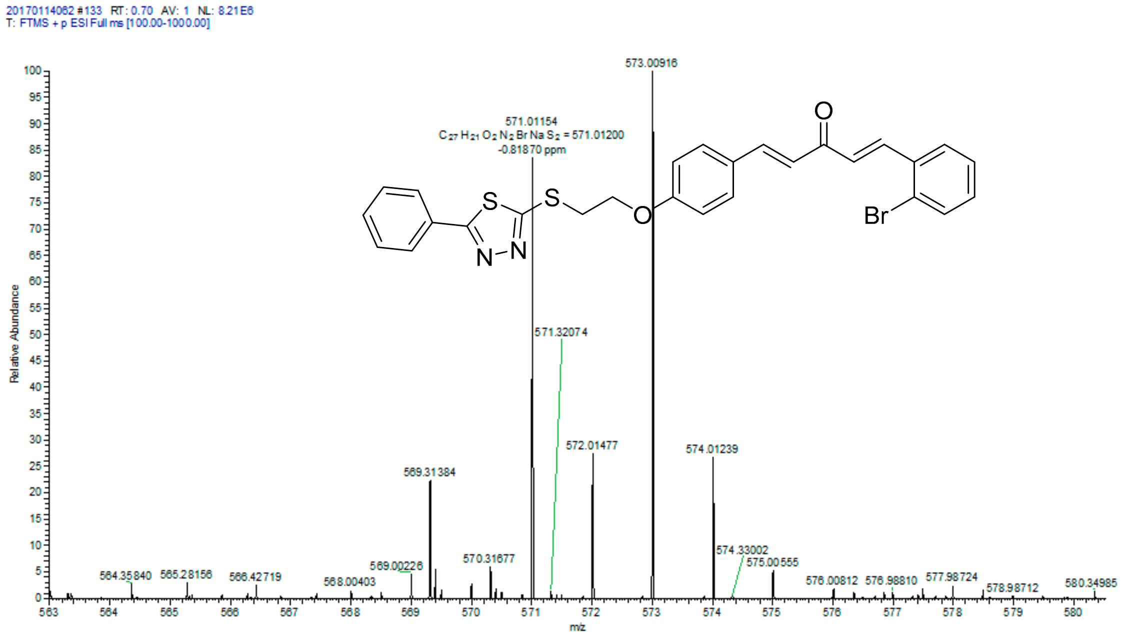

HRMS of compound **4m**

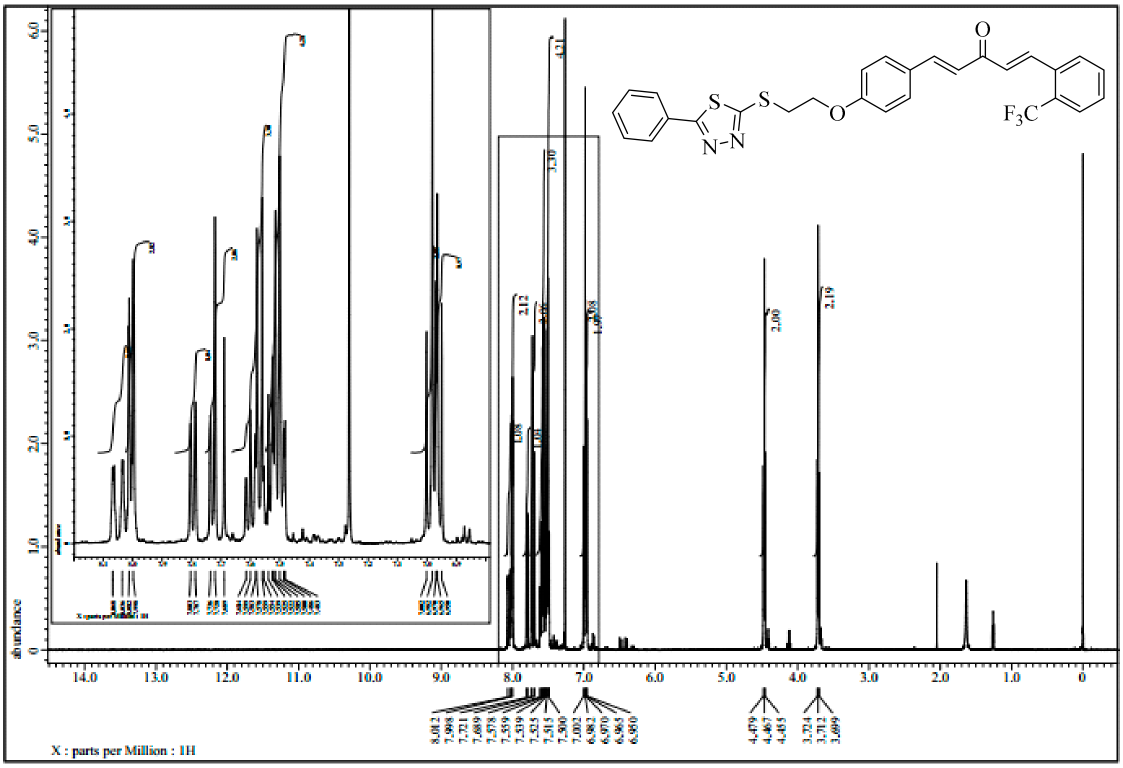

^{1}H NMR of compound **4n**

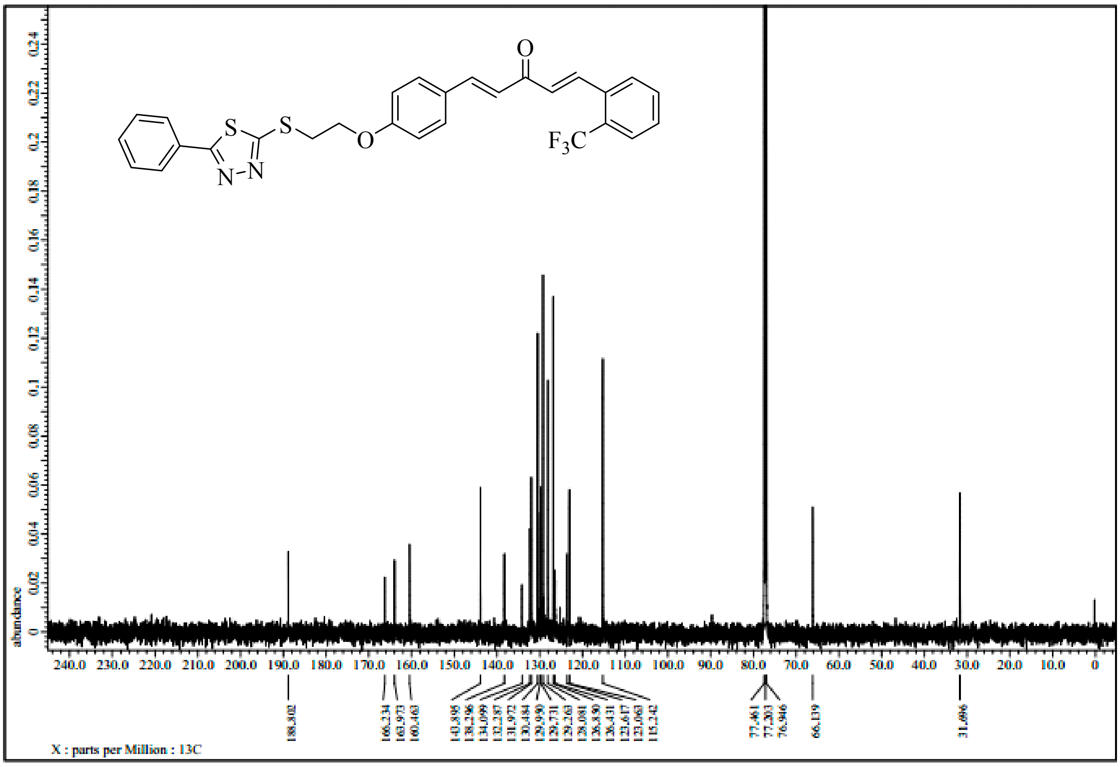

^{13}C NMR of compound **4n**

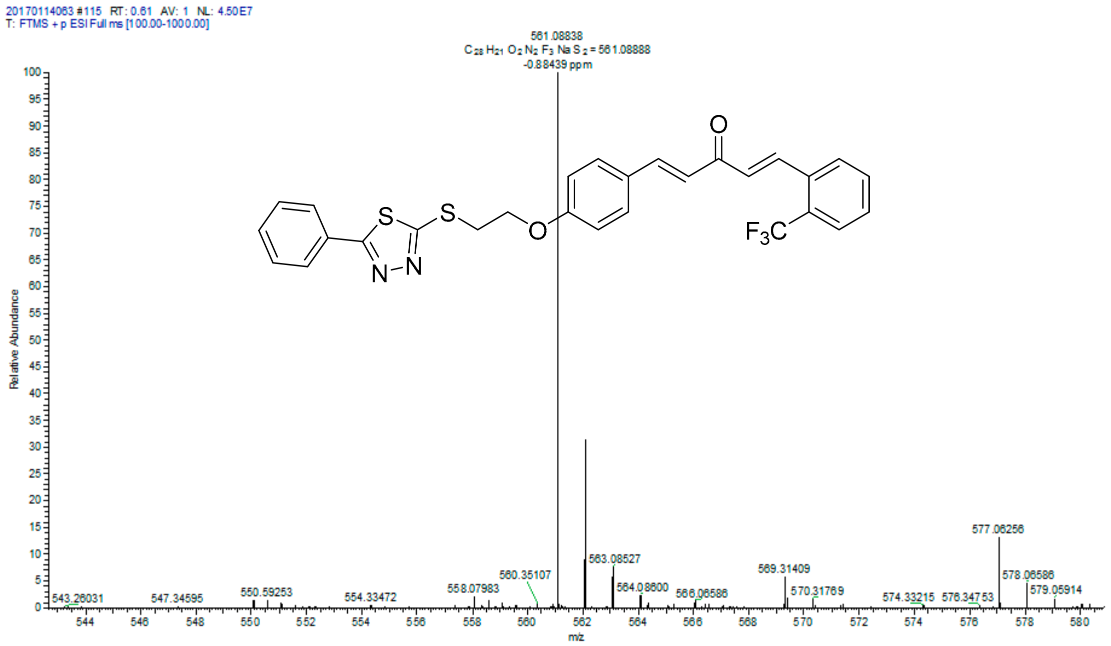

HRMS of compound **4n**

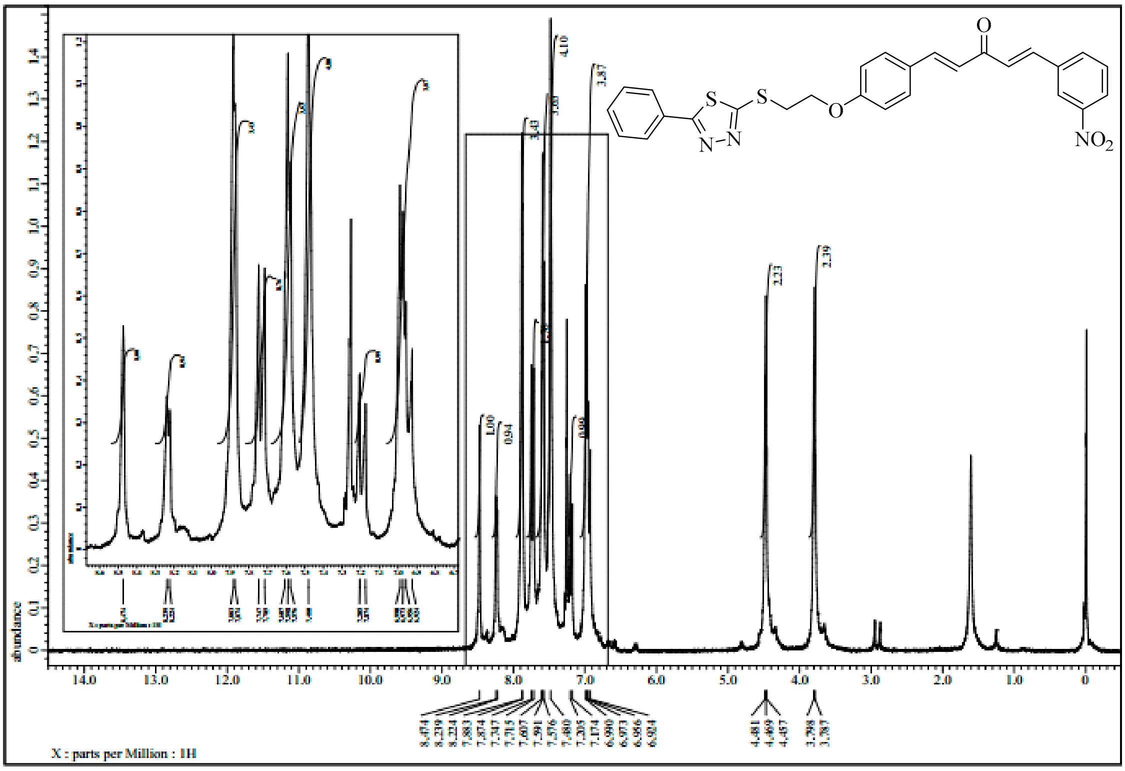

^{1}H NMR of compound **4o**

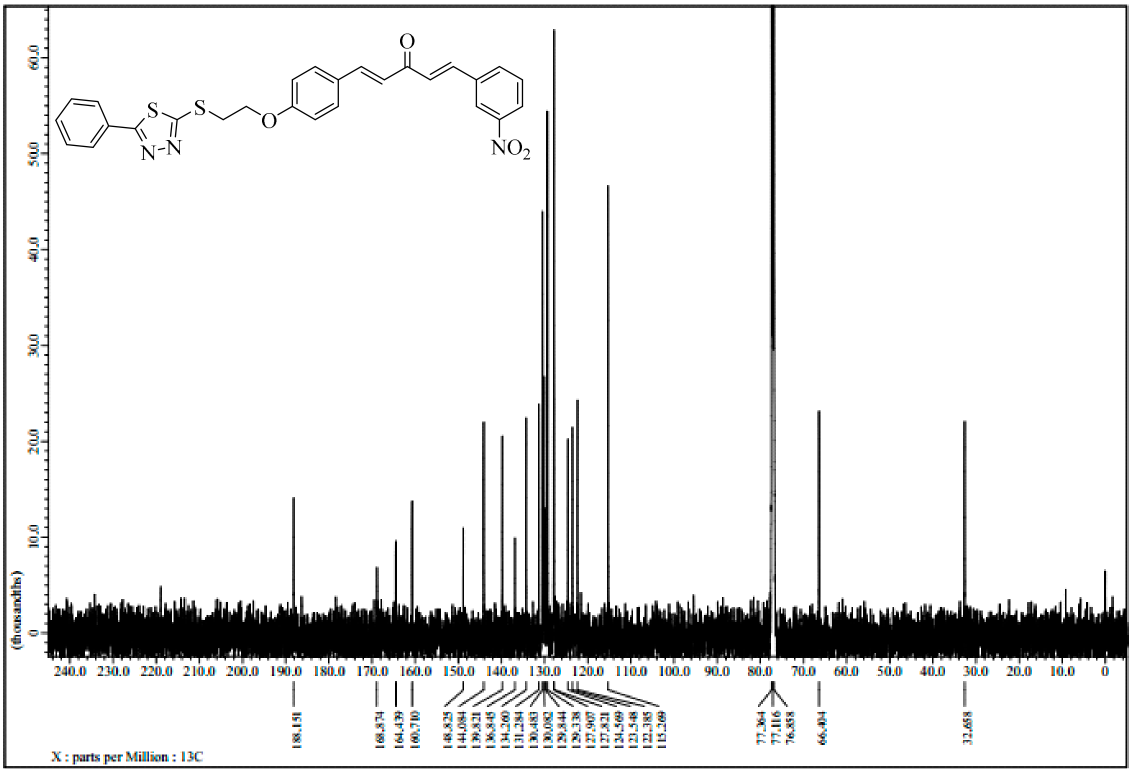

^{13}C NMR of compound **4o**

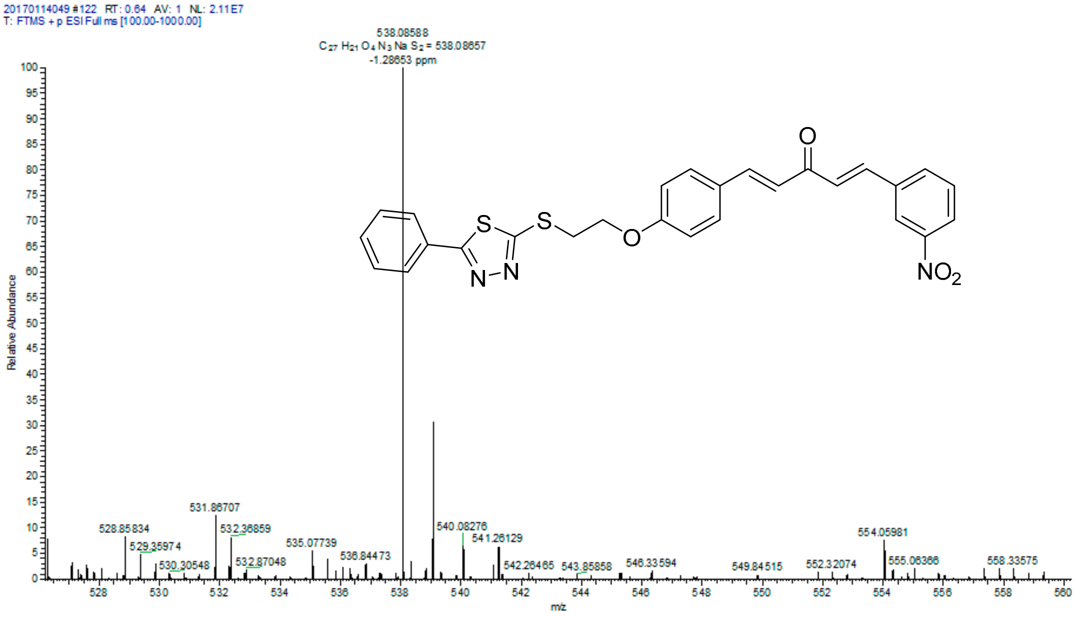

HRMS of compound **4o**

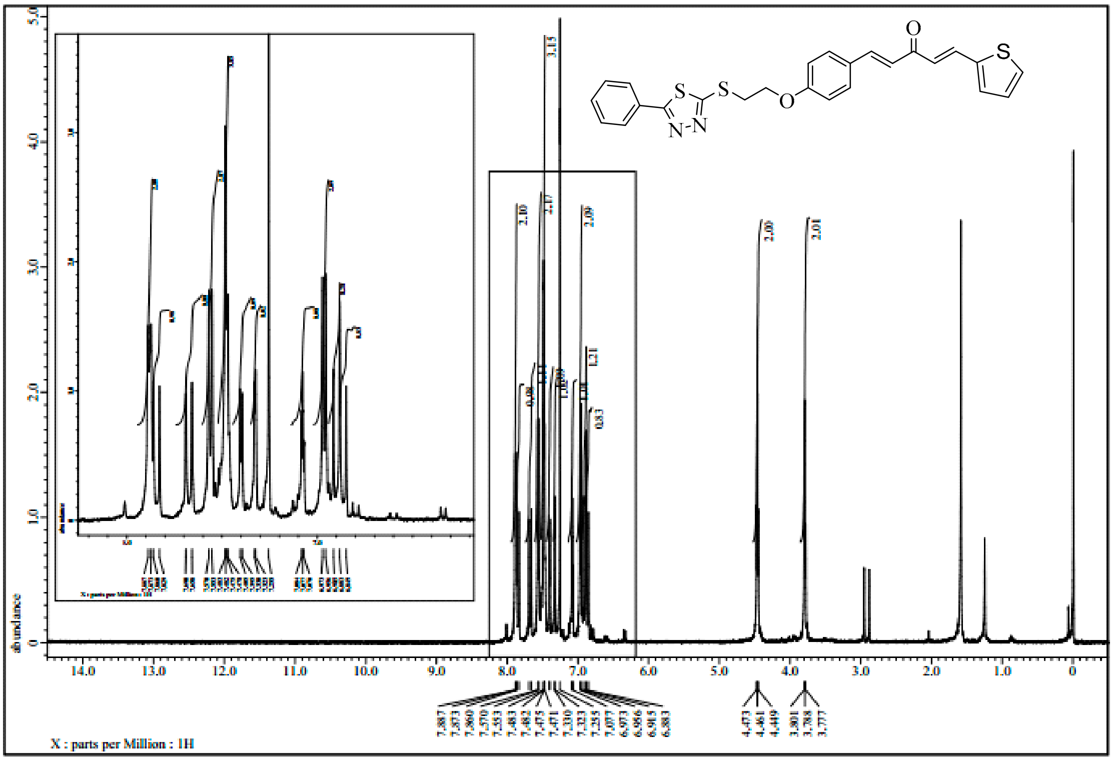

^{1}H NMR of compound **4p**

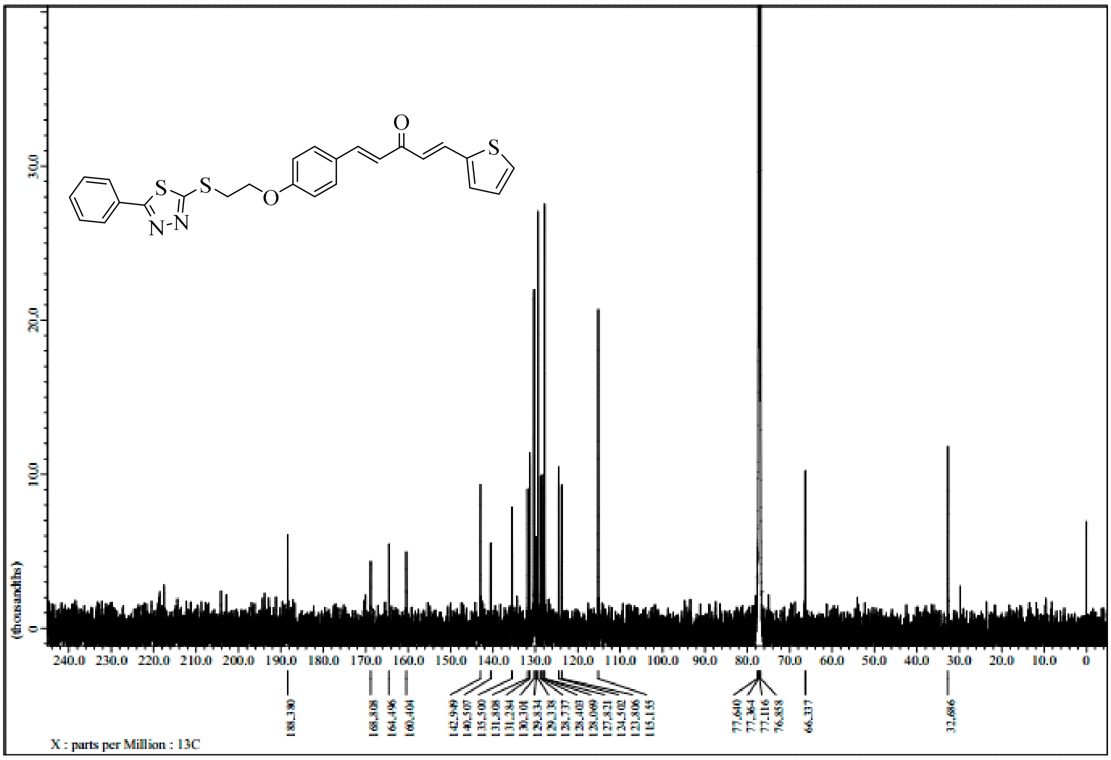

^{13}C NMR of compound **4p**

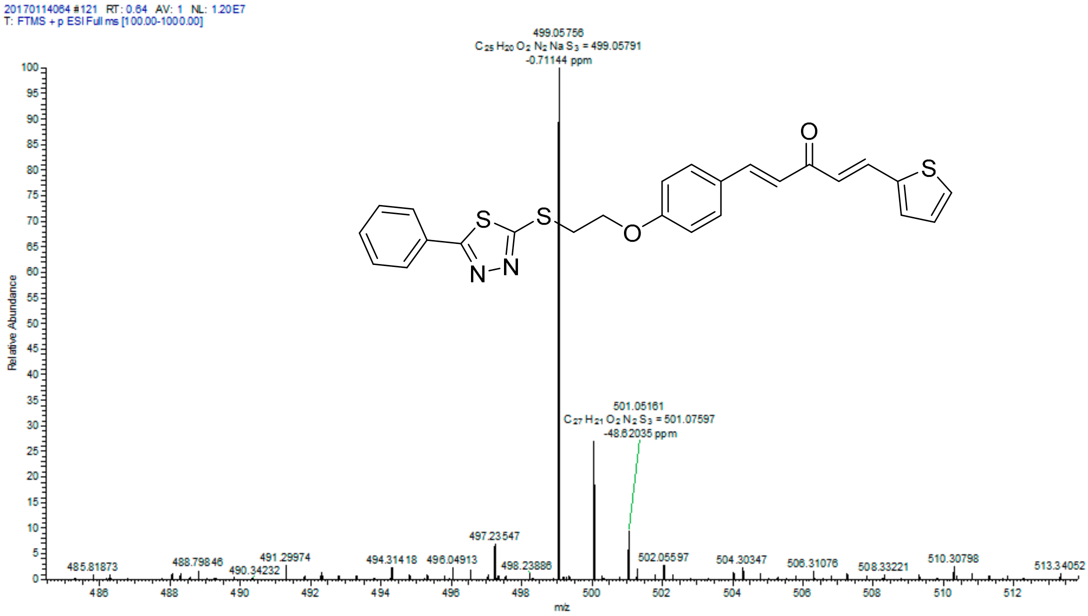

HRMS of compound **4p**

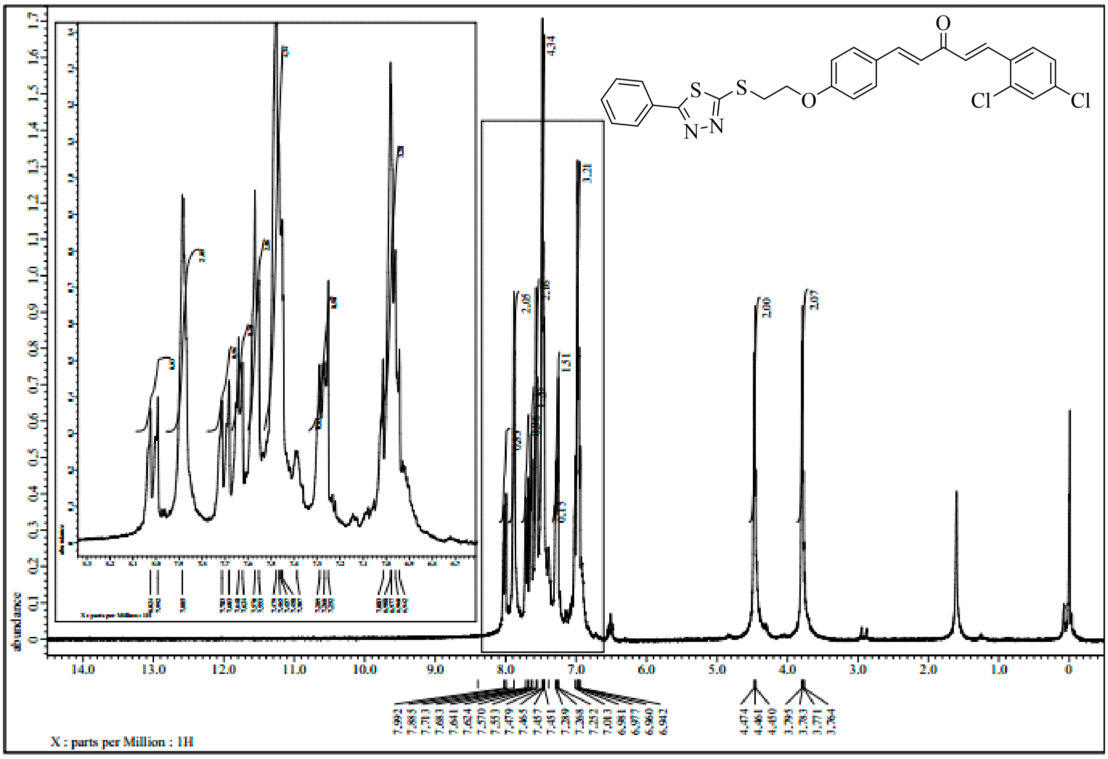

^{1}H NMR of compound **4q**

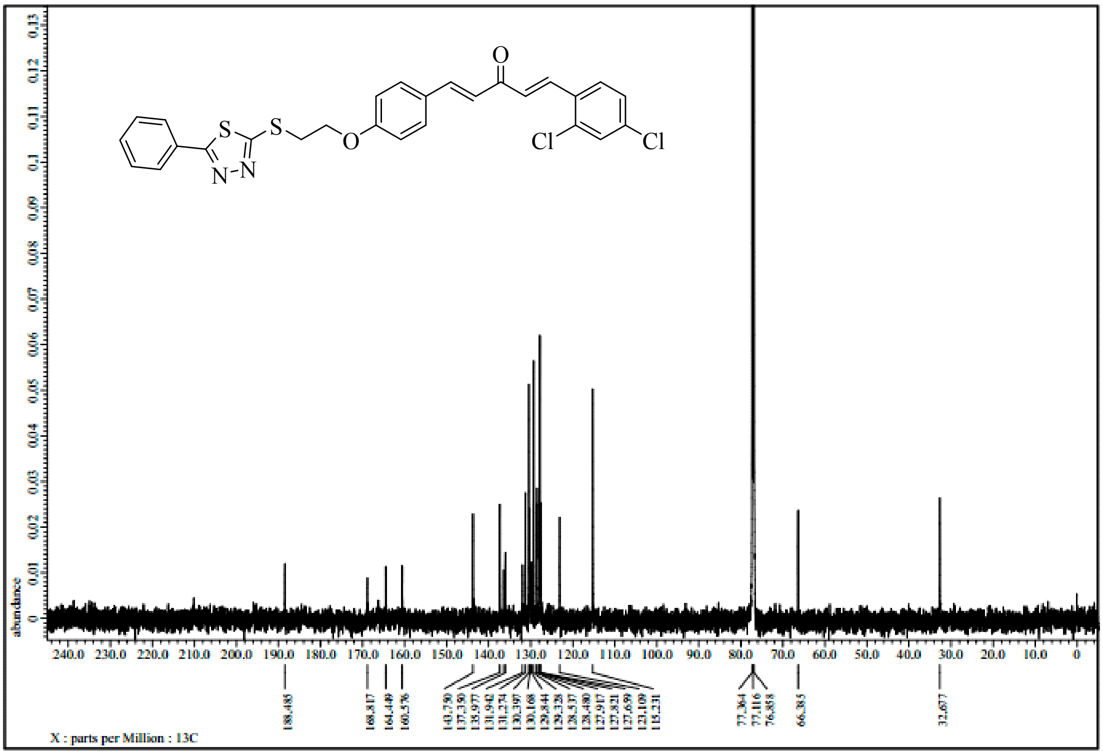

^{13}C NMR of compound **4q**

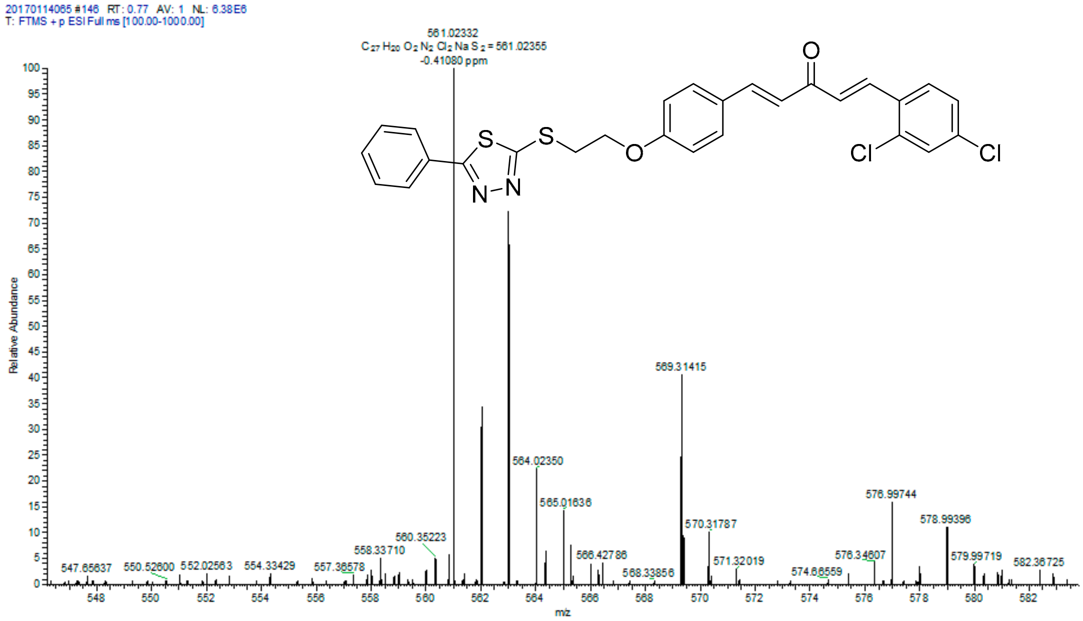

HRMS of compound **4q**

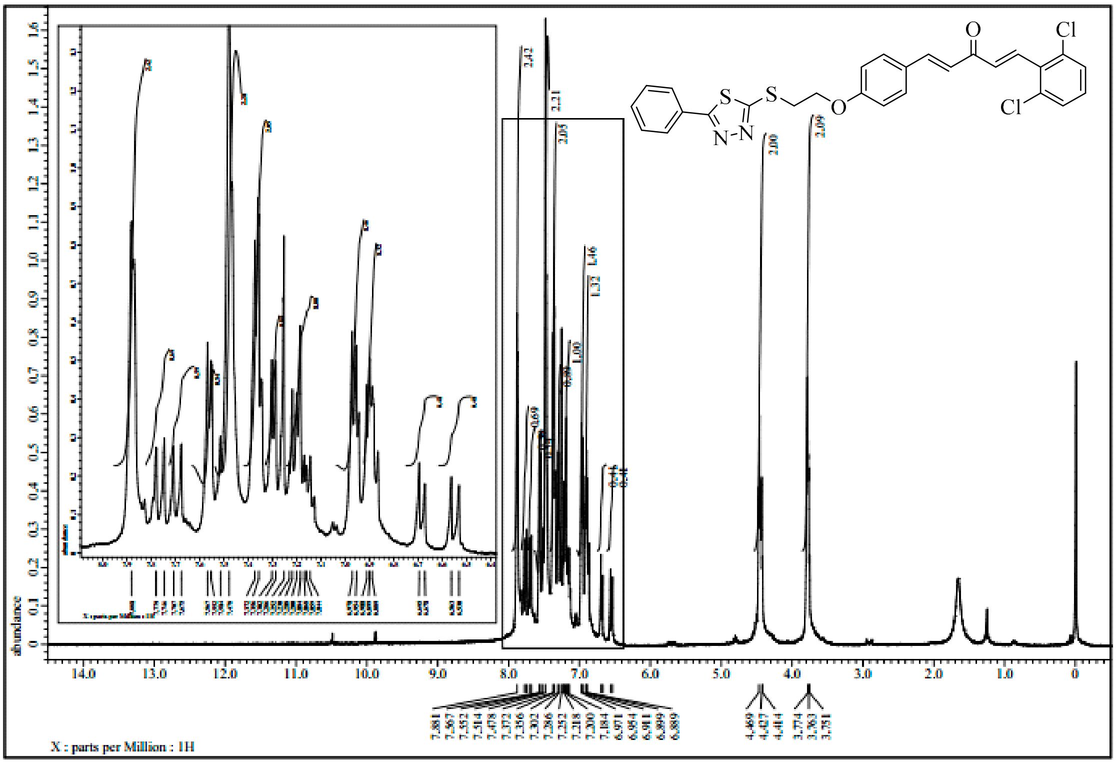

^{1}H NMR of compound **4r**

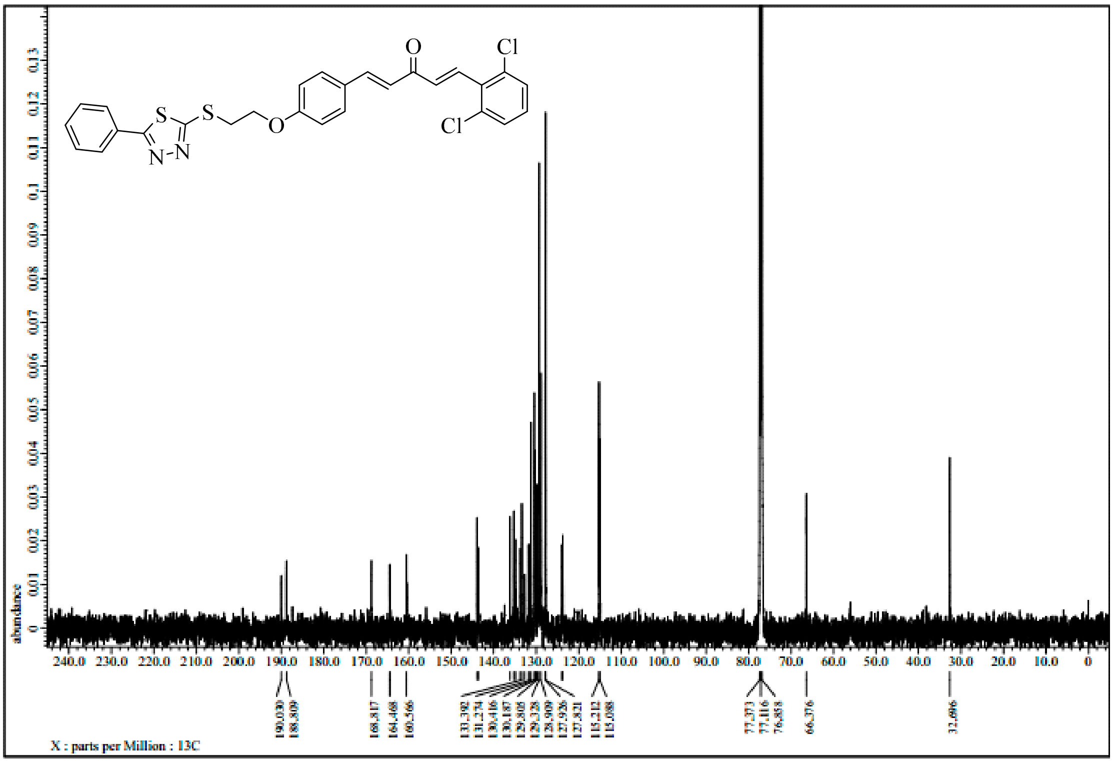

^{13}C NMR of compound **4r**

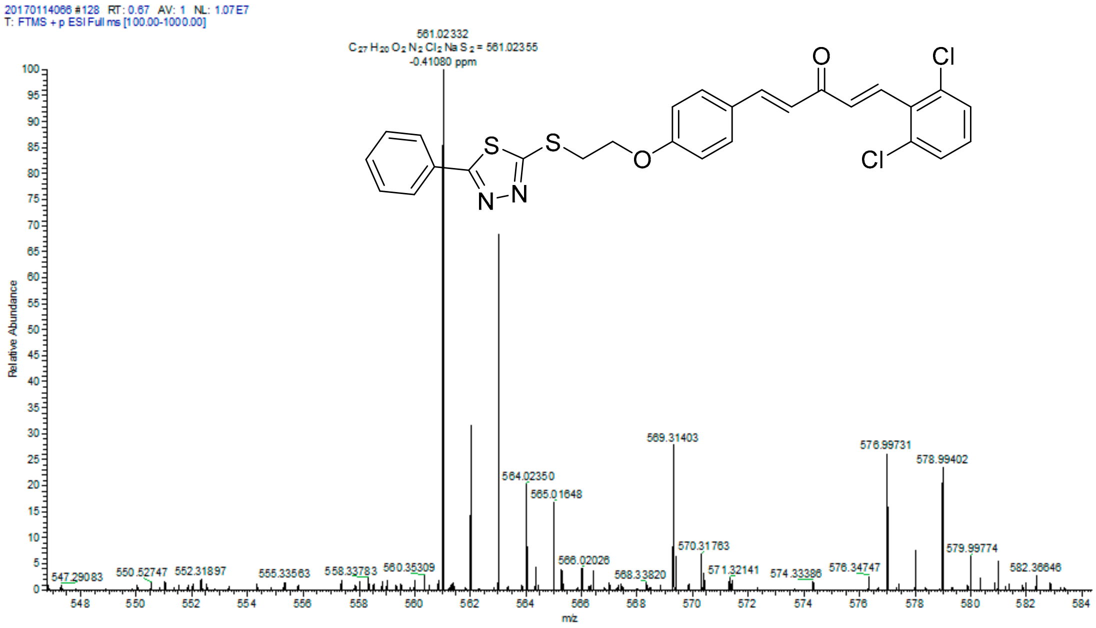

HRMS of compound **4r**

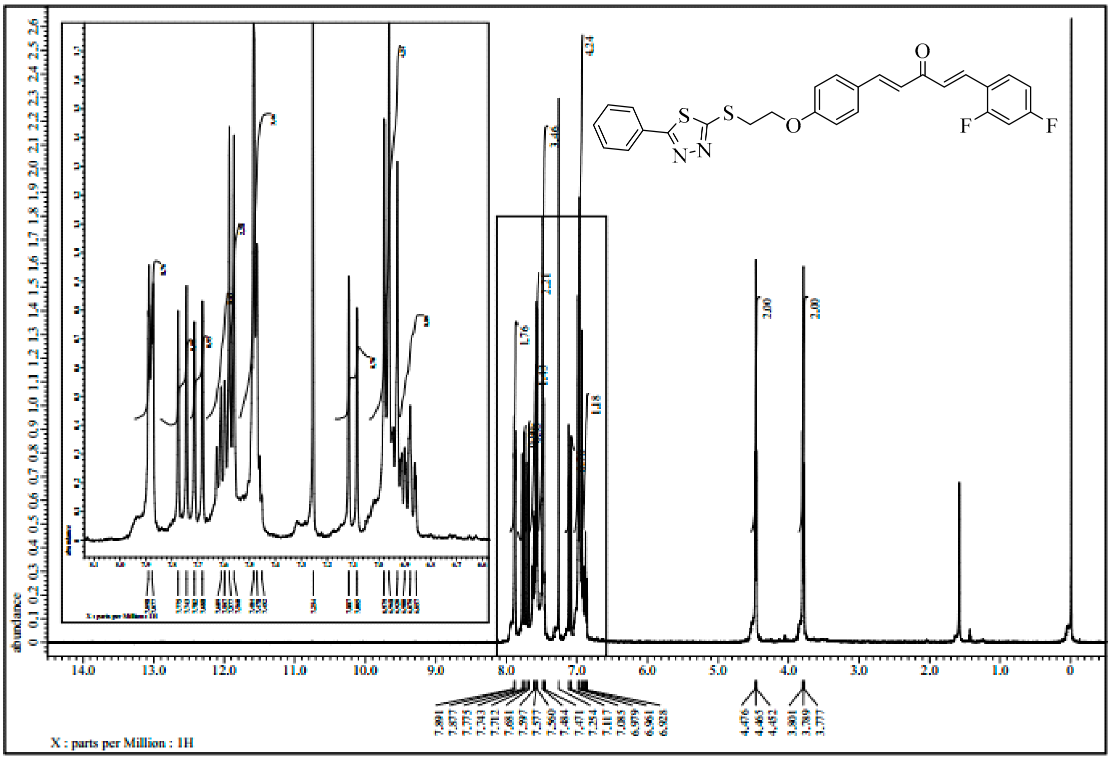

^{1}H NMR of compound **4s**

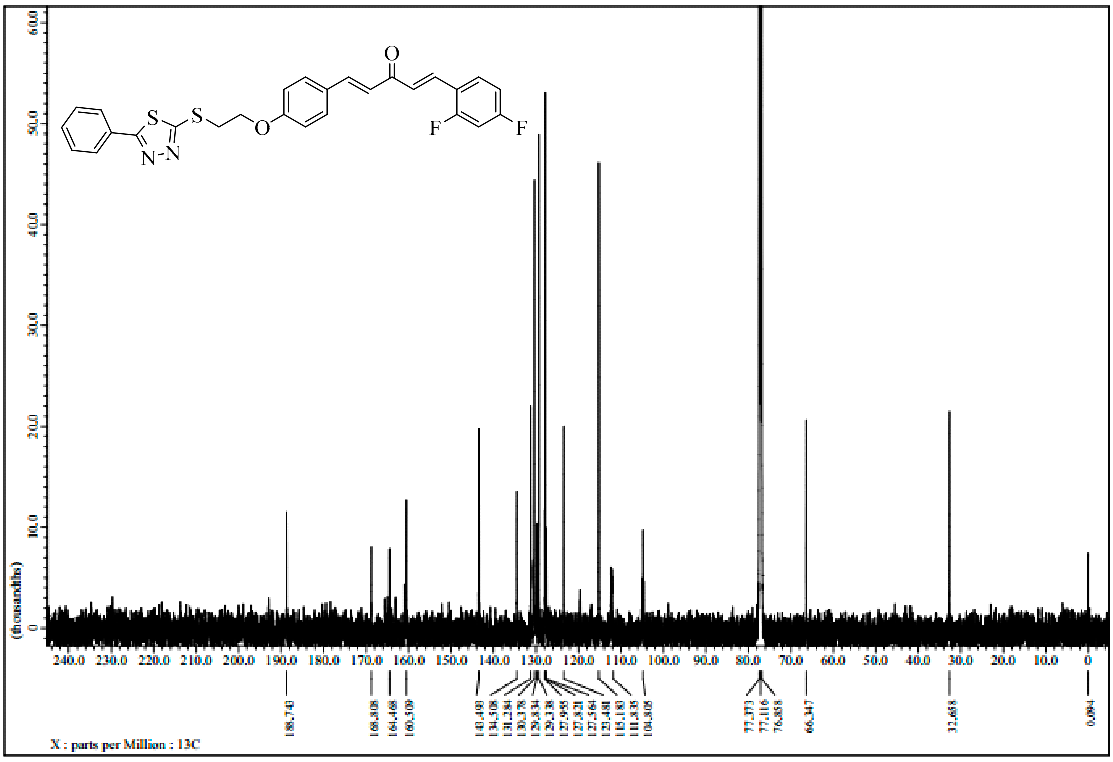

^{13}C NMR of compound **4s**

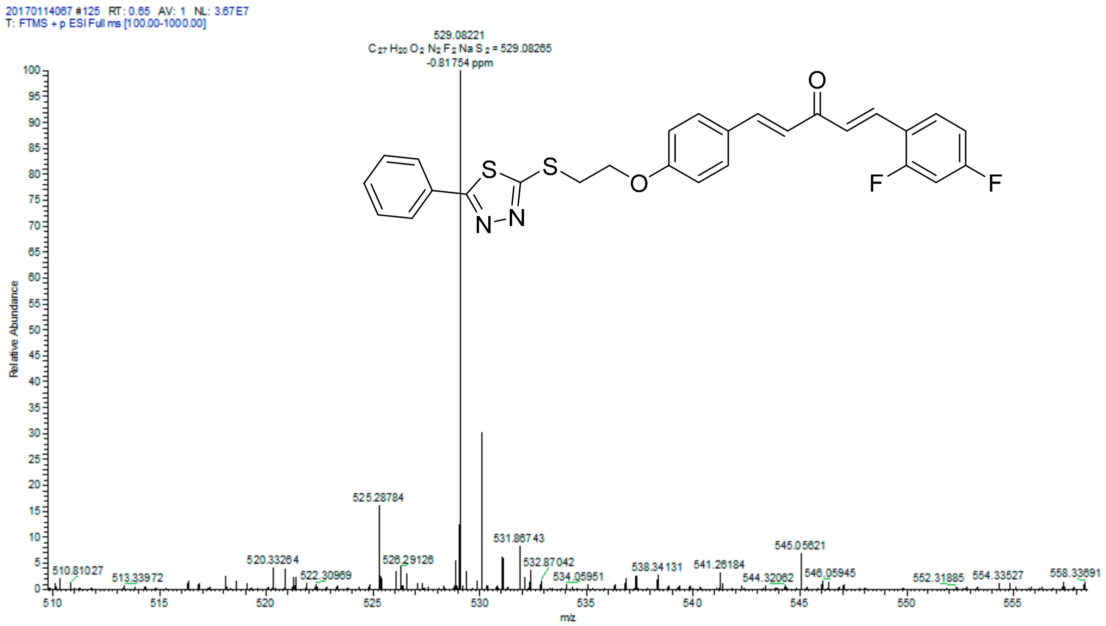

HRMS of compound **4s**

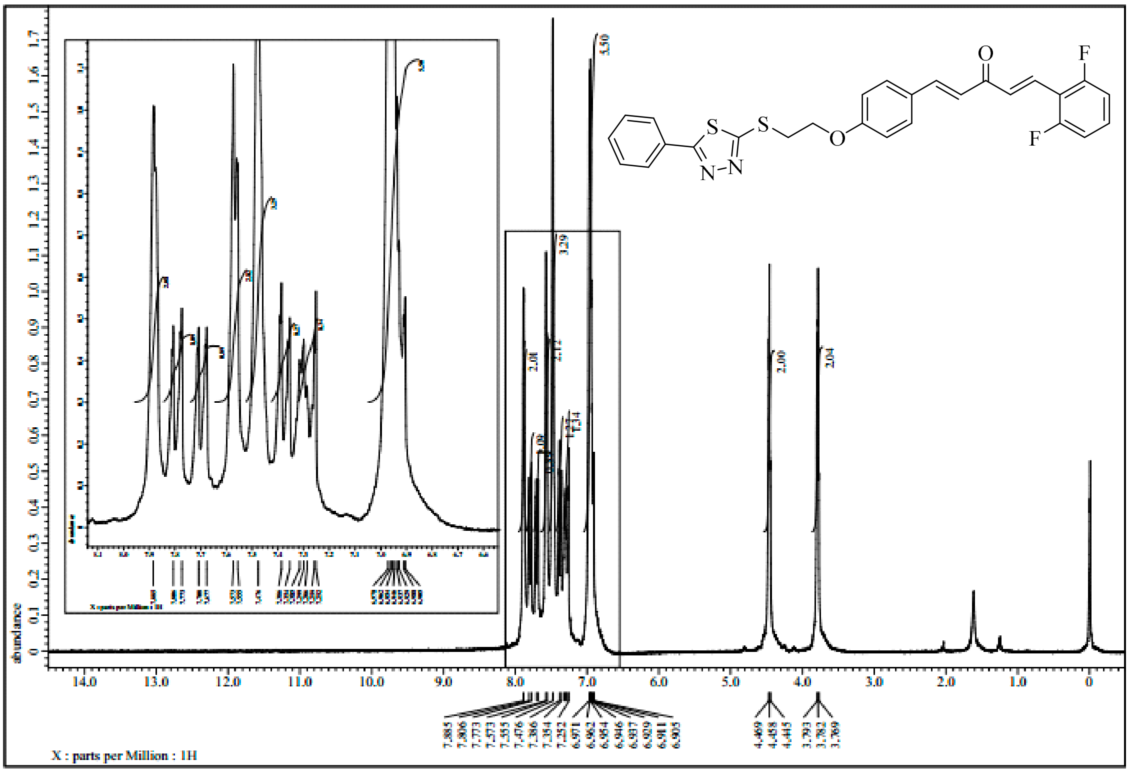

^{1}H NMR of compound **4t**

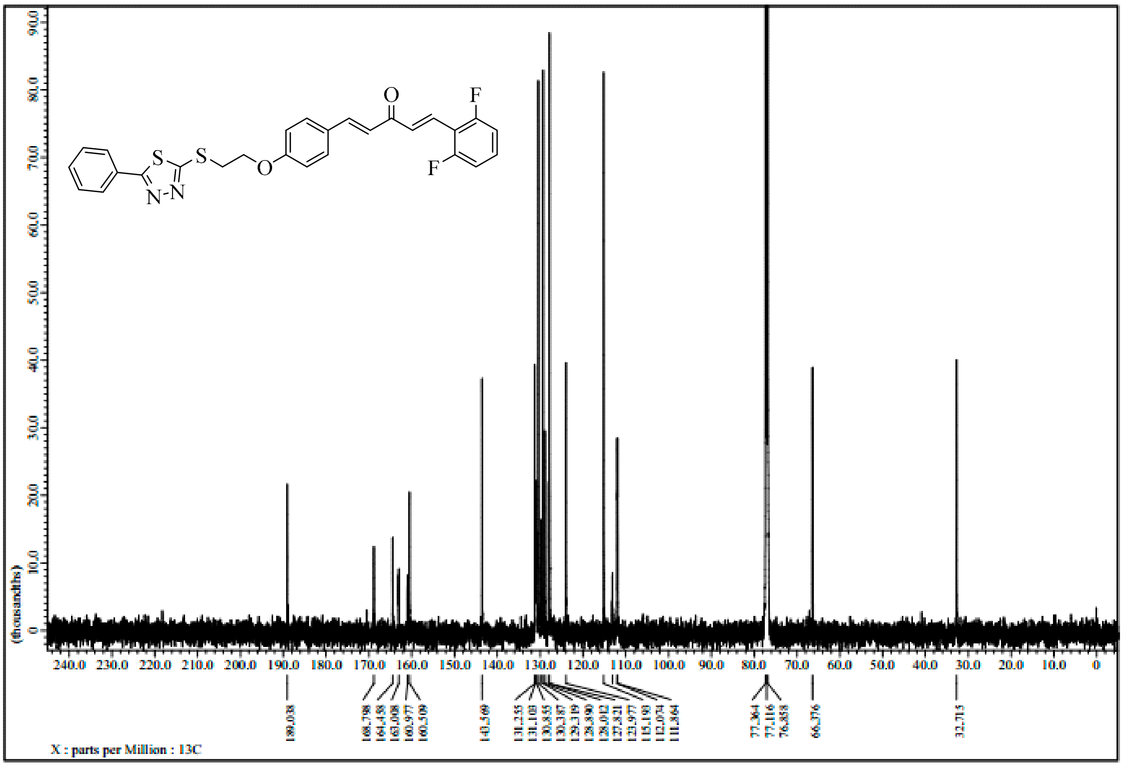

^{13}C NMR of compound **4t**

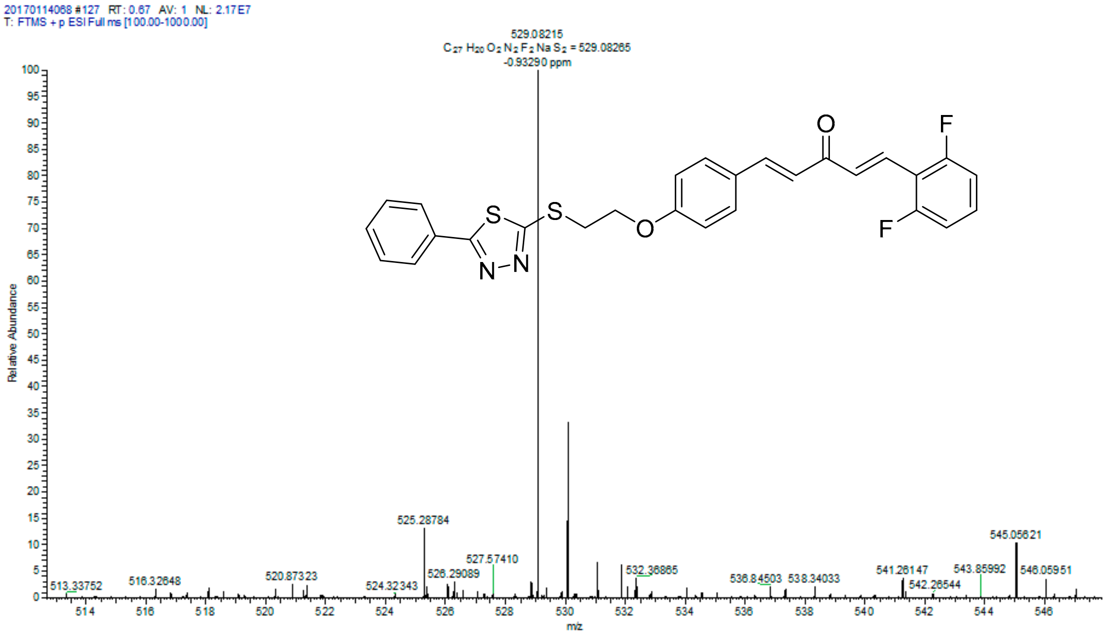

HRMS of compound **4t**
